# Copper-Catalyzed Regioselective Synthesis of (*E*)-β-Fluorovinyl Sulfones

**DOI:** 10.3390/molecules24081569

**Published:** 2019-04-20

**Authors:** Raquel Román, Pablo Barrio, Natalia Mateu, Daniel M. Sedgwick, Santos Fustero

**Affiliations:** 1 Departamento de Química Orgánica, Universitat de València, 46100 Burjassot, Spain; raquel.roman@uv.es; 2 Departamento de Química Orgánica e Inorgánica, Avda. Julián Clavería nº 8, 33006 Oviedo, Spain; barriopablo@uniovi.es; 3 Laboratorio de Moléculas Orgánicas, Centro de Investigación Príncipe Felipe, 46012 Valencia, Spain; natalia.mateu@uv.es

**Keywords:** β-fluorovinyl sulfone, regioselectivity, organofluorine chemistry, copper catalysis, alkynyl sulfones, hydrogenation

## Abstract

Organofluorine compounds are finding increasing application in a variety of fields such as pharmaceutical, agrochemical, and material sciences. However, given the scarcity of fluorine-containing natural products, advancement in this area depends almost entirely on the development of new synthetic methodologies. In this article, we present the synthesis of a series of previously undescribed (*E*)-β-fluorovinyl sulfones via a simple copper-catalyzed addition of hydrogen fluoride to alkynyl sulfone starting materials in varying yields and *E/Z* selectivities. The hydrogenation of these products was also explored and compared with the hydrogenation of the related *Z* isomers. These new products may find interesting applications, given the versatility of vinyl sulfones in chemical synthesis and the unique properties of vinyl fluorides in biological settings.

## 1. Introduction

Vinyl sulfones and related compounds are versatile intermediates that lend themselves to a wide variety of processes such as cycloadditions, hydrogenations, and Michael additions [1,2,3]. Fluorovinyl sulfones and derivatives, therefore, possess strong potential as fluorinated building blocks towards more complex fluorinated organic molecules.

Research into such areas represents a key goal in the synthetic community given the scarcity of fluorine-containing molecules in nature, and the prevalence of this capricious element in pharmaceutical, agrochemical and material sciences [4].

**Scheme 1:**
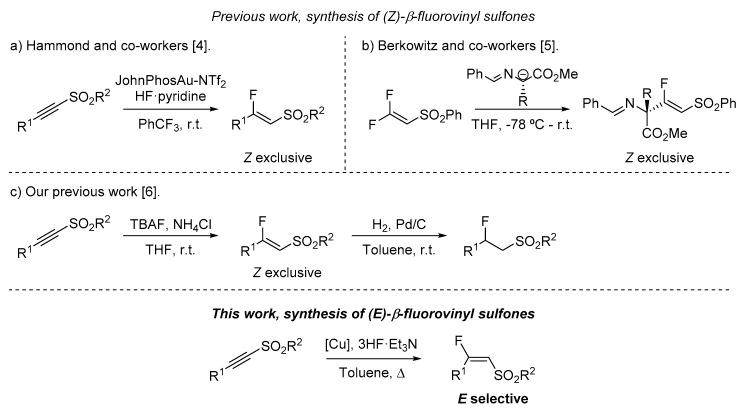
Comparison of past examples dealing with the synthesis of (*Z*)-β-fluorovinyl sulfones and in this work, the synthesis of related *E* isomers.

Although the synthesis of α-fluorovinyl sulfones is well-documented [5,6,7,8,9,10,11,12,13], the synthesis of the corresponding β isomers is much less so, and very few examples exist for the synthesis of such compounds. The first of these was reported just last year when Hammond and co-workers described a gold-catalyzed addition of HF·pyridine to alkynyl sulfone starting materials towards (*Z*)-β-fluorovinyl sulfones (Scheme 1a) [14]. Almost simultaneously, Berkowitz and co-workers described the use of phenyl 2,2-difluorovinyl sulfone as an electrophile in the synthesis of amino acid derivatives containing a (*Z*)-β-fluorovinyl sulfone moiety (Scheme 1b) [15]. This report also contained the first example of downstream chemistry using these products. We then described a metal-free and practical synthesis of (*Z*)-β-fluorovinyl sulfones via the addition of TBAF to alkynyl sulfones, and their subsequent chemoselective hydrogenation to saturated β-fluoroalkyl sulfones (Scheme 1c) [16].

Following our interest in this field, herein we report the synthesis of the related (*E*)-β-fluorovinyl sulfones which, to the best of our knowledge, remain undescribed in the chemical literature (Scheme 1).

## 2. Results and Discussion

We first began this study looking at the metal-catalyzed addition of a variety of fluoride sources to the alkynyl sulfone starting materials used in our previous publication dealing with the synthesis of the *Z* isomers [16]. We were surprised to observe small amounts of the elusive *E* isomers in our crude reaction mixtures, although in most cases with standard gold and silver-based catalysts, the major product was the expected *Z* isomer (Table 1). We then decided to explore the use of copper-based (Ph_3_P)_3_CuF·2MeOH, after Zhu and co-workers described a switch in regioselectivity in the hydrofluorination of ynamides when using this catalyst [17]. To our delight, the *E* isomer was formed to a higher degree when using this catalyst, which we prepared via the method described by Chaudhuri and co-workers [18]. During a short optimization of the reaction conditions, we found that heating the reagents to 70 °C in toluene gave the best results in terms of conversion and selectivity (Entry 7, Table 1). It is worth noting that the conversion was important in this procedure since the starting alkynyl sulfones and the resulting (*E*)-β-fluorovinyl sulfones were somewhat difficult to separate via simple column chromatography. The *E* and *Z* isomers, however, could be separated without any difficulties. A stoichiometric amount of the copper complex could also be used to effectively carry out the reaction (Entry 8, Table 1). Oddly, we found that the commercially available catalyst (Ph_3_P)_3_CuF free of any coordinated methanol was catalytically inactive under the same reaction conditions (Entry 9, Table 1), even though the same copper complex could be used stoichiometrically, albeit with lower stereoselectivity (Entry 10, Table 1). These results suggested that the methanol plays an important role in the stereoselectivity and the regeneration of the catalytically active copper species. The coordinated methanol has also been observed to exert important effects in other reactions catalyzed by this copper complex [19], as well as in other metal-mediated transformations of alkynes [20].

**Scheme 2:**
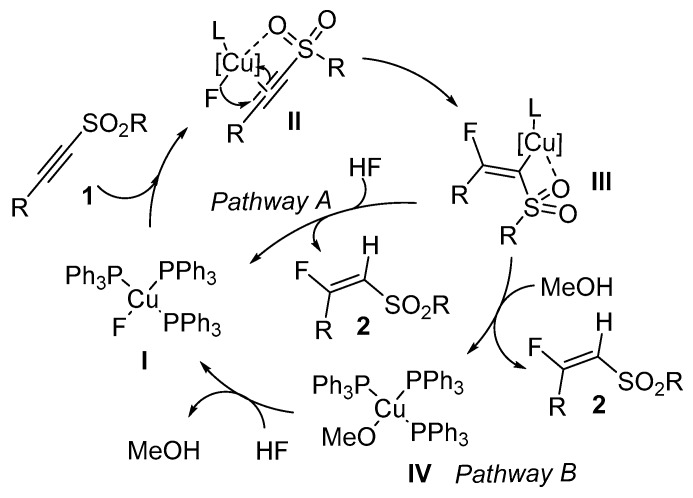
Proposed reaction mechanism.

In terms of the reaction mechanism, we propose the *E* selectivity is principally governed by an interaction between the copper metal center and the oxygen atoms in the sulfone group (Scheme 2). In this first step, the active copper complex **I** coordinates to the triple bond and the sulfone oxygen, forming a four-membered chelate ring in intermediate **II**. From there, the fluorine is delivered in the β position, leaving vinyl cuprate intermediate **III**. From there, one of the two pathways could be acting. Pathway A involves the direct protodemetallation and regeneration of active species **I** through the addition of HF. Pathway B involves the formation of copper species **IV** via methanol-mediated protodemetallation and subsequent regeneration of **I** through the reaction with hydrogen fluoride. We suspect the second pathway is more likely, given that the only slightly acidic 3HF·Et_3_N is used as the hydrogen fluoride source; methanol, therefore, could be preferred for the protodemetallation step. Secondly, this would explain the role of the coordinated methanol in the catalyst, given that the commercially available complex—which contains no methanol—was catalytically inactive, yet successful when a stoichiometric amount was used.

This mechanistic hypothesis was found to be plausible after carrying out the reaction with the catalytically inactive commercial species in a solvent mixture of toluene:methanol (20:1) (Entry 11, Table 1). To our delight, we found that the reaction proceeded to completion, proving that the methanol was indeed necessary for the catalytic reaction to take place and suggesting that the reaction was occurring via pathway B. Furthermore, using deuterated methanol in the same experiment, we saw an incorporation of roughly 50% deuterium into the vinylic position of the product (see supporting information).

**Scheme 3:**
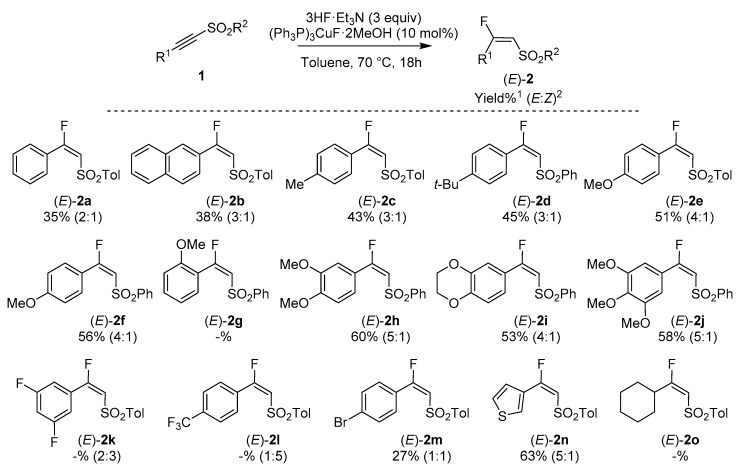
Reaction scope of our *syn*-hydrofluorination procedure. ^1^ Isolated yield. ^2^ Determined by ^19^F-NMR of the crude mixture.

We then proceeded to explore the scope of this reaction (Scheme 3). Substrates bearing electron-neutral and electron-rich aromatic rings were found to be suitable substrates and gave rise to the desired (*E*)-β-fluorovinyl sulfones in moderate to good yields (products **2a**–**j**, Scheme 3). However, substituents in the *ortho* position prevented the reaction from taking place, most likely due to the steric factors given that substrate **1g** bears an electronically favorable methoxy group, and even so the reaction failed to take place. Conversely, alkynyl sulfones bearing aromatic rings containing electron-withdrawing substituents proved less suitable in this procedure, resulting in lower *E* selectivity—or even slight *Z* selectivity—and therefore lower yields (products **2k**–**m**, Scheme 3). Thiophenyl derivative **1n** was also a successful substrate and afforded the desired **2n** in good selectivity and yield. Furthermore, we found that aromatic groups at the triple bond were required for the reaction to proceed successfully; substrate **1o** featuring a cyclohexyl-substituted alkynyl sulfone was found to be unsuitable for this reaction and resulted in a complex mixture of products.

**Figure 1:**
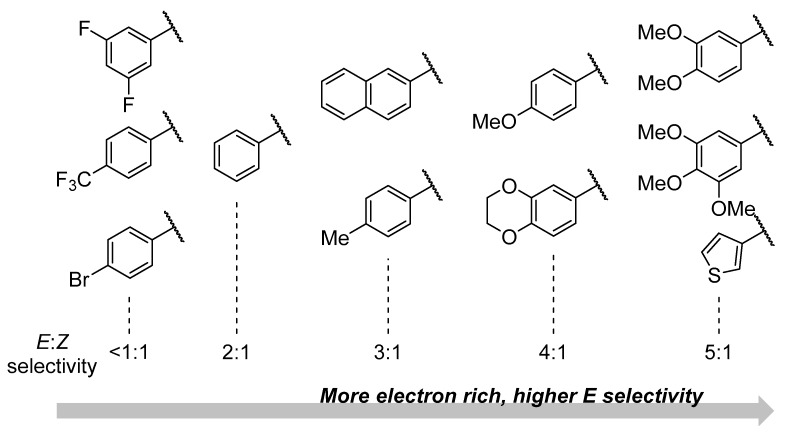
Correlation between the electronic properties of the alkynyl sulfone starting material and the *E*:*Z* selectivity of copper-catalyzed fluoride addition.

We observed a clear trend between the electronic properties of the substrate and the *E*:*Z* selectivity; substrates featuring more electron-rich aromatic rings favored the formation of the desired *E* isomers, whereas the opposite was true for substrates featuring more electron-poor aromatic rings (Figure 1).

We then explored the use of the resulting (*E*)-fluorovinyl sulfones in hydrogenation reactions, given our previous experience in this field [16]. Unfortunately, we found that the *Z* isomers were far more suitable for use in our hydrogenation procedure towards the same saturated fluoroalkyl sulfone products. The *E* isomers described herein were found to be much less reactive and required far longer reaction times to achieve similar rates of conversion, as well as resulting in higher rates of hydrodefluorination (Table 2). This is somewhat different from what one would expect from this type of reaction. Generally speaking, the reaction rate of catalytic heterogeneous hydrogenation correlates directly to the stability of the olefin in question—which in turn usually means the less sterically hindered isomer should react faster [21]. In our case, this would suggest that the (*E*)-β-fluorovinyl sulfones should react faster, which is not what we observed experimentally. Therefore, we could expect the differences to be due to electronic rather than steric factors. These results also reinforced our previous observation that the loss of fluorine during the hydrogenation of fluorovinyl sulfones takes place via the saturated fluoroalkyl sulfone product [16].

## 3. Materials and Methods

### 3.1. General Information

All reactions were carried out under a nitrogen atmosphere unless otherwise indicated. Solvents were purified prior to use: THF and toluene were distilled from sodium and DCM from calcium hydride. Reagents were used as received from the suppliers without further purification unless stated otherwise. The reactions were monitored by TLC on 0.25mm precoated silica-gel plates, which were revealed with UV light and aqueous ceric ammonium molybdate or potassium permanganate stains. Flash column chromatography was performed with the indicated solvents on silica gel 60 (particle size: 0.040−0.063 mm). ^1^H-, ^13^C- and ^19^F-NMR spectra were recorded by a 300 MHz spectrometer. Chemical shifts are given in ppm (δ), referenced to the residual proton resonances of the solvents. Coupling constants (*J*) are given in Hertz (Hz). The letters s, d, t, q and m stand for singlet, doublet, triplet, quartet and multiplet respectively. The letters br indicate that the signal is broad. A QTOF mass analysis system was used for the HRMS measurements.

See Supplementary Materials for the spectra of all new compounds as well as the synthetic procedures used to prepare the alkynyl sulfone starting materials.

### 3.2. General Method for the Synthesis of (E)-Fluorovinyl Sulfones

A screw-top eppendorf tube was charged with alkynyl sulfone (1 equiv) and (Ph_3_P)_3_CuF·2MeOH (10 mol%), and purged with nitrogen. Toluene (0.05 M) was then added, followed by 3HF·Et_3_N (3 equiv), and the resulting mixture was heated at 70 °C for 20 h in an oil bath. When the reaction was complete, the crude mixture was concentrated and purified by the flash column chromatography using mixtures of hexane and ethyl acetate as the eluent (assuming the reaction was complete; if not, mixtures of hexane and DCM were used to separate the product from the alkynyl sulfone starting material).

### 3.3. Characterization of (E)-Fluorovinyl Sulfones ***2a***–***2n***

*(E)-1-((2-Fluoro-2-phenylvinyl)sulfonyl)-4-methylbenzene* ((*E*)-**2a**). Flash chromatography of the crude reaction product [*n*-hexane:EtOAc (4:1)] afforded (*E*)-**2a** as a colorless oil (35%, 65 mg). ^1^H-NMR (CDCl_3_, 300 MHz): δ 2.33 (s, 3H), 6.38 (d, *J* = 18.4 Hz, 1H), 7.16–7.19 (m, 2H), 7.36–7.38 (m, 2H), 7.38–7.43 (m, 1H), 7.54–7.59 (m, 4H) ppm. ^13^C-NMR (CDCl_3_, 75.5 MHz): 21.6, 114.5 (d, *J* = 31.8 Hz), 127.4, 128.1 (d, *J* = 16.3 Hz), 128.3, 129.5 (d, *J* = 5.0 Hz), 129.6, 129.7 (d, *J* = 2.2 Hz), 132.0, 138.5 (d, *J* = 2.9 Hz), 138.6, 144.5, 167.7 (d, *J* = 276.4 Hz) ppm. ^19^F-NMR (CDCl_3_, 282.4 MHz): δ -71.74 (d, *J* = 18.4 Hz, 1F) ppm. HRMS (EI) calcd. for C_15_H_14_FO_2_S [M + H^+^]: 277.0693, found: 277.0698.

*(E)-2-(1-Fluoro-2-tosylvinyl)naphthalene* ((*E*)-**2b**). Flash chromatography of the crude reaction product [*n*-hexane:EtOAc (5:1)] afforded (*E*)-**2b** as a pale yellow solid (38%, 16 mg) with a melting point of 42–43 °C. ^1^H-NMR (CDCl_3_, 300 MHz): δ 2.29 (s, 3H), 6.47 (d, *J* = 18.4 Hz, 1H), 7.10–7.13 (d, 2H), 7.49–7.57 (m, 5H), 7.77–7.86 (m, 3H), 8.15 (s, 1H) ppm. ^13^C-NMR (CDCl_3_, 75.5 MHz): δ 21.6, 114.7 (d, *J* = 32.0 Hz), 124.9 (d, *J* = 3.8 Hz), 125.6 (d, *J* = 25.7 Hz), 126.9, 127.3, 127.4, 127.8 (d, *J* = 8.2 Hz), 128.3, 129.1, 129.7, 131.2 (d, *J* = 6.5 Hz), 132.0, 134.7, 138.5, 144.5, 167.7 (d, *J* = 276.6 Hz) ppm. ^19^F-NMR (CDCl_3_, 282.4 MHz): δ −71.93 (d, *J* = 18.4 Hz, 1F) ppm. HRMS (EI) calcd. for C_19_H_19_FNO_2_S [M + NH_4_^+^]: 344.1115, found: 344.1120.

*(E)-1-((2-Fluoro-2-(4-tolyl)vinyl)sulfonyl)-4-methylbenzene* ((*E*)-**2c**). Flash chromatography of the crude reaction product [*n*-hexane:EtOAc (4:1)] afforded (*E*)-**2c** as a colorless oil (43%, 18 mg). ^1^H-NMR (CDCl_3_, 300 MHz): δ 2.44 (s, 6H), 6.40 (d, *J* = 18.5 Hz, 1H), 7.25–7.30 (m, 4H), 7.58–7.60 (d, 2H), 7.67–7.69 (d, 2H) ppm. ^13^C-NMR (CDCl_3_, 75.5 MHz): δ 21.6, 21.7, 113.7 (d, *J* = 32.6 Hz), 125.6 (d, *J* = 26.0 Hz), 127.3, 128.8, 129.6 (d, *J* = 5.6 Hz), 129.7, 138.7 (d, *J* = 2.7 Hz), 142.7, 144.4, 167.8 (d, *J* = 275.7 Hz) ppm. ^19^F-NMR (CDCl_3_, 282.4 MHz): δ −72.04 (d, *J* = 18.6 Hz, 1F) ppm. HRMS (EI) calcd. for C_16_H_19_FNO_2_S [M + NH_4_^+^]: 308.1115, found: 308.1116.

*(E)-1-(tert-Butyl)-4-(1-fluoro-2-(phenylsulfonyl)vinyl)benzene* ((*E*)-**2d**). Flash chromatography of the crude reaction product [*n*-hexane:EtOAc (4:1)] afforded (*E*)-**2d** as a colorless oil (45%, 19 mg). ^1^H-NMR (CDCl_3_, 300 MHz): δ 1.36 (s, 9H), 6.45 (d, *J* = 18.4 Hz, 1H), 7.43–7.48 (m, 5H), 7.55–7.62 (m, 2H), 7.76–7.79 (m, 2H) ppm. ^13^C-NMR (CDCl_3_, 75.5 MHz): δ 31.1, 35.1, 113.7 (d, *J* = 32.9 Hz), 125.1, 125.2, 127.4, 128.6 (d, *J* = 11.8 Hz), 129.0, 129.4 (d, *J* = 5.2 Hz), 133.3 (d, *J* = 10.3 Hz), 141.5, 155.8, 168.2 (d, *J* = 276.3 Hz) ppm. ^19^F-NMR (CDCl_3_, 282.4 MHz): δ −71.61 (d, *J* = 18.4 Hz, 1F) ppm. HRMS (EI) calcd. for C_18_H_23_FNO_2_S [M + NH_4_^+^]: 336.1428, found: 336.1425.

*(E)-1-((2-Fluoro-2-(4-methoxyphenyl)vinyl)sulfonyl)-4-methylbenzene* ((*E*)-**2e**). Flash chromatography of the crude reaction product [*n*-hexane:EtOAc (3:1)] afforded (*E*)-**2e** as a colorless oil (51%, 30 mg). ^1^H-NMR (CDCl_3_, 300 MHz): δ 2.33 (s, 3H), 3.79 (s, 3H), 6.26 (d, *J* = 18.8 Hz, 1H), 6.86 (dd, *J* = 9.1, 0.9 Hz, 2H), 7.17–7.20 (m, 2H), 7.65 (s, 2H), 7.58–7.61 (m, 4H) ppm. ^13^C-NMR (CDCl_3_, 75.5 MHz): δ 21.6, 55.5, 112.7 (d, *J* = 33.5 Hz), 113.5, 120.6 (d, *J* = 26.7 Hz), 127.3, 128.5, 128.7, 129.7, 131.6 (d, *J* = 5.8 Hz), 133.8 (d, *J* = 19.4 Hz), 138.8 (d, *J* = 3.0 Hz), 144.4, 162.6 (d, *J* = 1.5 Hz), 167.4 (d, *J* = 274.4 Hz) ppm. ^19^F-NMR (CDCl_3_, 282.4 MHz): δ −72.67 (d, *J* = 18.8 Hz, 1F) ppm. HRMS (EI) calcd. for C_16_H_15_FO_3_S [M + H^+^]: 307.0799, found: 307.0803.

*(E)-1-(1-Fluoro-2-(phenylsulfonyl)vinyl)-4-methoxybenzene* ((*E*)-**2f**). Flash chromatography of the crude reaction product [*n*-hexane:EtOAc (3:1)] afforded (*E*)-**2f** as a colorless oil (56%, 40 mg). ^1^H-NMR (CDCl_3_, 300 MHz): δ 3.86 (s, 3H), 6.35 (d, *J* = 18.6 Hz, 1H), 6.93 (d, *J* = 8.4 Hz, 2H), 7.38–7.50 (m, 2H), 7.51–7.61 (m, 1H), 7.66 (d, *J* = 8.6 Hz, 2H), 7.78 (dd, *J* = 5.3, 3.3 Hz, 2H) ppm. ^13^C-NMR (CDCl_3_, 75.5 MHz): δ 55.5, 112.4 (d, *J* = 33.6 Hz), 113.6, 120.5 (d, *J* = 26.6 Hz), 127.2, 129.1, 131.5 (d, *J* = 5.6 Hz), 133.4, 141.7 (d, *J* = 2.5 Hz), 162.7, 167.8 (d, *J* = 275.1 Hz) ppm. ^19^F-NMR (CDCl_3_, 282.4 MHz): δ −71.77 (d, *J* = 18.6 Hz, 1F) ppm. HRMS (EI) calcd. for C_15_H_17_FNO_3_S [M + NH_4_^+^]: 310.0908, found: 310.0911.

*(E)-4-(1-Fluoro-2-(phenylsulfonyl)vinyl)-1,2-dimethoxybenzene* ((*E*)-**2h**). Flash chromatography of the crude reaction product [*n*-hexane:EtOAc (2:1)] afforded (*E*)-**2h** as a white solid (60%, 24 mg) with a melting point of 51–53 °C. ^1^H-NMR (CDCl_3_, 300 MHz): δ 3.91 (s, 3H), 3.95 (s, 3H), 6.39 (d, *J* = 18.8 Hz, 1H), 6.92 (d, *J* = 8.4 Hz, 1H), 7.27 (d, *J* = 2.1 Hz, 1H), 7.34 (ddd, *J* = 8.4, 2.0, 0.6 Hz, 1H), 7.45–7.50 (m, 2H), 7.56–7.58 (m, 1H), 7.78–7.81 (m, 2H) ppm. ^13^C-NMR (CDCl_3_, 75.5 MHz): δ 56.0, 110.3, 112.2 (d, *J* = 5.4 Hz), 112.7 (d, *J* = 33.7 Hz), 120.5 (d, *J* = 26.8 Hz), 123.7 (d, *J* = 6.5 Hz), 127.2, 129.1, 133.4, 141.6 (d, *J* = 2.5 Hz), 148.4, 152.4, 167.5 (d, *J* = 275.0 Hz) ppm. ^19^F-NMR (CDCl_3_, 282.4 MHz): δ −72.68 (d, *J* = 18.9 Hz, 1F) ppm. HRMS (EI) calcd. for C_16_H_19_FNO_4_S [M + NH_4_^+^]: 340.1013, found: 340.1019.

*(E)-6-(1-Fluoro-2-(phenylsulfonyl)vinyl)-2,3-dihydrobenzo[b]*[1,4]*dioxine* ((*E*)-**2i**). Flash chromatography of the crude reaction product [*n*-hexane:EtOAc (2:1)] afforded (*E*)-**2i** as a colorless oil (53%, 19 mg). ^1^H-NMR (CDCl_3_, 300 MHz): δ 4.22 (dqd, *J* = 7.0, 3.3, 1.5 Hz, 4H), 6.28 (d, *J* = 18.6 Hz, 1H), 6.82 (dd, *J* = 8.4, 0.8 Hz, 1H), 7.12–7.14 (m, 2H), 7.40–7.43 (m, 2H), 7.49–7.51 (m, 1H), 7.72–7.75 (m, 2H) ppm. ^13^C-NMR (CDCl_3_, 75.5 MHz): δ 64.1, 64.6, 112.8 (d, *J* = 33.5 Hz), 117.1, 118.8 (d, *J* = 5.5 Hz), 121.2 (d, *J* = 26.6 Hz), 123.6 (d, *J* = 5.9 Hz), 127.4, 129.1, 133.4, 141.6, 143.0, 147.1, 167.3 (d, *J* = 275.8 Hz) ppm. ^19^F-NMR (CDCl_3_, 282.4 MHz): δ −71.81 (d, *J* = 18.6 Hz, 1F) ppm. HRMS (EI) calcd. for C_16_H_17_FNO_4_S [M + NH_4_^+^]: 338.0857, found: 338.0866.

*(E)-5-(1-Fluoro-2-(phenylsulfonyl)vinyl)-1,2,3-trimethoxybenzene* ((*E*)-**2j**). Flash chromatography of the crude reaction product [*n*-hexane:EtOAc (2:1)] afforded (*E*)-**2j** as a colorless oil (58%, 23 mg). ^1^H-NMR (CDCl_3_, 300 MHz): δ 3.88 (s, 6H), 3.93 (s, 3H), 6.46 (d, *J* = 18.6 Hz, 1H), 6.96 (s, 2H), 7.45–7.50 (m, 2H), 7.56–7.59 (m, 1H), 7.76–7.80 (m, 2H) ppm. ^13^C-NMR (CDCl_3_, 75.5 MHz): δ 56.3, 61.0, 107.1 (d, *J* = 5.6 Hz), 113.9 (d, *J* = 32.9 Hz), 123.0 (d, *J* = 26.8 Hz), 127.2, 128.6 (d, *J* = 8.2 Hz), 129.0, 133.4, 133.8 (d, *J* = 13.9 Hz), 141.4, 152.7, 167.4 (d, *J* = 276.5 Hz) ppm. ^19^F-NMR (CDCl_3_, 282.4 MHz): δ −73.03 (d, *J* = 18.6 Hz, 1F) ppm. HRMS (EI) calcd. for C_17_H_21_FNO_5_S [M + NH_4_^+^]: 370.1119, found: 370.1123.

*(E)-1-Bromo-4-(1-fluoro-2-tosylvinyl)benzene* ((*E*)-**2m**). Flash chromatography of the crude reaction product [*n*-hexane:EtOAc (5:1)] afforded (*E*)-**2m** as a colorless oil (27%, 13mg). ^1^H-NMR (CDCl_3_, 300 MHz): δ 2.42 (s, 3H), 6.45 (d, *J* = 18.5 Hz, 1H), 7.26–7.30 (m, 2H), 7.52–7.66 (m, 6H) ppm. ^13^C-NMR (CDCl_3_, 75.5 MHz): δ 21.6, 114.9 (d, *J* = 31.3 Hz), 127.0, 127.1, 127.4, 129.8, 131.0 (d, *J* = 4.9 Hz), 131.4, 138.3, 144.8, 166.27 (d, *J* = 275.7 Hz) ppm. ^19^F-NMR (CDCl_3_, 282.4 MHz): δ −73.51 (d, *J* = 18.5 Hz, 1F) ppm. HRMS (EI) calcd. for C_15_H_16_BrFNO_2_S [M + NH_4_^+^]: 372.0064, found: 372.0068.

*(E)-3-(1-Fluoro-2-tosylvinyl)thiophene* ((*E*)-**2n**). Flash chromatography of the crude reaction product [*n*-hexane:EtOAc (4:1)] afforded (*E*)-**2n** as a colorless oil (63%, 23 mg). ^1^H-NMR (CDCl_3_, 300 MHz): δ 2.34 (s, 3H), 6.28 (d, *J* = 20.4 Hz, 1H), 7.22–7.25 (m, 3H), 7.43–7.45 (m, 1H), 7.63–7.65 (m, 2H), 8.12 (ddd, *J* = 3.0, 1.3, 0.7 Hz, 1H) ppm. ^13^C-NMR (CDCl_3_, 75.5 MHz): δ 21.6, 112.7 (d, *J* = 33.1 Hz), 125.9, 127.2, 127.5 (d, *J* = 5.0 Hz), 129.8, 132.3 (d, *J* = 8.3 Hz), 138.7 (d, *J* = 2.8 Hz), 144.6, 162.4 (d, *J* = 269.0 Hz) ppm. ^19^F-NMR (CDCl_3_, 282.4 MHz): δ −77.29 (dd, *J* = 20.5, 1.3 Hz, 1F) ppm. HRMS (EI) calcd. for C_15_H_13_FO_2_S [M + H^+^]: 283.0257, found: 283.0257.

## 4. Conclusions

In conclusion, we have developed a copper-catalyzed procedure to synthesize the previously undescribed (*E*)-β-fluorovinyl sulfones starting from the parent alkynyl sulfones. The hydrogenation of these products was also explored, and unfortunately, the *E* isomers were much less suitable to this transformation than the *Z* isomers. Further studies into the reactivity of this interesting class of compounds are ongoing in our research group.

## Figures and Tables

**Table 1:**
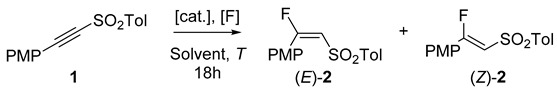
Optimization of the *syn*-hydrofluorination procedure towards (*E*)-β-fluorovinyl sulfones ^1^.

Entry	[cat.]	[F]	Solvent	*T* °C	Conv.% ^2^	*E:Z* ^3^
1	Ph_3_PAuMe	HF·DMPU	DCE	70	44	<1:20
2	JohnPhosAuCl	HF·DMPU	DCE	55	58	1:9
3	AgNTf_2_	3HF·Et_3_N	DMF	70	55	1:5
4 ^4^	(Ph_3_P)_3_CuF·2MeOH	HF·DMPU	THF	70	73	1:1
5 ^4^	(Ph_3_P)_3_CuF·2MeOH	3HF·Et_3_N	THF	70	79	2:1
6 ^4^	(Ph_3_P)_3_CuF·2MeOH	3HF·Et_3_N	Dioxane	70	90	3:1
7 ^4^	(Ph_3_P)_3_CuF·2MeOH	3HF·Et_3_N	Toluene	70	>95	4:1
8 ^4,5^	(Ph_3_P)_3_CuF·2MeOH	-	Toluene	70	>95	3:1
9 ^6^	(Ph_3_P)_3_CuF	3HF·Et_3_N	Toluene	70	<10	- ^7^
10 ^5,6^	(Ph_3_P)_3_CuF	-	Toluene	70	>95	1:1
11 ^8^	(Ph_3_P)_3_CuF	3HF·Et_3_N	Toluene	70	>95	3:1

^1^ Typical reaction conditions: **1** (0.1 mmol), [cat.] (0.01 mmol), [F] (0.3 mmol), solvent (2 mL). ^2^ Determined by ^1^H-NMR of the crude reaction mixture. ^3^ Determined by ^19^F-NMR of the crude reaction mixture. ^4^ Catalyst synthesized as described by Chaudhuri and co-workers [8]. ^5^ 100 mol% of the corresponding copper species was used. ^6^ Commercial catalyst was used as supplied. ^7^ Ratio not determined. ^8^ A mixture of toluene:methanol (20:1) was used as the solvent.

**Table 2:**
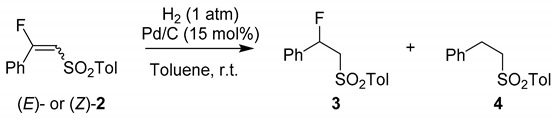
A comparison of the hydrogenation of both (*E*)- and (*Z*)-fluorovinyl sulfones.

Entry	Substrate	Time, h	Conv.% ^1^	3:4 ^1^
1	(*Z*)-2a ^2^	2	>98	12:1
2	(*E*)-2a	2	-	-
3	(*E*)-2a	4	10	1:1
4	(*E*)-2a	20	95	2:3

^1^ Determined by ^1^H-NMR of the crude reaction mixture. ^2^ Synthesized as described in our previous report [16].

## Supplementary Materials

Supplementary Materials are available online.
