# Supplementary material for: Copper-Catalyzed Regioselective Synthesis of (E)-β-Fluorovinyl Sulfones
Source: Molecules. 2019 Apr 20;24(8):1569. doi: 10.3390/molecules24081569 (PMC6514952; doi:10.3390/molecules24081569)
Supplement: Supplementary file 1 [file molecules-24-01569-s001.pdf]

Copper-catalyzed regioselective synthesis of (*E*)-β-fluorovinyl sulfones

Raquel Román[1], Pablo Barrio[2], Natalia Mateu[3], Daniel M. Sedgwick*[1], and Santos Fustero*[1,3]

[1] Departamento de Química Orgánica, Universitat de València, 46100 Burjassot, Spain; raquel.roman@uv.es (R. R.)

[2] Departamento de Química Orgánica e Inorgánica, Avda. Julián Clavería nº 8, 33006 Oviedo, Spain; barriopablo@uniovi.es (P. B.)

[3] Laboratorio de Moléculas Orgánicas, Centro de Investigación Príncipe Felipe, 46012 Valencia, Spain; natalia.mateu@uv.es (N. M.)

***** Correspondence: dased@uv.es (D. M. S); santos.fustero@uv.es (S. F.) Tel.: +34-963544940 (D. M. S.); +34-963544279 (S. F.).

Contents

General 1

Synthesis of starting materials 2

General procedure for the synthesis of (E)-β-fluorovinyl sulfones.. 5

Identification of E/Z isomers of fluorovinyl sulfone products 11

^{1}H NMR of control experiment with CD_3OD.. 12

NMR spectra of new compounds.. 12

General

All reactions were carried out under a nitrogen atmosphere unless otherwise indicated. Solvents were purified prior to use: THF and toluene were distilled from sodium and DCM from calcium hydride. Reagents were used as received from the suppliers without further purification, unless stated otherwise. The reactions were monitored by TLC on 0.25mm precoated silica-gel plates, which were revealed with UV light and aqueous ceric ammonium molybdate or potassium permanganate stains. Flash column chromatography was performed with the indicated solvents on silica gel 60 (particle size: 0.040−0.063 mm). ^{1}H, ^{13}C and ^{19}F NMR spectra were recorded by a 300 MHz spectrometer. Chemical shifts are given in ppm (δ), referenced to the residual proton resonances of the solvents. Coupling constants (*J*) are given in Hertz (Hz). The letters s, d, t, q and m stand for singlet, doublet, triplet, quartet and multiplet respectively. The letters br indicate that the signal is broad. A QTOF mass analysis system was used for the HRMS measurements.

Synthesis of starting materials

*General procedure for alkynyl sulfone synthesis via method **A**.*

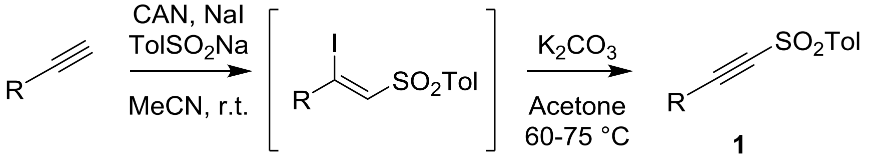

Ceric ammonium nitrate (2.5 equiv) was added portion-wise to a mixture of the corresponding acetylene (1 equiv), sodium *p*-toluenesulfinate (1.2 equiv) and NaI (1.2 equiv) in MeCN (0.08 M) under a nitrogen atmosphere. After the completion of the reaction (generally 1-3 h), water was added and the reaction mixture was extracted with DCM. The organic layer was separated, washed with brine, dried over anhydrous Na_2SO_4, filtered and concentrated to dryness. The residue was then redissolved in anhydrous acetone (0.1 M) and heated at 60-75 °C with K_2CO_3 (3 equiv) for 16 h. After the completion of the reaction, water was added and the reaction mixture was extracted once again with DCM. The combined organic extracts were washed with brine and dried over anhydrous Na_2SO_4. The solvent was removed *in vacuo* using a rotary evaporator and the residue was purified by column chromatography to afford the desired product. Compounds **1c** and **1e** have been previously described by Nair *et al.*[1]; compounds **1a** by Zhu, Jiang *et al.*[2]; compounds **1b**, **1m**, and **1l** by Kuhakarn *et al.*[3]; compound **1n** by Wei, Wang *et al.*[4]; and compounds **1g** and **1o** by Schwarz and König.[5] We described the synthesis of compound **1k** in our previous report.[6]

*General procedure for alkynyl sulfone synthesis via method **B**.*

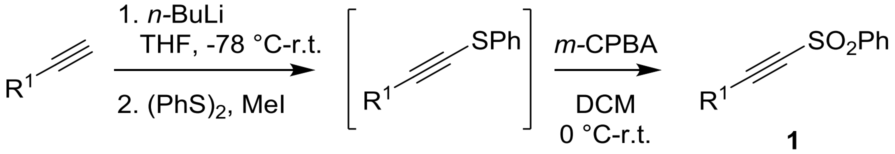

The terminal alkyne starting material (1 equiv) was dissolved in THF (0.1 M) and cooled to -78 °C, and *n*-BuLi (2.5 M in hexanes, 1.1 equiv) was added slowly. The resulting mixture was stirred at this temperature for 1 h, and then removed from the acetone/dry ice bath and stirred for a further 30 minutes whilst slowly warming to room temperature. Phenyl disulfide (1.2 equiv) and iodomethane (1.2 equiv) were then added, and the mixture was stirred for 6 h

[1] Nair, V.; Augustine, A.; Suja, T. D. *Synthesis*, **2002**, *15*, 2259.
[2] Chen, P.; Zhu, C. H.; Zhu, R.; Wu, W.; Jiang, H. *Chem. Asian. J.* **2017**, *12*, 1875.
[3] Meesin, J.; Katrun, P.; Pareseecharoen, C.; Pohmakotre, M.; Reutrakul, V.; Soorukram D.; Kuhakarn, C. *J. Org. Chem.,* **2016**, *81*, 2744.
[4] Wang, L.; Wei, W.; Yang, D.; Cui, H.; Yue, H.; Wang, H. *Tetrahedron Lett.*, **2017**, *58*, 4799.
[5] Schwarz. J; König, B. *ChemPhotoChem*, **2017**, *1*, 237.
[6] Sedgwick, D. M.; Román, R.; Barrio, P.; Morales, C.; Fustero, S. *J. Fluorine Chem.* **2018**, *206*, 108.

at room temperature. After this time, a saturated aqueous NH_4Cl solution was added to quench the reaction, and the resulting biphasic mixture was extracted with diethyl ether. The combined organic layers were dried over anhydrous Na_2SO_4, concentrated *in vacuo*, and the resulting residue was submitted to high vacuum to remove the phenyl methyl sulfide side-product. The crude mixture was dissolved in DCM (0.1 M) and cooled to 0 °C. *m*-CPBA (3 equiv) was then added portion-wise, ensuring that the reaction mixture didn't heat up excessively. The resulting mixture was stirred at 0 °C for 1 h and then a further 12 h at room temperature. After this time, a saturated aqueous solution of sodium bisulfite was added, along with further water. The organic phase was washed successively with further sodium bisulfite solution, saturated aqueous sodium bicarbonate solution, and brine. The resulting organic layer was dried over anhydrous Na_2SO_4, concentrated *in vacuo* and purified by flash column chromatography. Compound **1f** has been previously described by Nair *et al.*[1], whereas compounds **1h** and **1i** have been described by Pelter, Ward *et al.*[7]

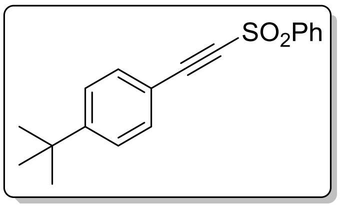

1-(*tert*-Butyl)-4-((phenylsulfonyl)ethynyl)benzene, 1d

Flash chromatography of the crude reaction product [*n*-hexane-EtOAc (5:1)] afforded **1d** as a white solid (67%, 456 mg), with a melting point of 103-105 °C.

^{1}H NMR ($CDCl_3$, 300 MHz): δ 1.31 (s, 9H), 7.38-7.58 (m, 4H), 7.58-7.69 (m, 3H), 8.08-8.12 (m, 2H) ppm.

^{13}C NMR ($CDCl_3$, 75.5 MHz): δ 31.0, 35.2, 84.9, 94.3, 114.7, 125.8, 127.3, 129.4, 132.6, 134.1, 142.0, 155.5, 170.5 ppm.

HRMS (EI) calcd. for $C_{18}H_{22}NO_2S$ [$M+NH_4^+$]: 316.1366, found: 316.1369.

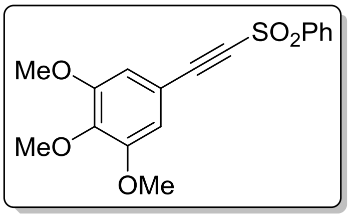

1,2,3-Trimethoxy-5-((phenylsulfonyl)ethynyl)benzene, 1j

Flash chromatography of the crude reaction product [*n*-hexane-EtOAc (5:1)] afforded **1j** as a white solid (61%, 566 mg), with a melting point of 114-116 °C.

[7] Pelter, A.; Ward, R. S.; Little, G. M. *J. Chem. Soc. Perkin Trans. 1*, **1990**, 2775.

^{1}H NMR ($CDCl_3$, 300 MHz): δ 3.76 (s, 6H), 3.79 (s, 3H), 6.69 (s, 2H), 7.51-7.62 (m, 3H), 7.99-8.02 (m, 2H) ppm.

^{13}C NMR ($CDCl_3$, 75.5 MHz): δ 56.3 (CH_3), 61.0 (CH_3), 84.5 (C), 94.0 (C), 110.1 (CH), 112.3 (C), 127.4 (CH), 129.4 (CH), 134.1 (CH), 141.7 (C), 141.8 (C), 153.2 (C) ppm.

HRMS (EI) calcd. for $C_{17}H_{20}NO_5S$ [$M+NH_4^+$]: 350.1057, found: 350.1057.

General procedure for the synthesis of (E)-β-fluorovinyl sulfones.

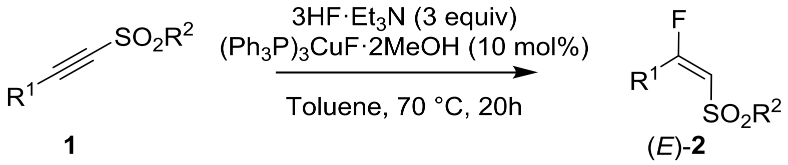

A screw-top eppendorf tube was charged with alkynyl sulfone (1 equiv) and $(Ph_3P)_3CuF·2MeOH$ (10 mol%), and purged with nitrogen. Toluene (0.05 M) was then added, followed by $3HF·Et_3N$ (3 equiv), and the resulting mixture was heated at 70 °C for 20 h in an oil bath. When the reaction was complete, the crude mixture was concentrated and purified by flash column chromatography using mixtures of hexane and ethyl acetate as the eluent (assuming the reaction was complete; if not, mixtures of hexane and DCM (2:1) was used in order to separate the product from the alkynyl sulfone starting material).

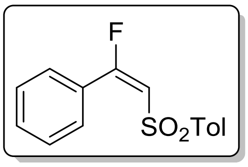

(*E*)-**1-((2-Fluoro-2-phenylvinyl)sulfonyl)-4-methylbenzene,** (*E*)-**2a**

Flash chromatography of the crude reaction product [*n*-hexane:EtOAc (4:1)] afforded (*E*)-**2a** as a colorless oil (35%, 65 mg).

^{1}H NMR ($CDCl_3$, 300 MHz): δ 2.33 (s, 3H), 6.38 (d, *J*= 18.4 Hz, 1H), 7.16-7.19 (m, 2H), 7.36-7.38 (m, 2H), 7.38-7.43 (m, 1H), 7.54-7.59 (m, 4H) ppm.

^{13}C NMR ($CDCl_3$, 75.5 MHz): 21.6 (CH_3), 114.5 (d, *J*= 31.8 Hz, C), 127.4 (CH), 128.1 (d, *J*= 16.3 Hz, C), 128.3 (CH), 129.5 (d, *J*= 5.0 Hz, CH), 129.6 (CH), 129.7 (d, *J*= 2.2 Hz, CH), 132.0 (2C), 138.5 (d, *J*= 2.9 Hz, C), 138.6, 144.5 (C), 167.7 (d, *J*= 276.4 Hz, C) ppm.

^{19}F NMR ($CDCl_3$, 282.4 MHz): δ -71.74 (d, *J*= 18.4 Hz, 1F) ppm.

HRMS (EI) calcd. for $C_{15}H_{14}FO_2S$ [$M+H^+$]: 277.0693, found: 277.0698.

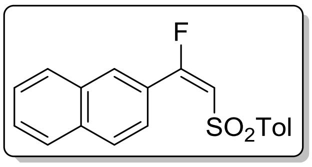

(*E*)-**2-(1-Fluoro-2-tosylvinyl)naphthalene,** (*E*)-**2b**

Flash chromatography of the crude reaction product [*n*-hexane:EtOAc (5:1)] afforded (*E*)-**2b** as a pale yellow solid (38%, 16 mg) with a melting point of 42-43 °C.

^{1}H NMR ($CDCl_3$, 300 MHz): δ 2.29 (s, 3H), 6.47 (d, *J*= 18.4 Hz, 1H), 7.10-7.13 (d, 2H), 7.49-7.57 (m, 5H), 7.77–7.86 (m, 3H), 8.15 (s, 1H) ppm.

^{13}C NMR ($CDCl_3$, 75.5 MHz): δ 21.6, 114.7 (d, *J*= 32.0 Hz), 124.9 (d, *J*= 3.8 Hz), 125.6 (d, *J*= 25.7 Hz), 126.9, 127.3, 127.4, 127.8 (d, *J*= 8.2 Hz), 128.3, 129.1, 129.7, 131.2 (d, *J*= 6.5 Hz), 132.0, 134.7, 138.5, 144.5, 167.7 (d, *J*= 276.6 Hz, CF) ppm.

^{19}F NMR ($CDCl_3$, 282.4 MHz): δ −71.93 (d, *J*= 18.4 Hz, 1F) ppm.

HRMS (EI) calcd. for $C_{19}H_{19}FNO_2S$ [M+NH_4^+]: 344.1115, found: 344.1120.

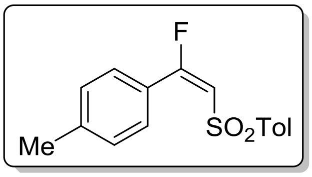

(*E*)-**1-((2-Fluoro-2-(4-tolyl)vinyl)sulfonyl)-4-methylbenzene,** (*E*)-**2c**

Flash chromatography of the crude reaction product [*n*-hexane:EtOAc (4:1)] afforded (*E*)-**2c** as a colourless oil (43%, 18 mg).

^{1}H NMR ($CDCl_3$, 300 MHz): δ 2.44 (s, 6H), 6.40 (d, *J*= 18.5 Hz, 1H), 7.25-7.30 (m, 4H), 7.58-7.60 (d, 2H), 7.67-7.69 (d, 2H) ppm.

^{13}C NMR ($CDCl_3$, 75.5 MHz): δ 21.6 (CH_3), 21.7 (CH_3), 113.7 (d, *J*= 32.6 Hz, CH), 125.6 (d, *J*= 26.0 Hz, C), 127.3 (CH), 128.8 (CH), 129.6 (d, *J*= 5.6 Hz, CH), 129.7 (CH), 138.7 (d, *J*= 2.7 Hz, C), 142.7 (C), 144.4 (C), 167.8 (d, *J*= 275.7 Hz, CF) ppm.

^{19}F NMR ($CDCl_3$, 282.4 MHz): δ −72.04 (d, *J*= 18.6 Hz, 1F) ppm.

HRMS (EI) calcd. for $C_{16}H_{19}FNO_2S$ [M+NH_4^+]: 308.1115, found: 308.1116.

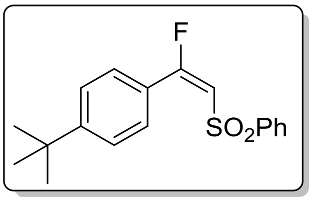

(*E*)-**1-(*tert*-Butyl)-4-(1-fluoro-2-(phenylsulfonyl)vinyl)benzene,** (*E*)-**2d**

Flash chromatography of the crude reaction product [*n*-hexane:EtOAc (4:1)] afforded (*E*)-**2d** as a colourless oil (45%, 19 mg).

^{1}H NMR ($CDCl_3$, 300 MHz): δ 1.36 (s, 9H), 6.45 (d, *J*= 18.4 Hz, 1H), 7.43-7.48 (m, 5H), 7.55-7.62 (m, 2H), 7.76-7.79 (m, 2H) ppm.

^{13}C NMR ($CDCl_3$, 75.5 MHz): δ 31.1 (3xCH_3), 35.1 (C), 113.7 (d, *J*= 32.9 Hz, CH), 125.1 (CH), 125.2 (C), 127.4 (CH), 128.6 (d, *J*= 11.8 Hz, CH), 129.0 (CH), 129.4 (d, *J*= 5.2 Hz, CH), 133.3 (d, *J*= 10.3 Hz, CH), 141.5 (C), 155.8 (C), 168.2 (d, *J*= 276.3 Hz, CF) ppm.

^{19}F NMR ($CDCl_3$, 282.4 MHz): δ −71.61 (d, *J*= 18.4 Hz, 1F) ppm.

HRMS (EI) calcd. for $C_{18}H_{23}FNO_2S$ [M+NH_4^+]: 336.1428, found: 336.1425.

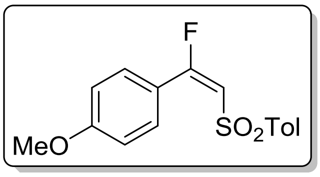

(*E*)-**1-((2-Fluoro-2-(4-methoxyphenyl)vinyl)sulfonyl)-4-methylbenzene,** (*E*)-**2e**

Flash chromatography of the crude reaction product [*n*-hexane:EtOAc (3:1)] afforded (*E*)-**2e** as a colourless oil (51%, 30 mg).

^{1}H NMR ($CDCl_3$, 300 MHz): δ 2.33 (s, 3H), 3.79 (s, 3H), 6.26 (d, *J*= 18.8 Hz, 1H), 6.86 (dd, *J*= 9.1, 0.9 Hz, 2H), 7.17-7.20 (m, 2H), 7.65 (s, 2H), 7.58-7.61 (m, 4H) ppm.

^{13}C NMR ($CDCl_3$, 75.5 MHz): δ 21.6 (CH_3), 55.5 (CH_3), 112.7 (d, *J*= 33.5 Hz, C), 113.5 (CH), 120.6 (d, *J*= 26.7 Hz, C), 127.3 (CH), 128.5 (CH), 128.7, 129.7, 131.6 (d, *J*= 5.8 Hz, C), 133.8 (d, *J*= 19.4 Hz, C), 138.8 (d, *J*= 3.0 Hz, C), 144.4 (C), 162.6 (d, *J*= 1.5 Hz, C), 167.4 (d, *J*= 274.4 Hz, C) ppm.

^{19}F NMR ($CDCl_3$, 282.4 MHz): δ -72.67 (d, *J*= 18.8 Hz, 1F) ppm.

HRMS (EI) calcd. for $C_{16}H_{15}FO_3S$ [M+H^+]: 307.0799, found: 307.0803.

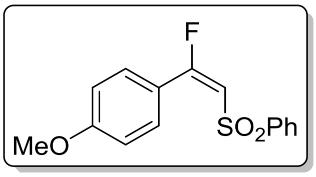

(*E*)-**1-(1-Fluoro-2-(phenylsulfonyl)vinyl)-4-methoxybenzene**, (*E*)-**2f**

Flash chromatography of the crude reaction product [*n*-hexane:EtOAc (3:1)] afforded (*E*)-**2f** as a colourless oil (56%, 40 mg).

^{1}H NMR ($CDCl_3$, 300 MHz): δ 3.86 (s, 3H), 6.35 (d, *J*= 18.6 Hz, 1H), 6.93 (d, *J*= 8.4 Hz, 2H), 7.38-7.50 (m, 2H), 7.51-7.61 (m, 1H), 7.66 (d, *J*= 8.6 Hz, 2H), 7.78 (dd, *J*= 5.3, 3.3 Hz, 2H) ppm.

^{13}C NMR ($CDCl_3$, 75.5 MHz): δ 55.5 (CH_3), 112.4 (d, *J*= 33.6 Hz, CH), 113.6 (CH), 120.5 (d, *J*= 26.6 Hz, C), 127.2 (CH), 129.1 (CH), 131.5 (d, *J*= 5.6 Hz, CH), 133.4 (CH), 141.7 (d, *J*= 2.5 Hz, C), 162.7 (C), 167.8 (d, *J*= 275.1 Hz, C) ppm.

^{19}F NMR ($CDCl_3$, 282.4 MHz): δ -71.77 (d, *J*= 18.6 Hz, 1F) ppm.

HRMS (EI) calcd. for $C_{15}H_{17}FNO_3S$ [$M+NH_4^+$]: 310.0908, found: 310.0911.

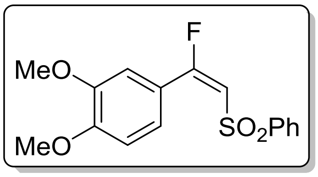

(*E*)-**4-(1-Fluoro-2-(phenylsulfonyl)vinyl)-1,2-dimethoxybenzene,** (*E*)-**2h**

Flash chromatography of the crude reaction product [*n*-hexane:EtOAc (2:1)] afforded (*E*)-**2h** as a white solid (60%, 24 mg) with a melting point of 51-53 °C.

^{1}H NMR ($CDCl_3$, 300 MHz): δ 3.91 (s, 3H), 3.95 (s, 3H), 6.39 (d, *J*= 18.8 Hz, 1H), 6.92 (d, *J*=8.4 Hz, 1H), 7.27 (d, *J*=2.1 Hz, 1H), 7.34 (ddd, *J*= 8.4, 2.0, 0.6 Hz, 1H), 7.45-7.50 (m, 2H), 7.56-7.58 (m, 1H), 7.78-7.81 (m, 2H) ppm.

^{13}C NMR ($CDCl_3$, 75.5 MHz): δ 56.0 ($2OCH_3$), 110.3 (CH), 112.2 (d, *J*= 5.4 Hz, CH), 112.7 (d, *J*= 33.7 Hz, CH), 120.5 (d, *J*= 26.8 Hz, C), 123.7 (d, *J*= 6.5 Hz, CH), 127.2 (CH), 129.1 (CH), 133.4 (CH), 141.6 (d, *J*= 2.5 Hz, C), 148.4 (C), 152.4 (C), 167.5 (d, *J*= 275.0 Hz, C) ppm.

^{19}F NMR ($CDCl_3$, 282.4 MHz): δ -72.68 (d, *J*= 18.9 Hz, 1F) ppm.

HRMS (EI) calcd. for $C_{16}H_{19}FNO_4S$ [$M+NH_4^+$]: 340.1013, found: 340.1019.

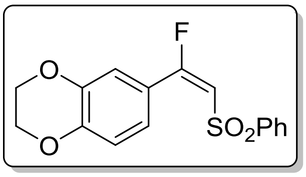

(*E*)-**6-(1-Fluoro-2-(phenylsulfonyl)vinyl)-2,3-dihydrobenzo[b][1,4]dioxine,** (*E*)-**2i**

Flash chromatography of the crude reaction product [*n*-hexane:EtOAc (2:1)] afforded (*E*)-**2i** as a colourless oil (53%, 19 mg).

^{1}H NMR (CDCl$_3$, 300 MHz): δ 4.22 (dqd, *J*= 7.0, 3.3, 1.5 Hz, 4H), 6.28 (d, *J*= 18.6 Hz, 1H), 6.82 (dd, *J*= 8.4, 0.8 Hz, 1H), 7.12-7.14 (m, 2H), 7.40-7.43 (m, 2H), 7.49-7.51 (m, 1H), 7.72-7.75 (m, 2H) ppm.

^{13}C NMR (CDCl$_3$, 75.5 MHz): δ 64.1 (CH$_2$), 64.6 (CH$_2$), 112.8 (d, *J*= 33.5 Hz, CH), 117.1 (CH), 118.8 (d, *J*= 5.5 Hz, CH), 121.2 (d, *J*= 26.6 Hz, C), 123.6 (d, *J*= 5.9 Hz, CH), 127.4 (CH), 129.1 (CH), 133.4 (CH), 141.6 (C), 143.0 (C), 147.1 (C), 167.3 (d, *J*= 275.8 Hz, CF) ppm.

^{19}F NMR (CDCl$_3$, 282.4 MHz): δ -71.81 (d, *J*= 18.6 Hz, 1F) ppm.

HRMS (EI) calcd. for $C_{16}H_{17}FNO_4S$ [M+NH_4^+]: 338.0857, found: 338.0866.

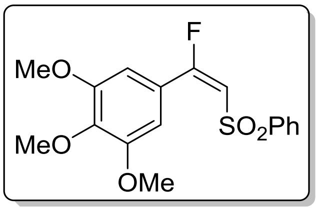

(*E*)-**5-(1-Fluoro-2-(phenylsulfonyl)vinyl)-1,2,3-trimethoxybenzene,** (*E*)-**2j**

Flash chromatography of the crude reaction product [*n*-hexane:EtOAc (2:1)] afforded (*E*)-**2j** as a colourless oil (58%, 23 mg).

^{1}H NMR (CDCl$_3$, 300 MHz): δ 3.88 (s, 6H), 3.93 (s, 3H), 6.46 (d, *J*= 18.6 Hz, 1H), 6.96 (s, 2H), 7.45-7.50 (m, 2H), 7.56-7.59 (m, 1H), 7.76-7.80 (m, 2H) ppm.

^{13}C NMR (CDCl$_3$, 75.5 MHz): δ 56.3 (CH$_3$), 61.0 (CH$_3$), 107.1 (d, *J*= 5.6 Hz, CH), 113.9 (d, *J*= 32.9 Hz, CH), 123.0 (d, *J*= 26.8 Hz, C), 127.2 (CH), 128.6 (d, *J*= 8.2 Hz, CH), 129.0 (CH), 133.4 (CH), 133.8 (d, *J*= 13.9 Hz, CH), 141.4 (C), 152.7 (C), 167.4 (d, *J*= 276.5 Hz, C) ppm.

^{19}F NMR (CDCl$_3$, 282.4 MHz): δ -73.03 (d, *J*= 18.6 Hz, 1F) ppm.

HRMS (EI) calcd. for $C_{17}H_{21}FNO_5S$ [M+NH_4^+]: 370.1119, found: 370.1123.

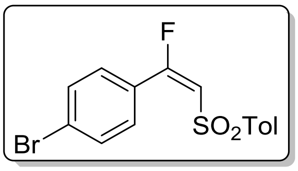

(*E*)-**1-Bromo-4-(1-fluoro-2-tosylvinyl)benzene**, (*E*)-**2m**

Flash chromatography of the crude reaction product [*n*-hexane:EtOAc (5:1)] afforded (*E*)-**2m** as a colourless oil (27%, 13mg).

^{1}H NMR ($CDCl_3$, 300 MHz): δ 2.42 (s, 3H), 6.45 (d, *J*= 18.5 Hz, 1H), 7.26-7.30 (m, 2H), 7.52-7.66 (m, 6H) ppm.

^{13}C NMR ($CDCl_3$, 75.5 MHz): δ 21.6 (CH_3), 114.9 (d, *J*= 31.3 Hz, CH), 127.0 (C), 127.1 (C), 127.4 (CH), 129.8 (CH), 131.0 (d, *J*= 4.9 Hz, CH), 131.4 (CH), 138.3 (C), 144.8 (C), 166.27 (d, *J*= 275.7 Hz, C) ppm.

^{19}F NMR ($CDCl_3$, 282.4 MHz): δ -73.51 (d, *J*= 18.5 Hz, 1F) ppm.

HRMS (EI) calcd. for $C_{15}H_{16}BrFNO_2S$ [$M+NH_4^+$]: 372.0064, found: 372.0068.

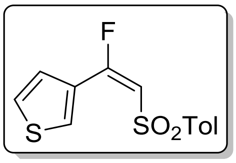

(*E*)-**3-(1-Fluoro-2-tosylvinyl)thiophene,** (*E*)-**2n**

Flash chromatography of the crude reaction product [*n*-hexane:EtOAc (4:1)] afforded (*E*)-**2n** as a colourless oil (63%, 23 mg).

^{1}H NMR ($CDCl_3$, 300 MHz): δ 2.34 (s, 3H), 6.28 (d, *J*= 20.4 Hz, 1H), 7.22-7.25 (m, 3H), 7.43-7.45 (m, 1H), 7.63-7.65 (m, 2H), 8.12 (ddd, *J*= 3.0, 1.3, 0.7 Hz, 1H) ppm.

^{13}C NMR ($CDCl_3$, 75.5 MHz): δ 21.6 (CH_3), 112.7 (d, *J*= 33.1 Hz, C), 125.9 (CH), 127.2 (s, CH), 127.5 (d, *J*= 5.0 Hz, CH), 129.8 (CH), 132.3 (d, *J*= 8.3 Hz, C), 138.7 (d, *J*= 2.8 Hz, C), 144.6 (C), 162.4 (d, *J*= 269.0 Hz, C) ppm.

^{19}F NMR ($CDCl_3$, 282.4 MHz): δ -77.29 (dd, *J*= 20.5, 1.3 Hz, 1F) ppm.

HRMS (EI) calcd. for $C_{15}H_{13}FO_2S$ [$M+H^+$]: 283.0257, found: 283.0257.

…………………………………………………………………………………

Identification of E/Z isomers of fluorovinyl sulfone products

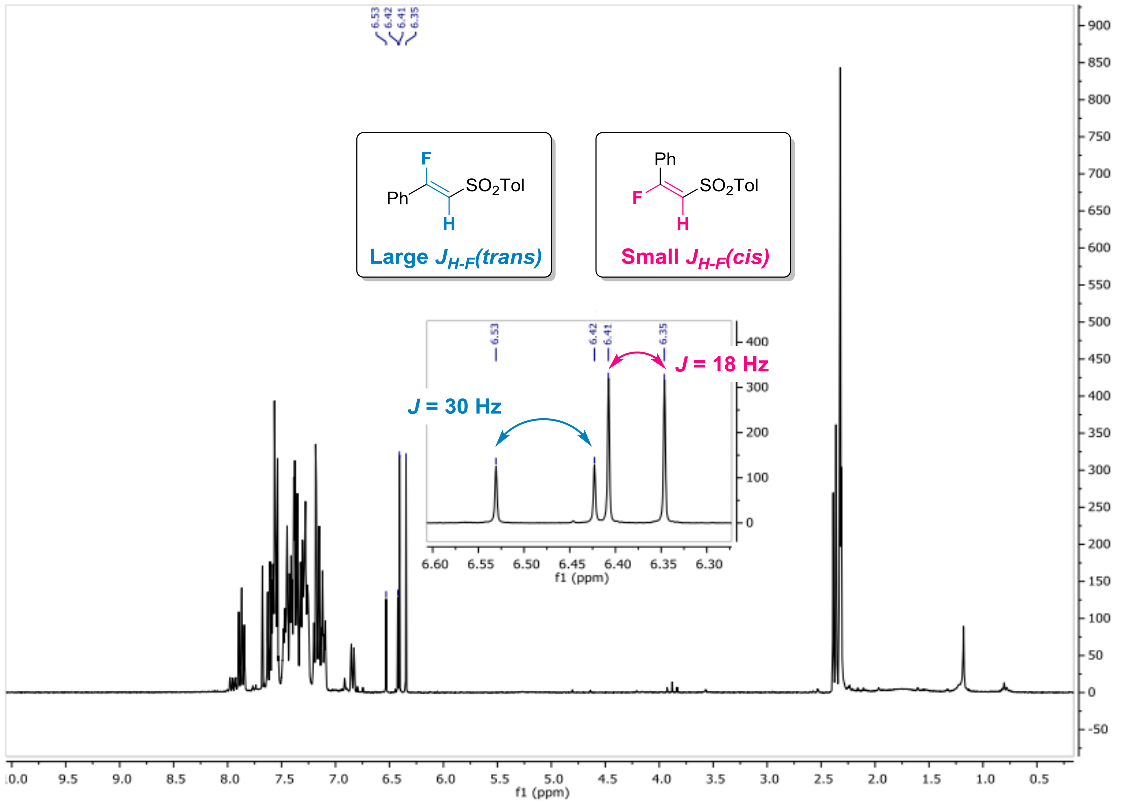

Crude reaction mixture for the preparation of compound **2a**. The two isomers are clearly detected through the signal belonging to the olefin proton in the ^{1}H NMR spectrum and can be identified by their respective coupling constants with the adjacent fluorine atom.

…………………………………………………………………………………………

^{1}H NMR of control experiment with CD_3OD

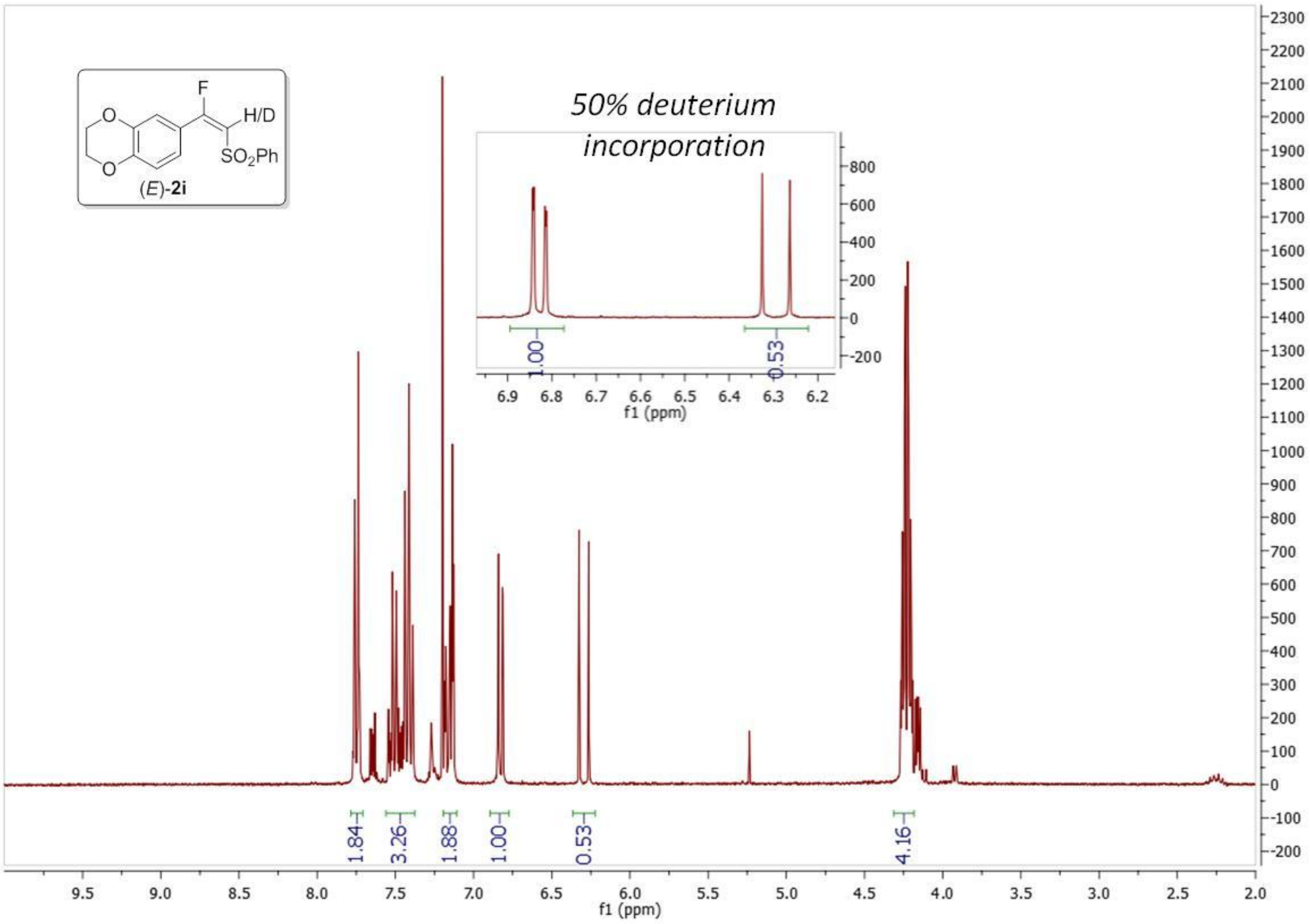

....

NMR spectra of new compounds

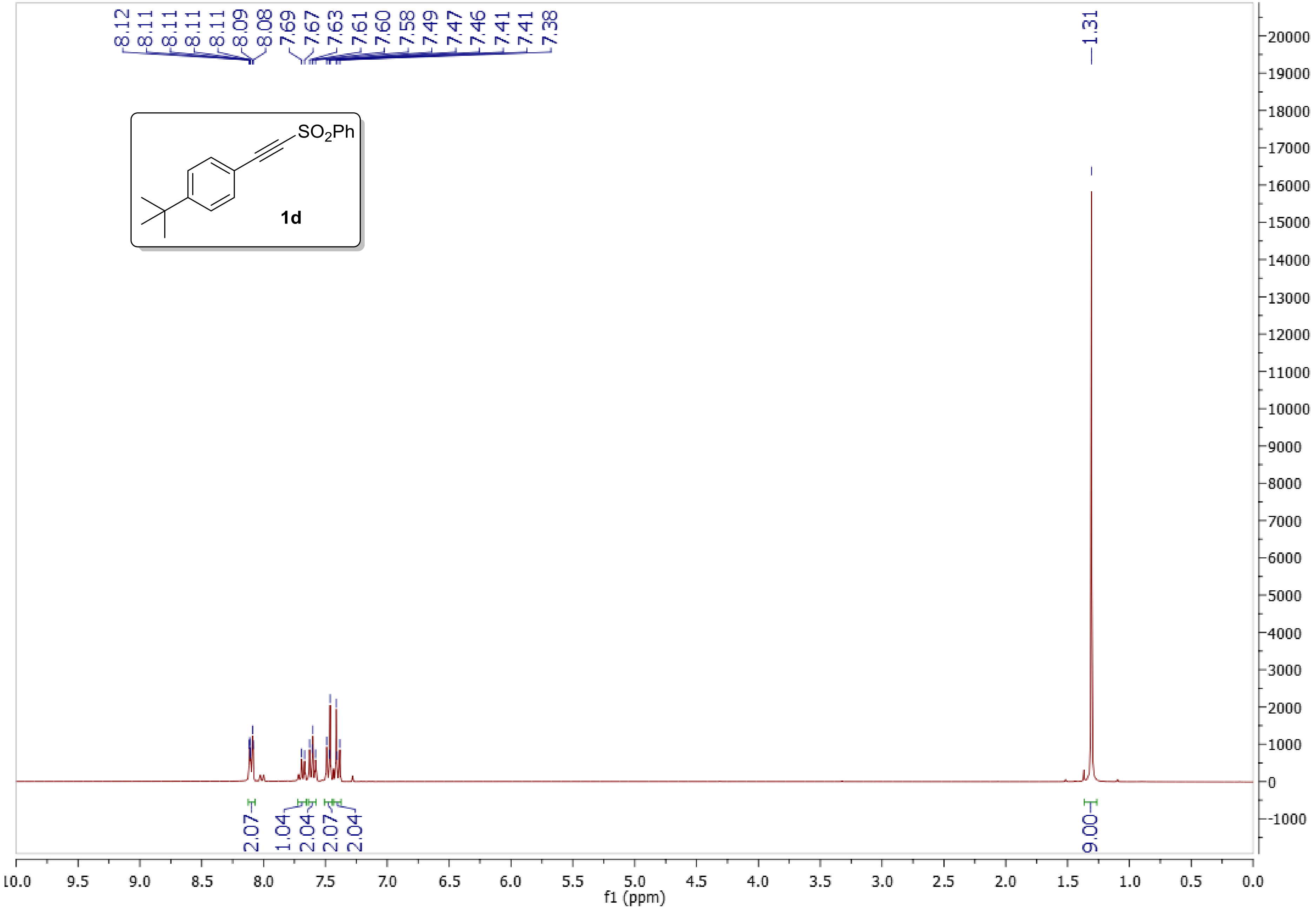
8.12 8.11 8.11 8.11 8.11 8.09 8.08 7.69 7.67 7.63 7.61 7.60 7.58 7.49 7.47 7.46 7.41 7.41 7.38
1.31
SO2Ph
1d
2.07
1.04
2.04
2.07
2.04
9.00
f1 (ppm)

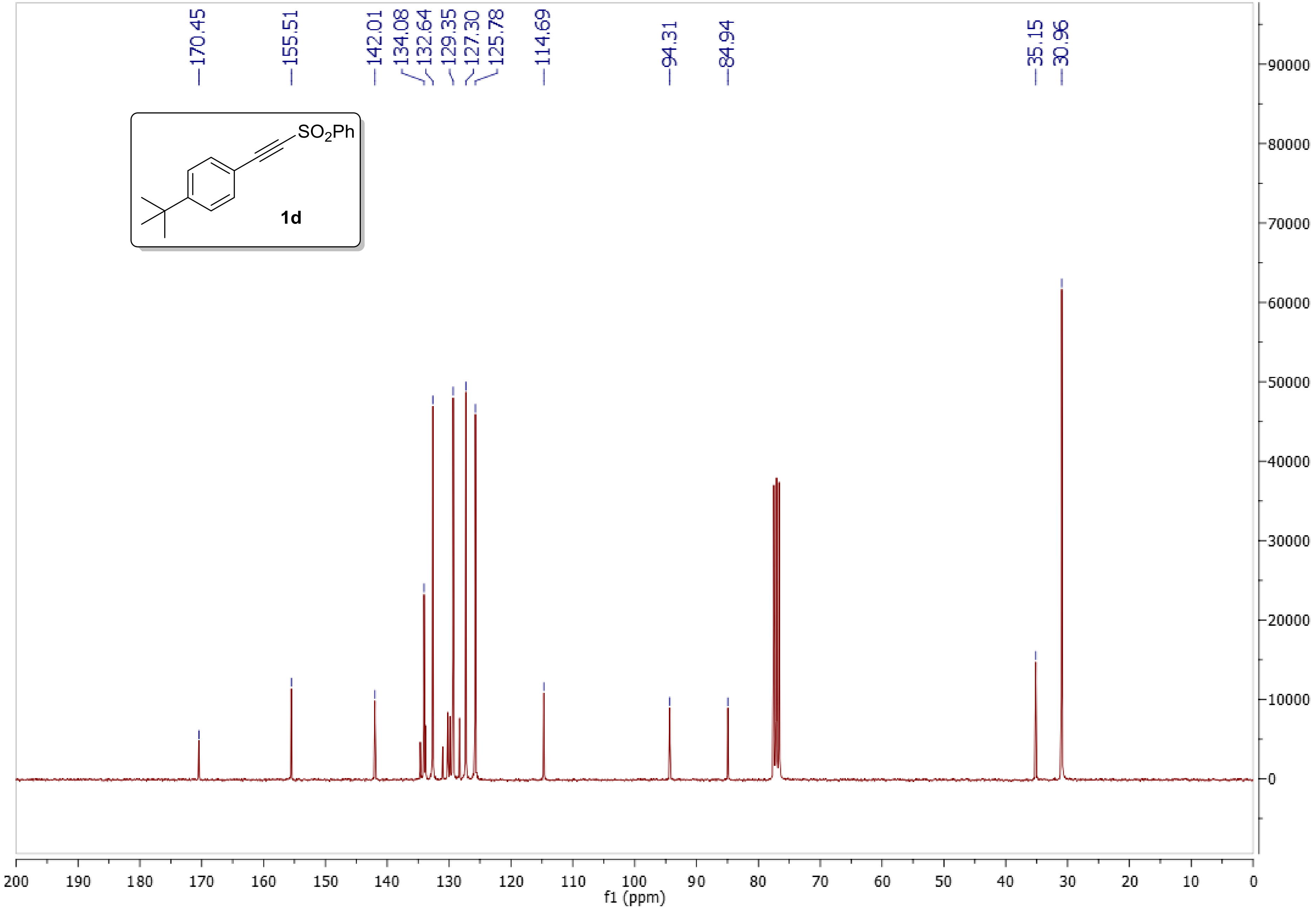
170.45
155.51
142.01
134.08
132.64
129.35
127.30
125.78
114.69
94.31
84.94
35.15
30.96
SO2Ph
1d
f1 (ppm)
200 190 180 170 160 150 140 130 120 110 100 90 80 70 60 50 40 30 20 10 0
90000 80000 70000 60000 50000 40000 30000 20000 10000 0

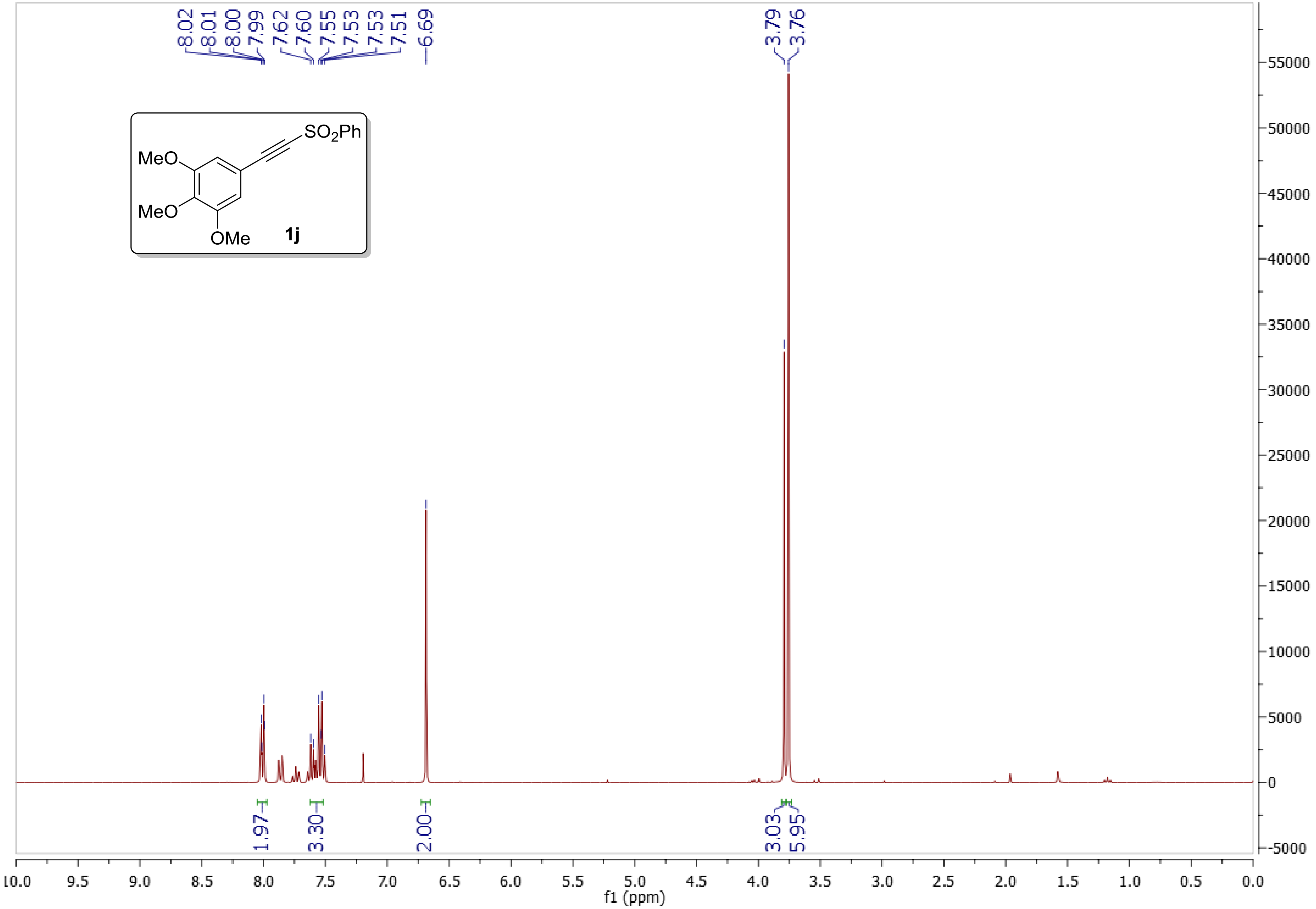

8.02
8.01
8.00
7.99
7.62
7.60
7.55
7.53
7.53
7.51
6.69
3.79
3.76
MeO
MeO
OMe
SO2Ph
1j
1.97
3.30
2.00
3.03
5.95
f1 (ppm)

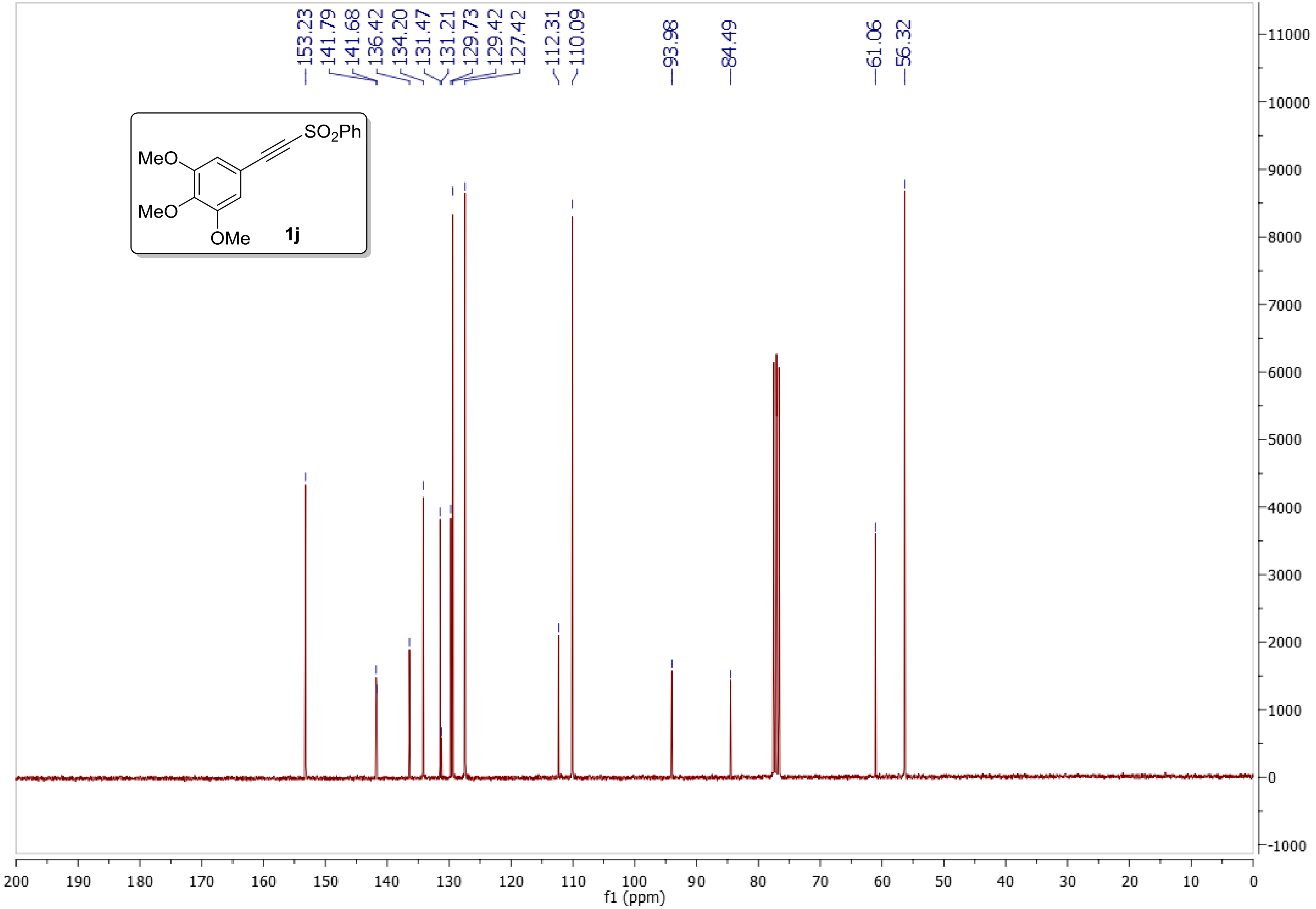

153.23
141.79
141.68
136.42
134.20
131.47
131.21
129.73
129.42
127.42
112.31
110.09
93.98
84.49
61.06
56.32
MeO
MeO
OMe
SO2Ph
1j
f1 (ppm)

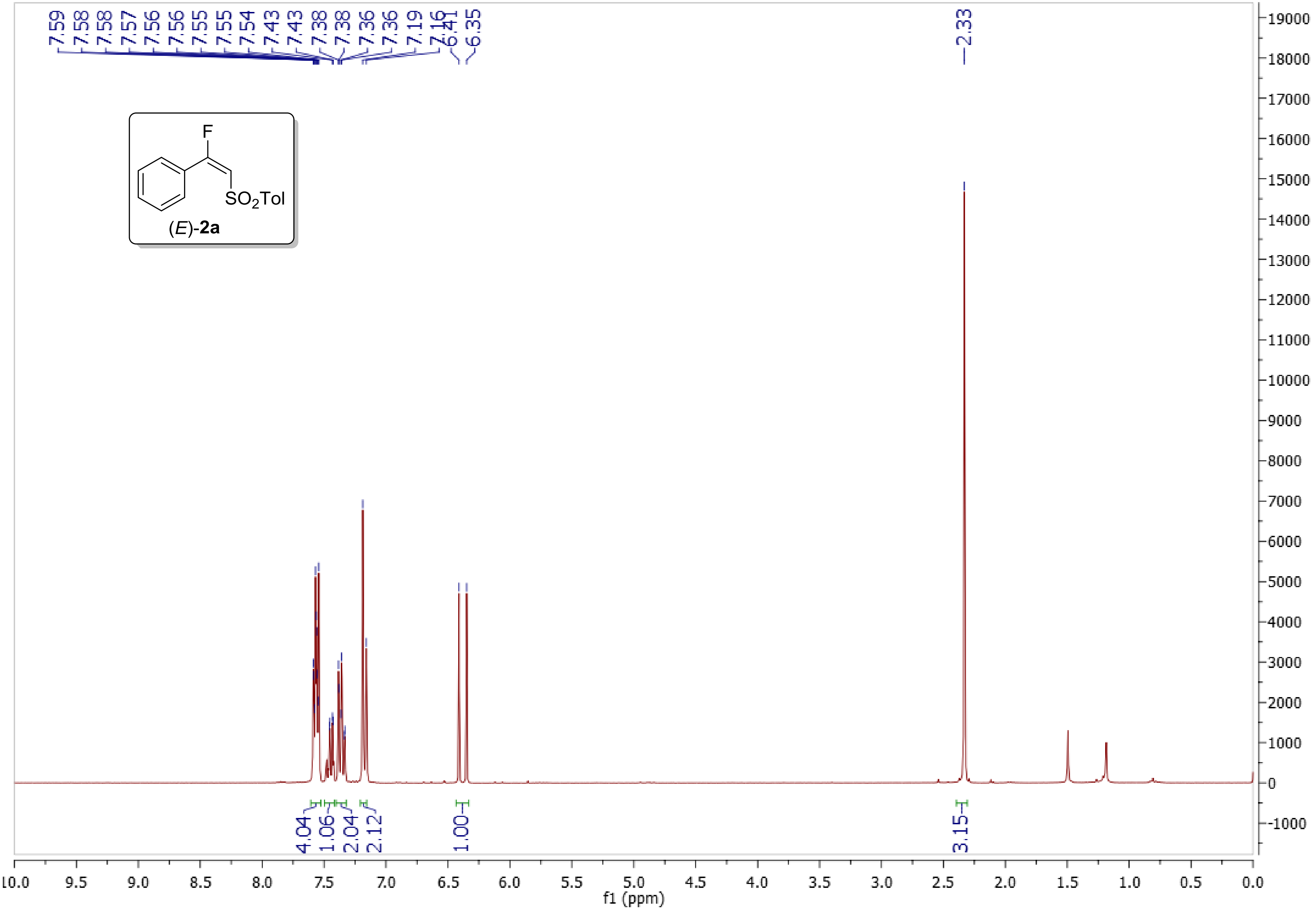
7.59 7.58 7.58 7.57 7.56 7.56 7.55 7.55 7.54 7.43 7.43 7.38 7.38 7.36 7.36 7.19 7.16 6.41 6.35
2.33
F
SO2Tol
(E)-2a
4.04 1.06 2.04 2.12 1.00 3.15
f1 (ppm)

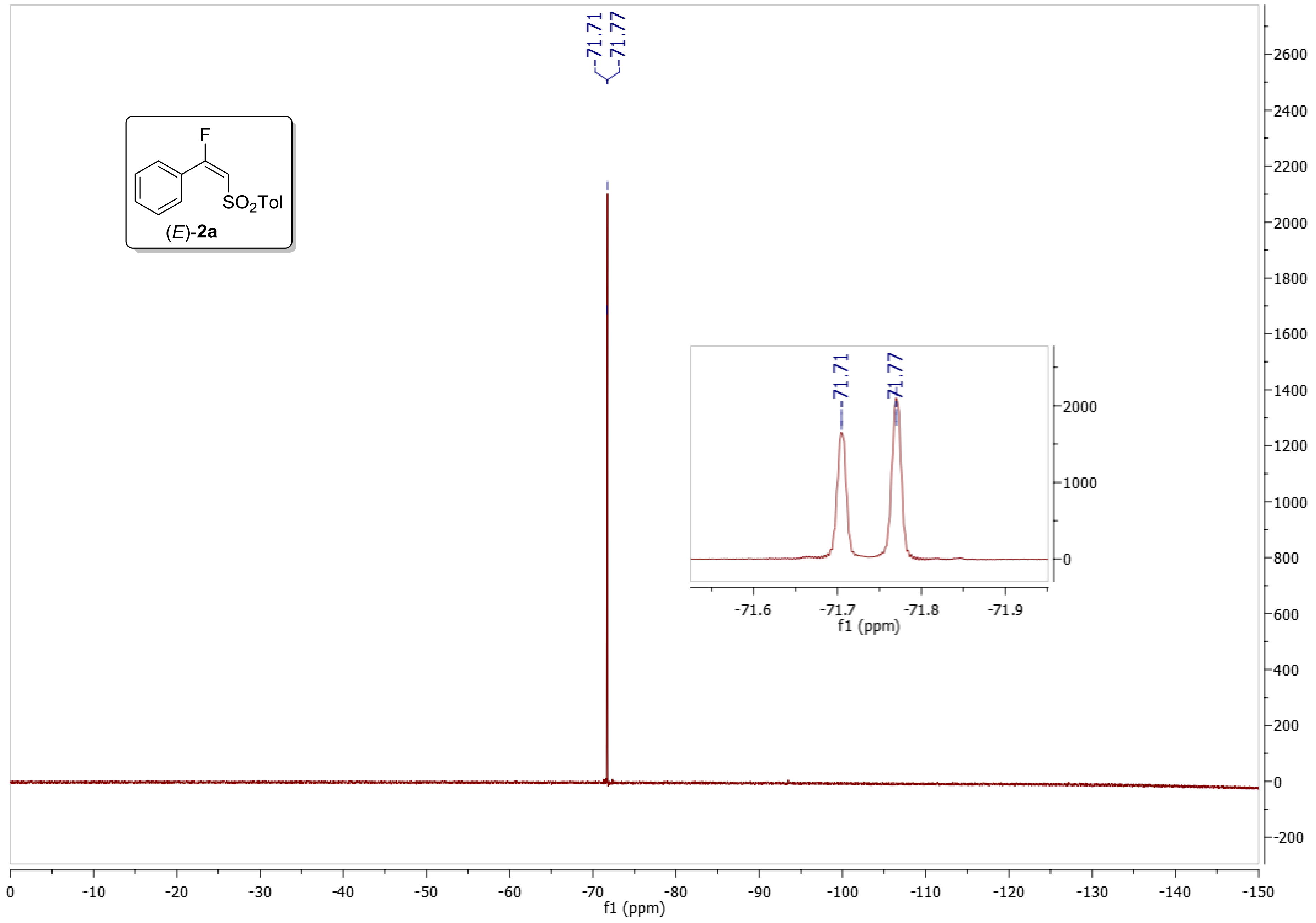
-71.71
-71.77
F
SO2Tol
(E)-2a
-71.6
-71.7
-71.8
-71.9
f1 (ppm)
2000
1000
0
2600
2400
2200
2000
1800
1600
1400
1200
1000
800
600
400
200
0
-200
0
-10
-20
-30
-40
-50
-60
-70
-80
-90
-100
-110
-120
-130
-140
-150
f1 (ppm)

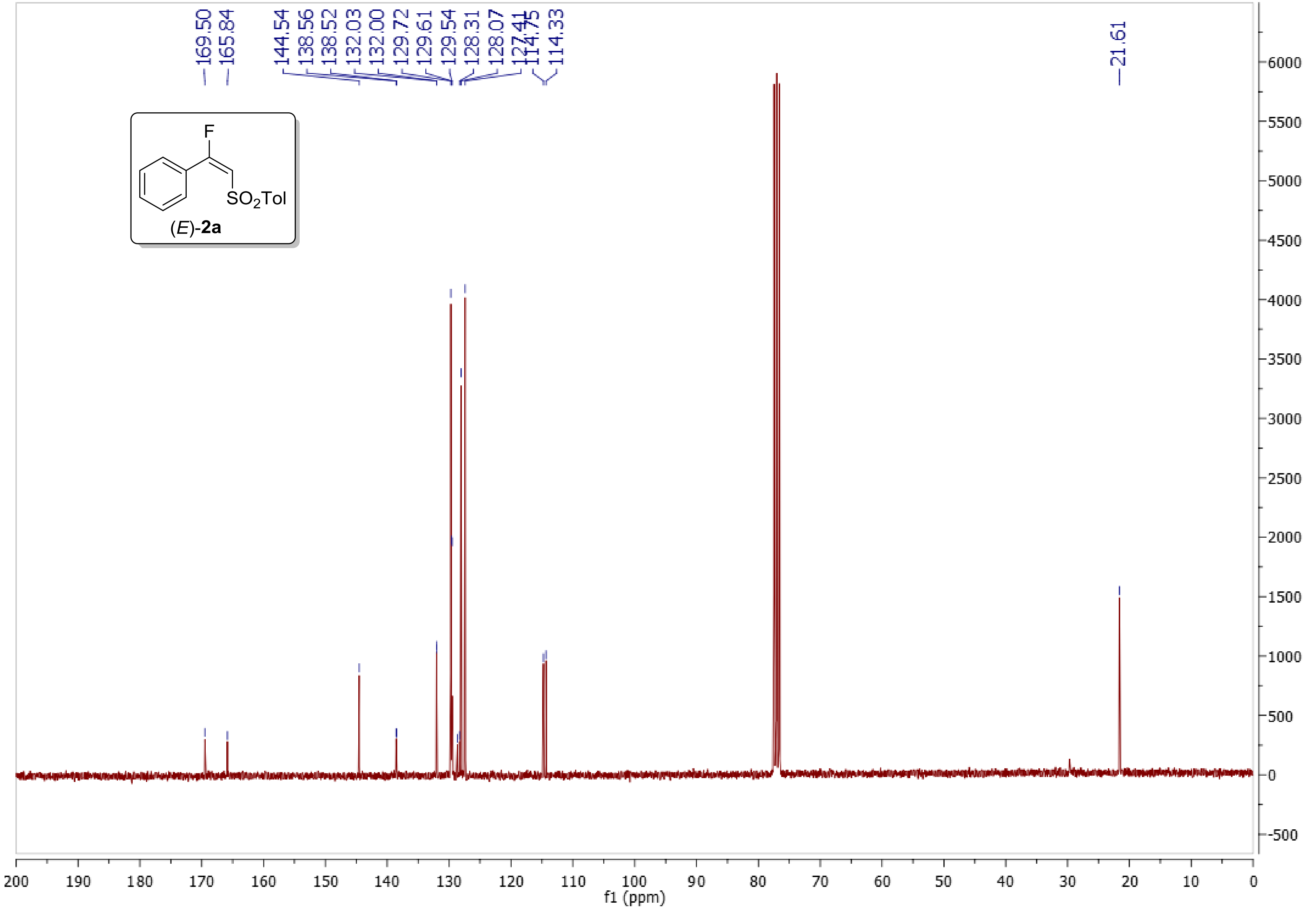

169.50
165.84
144.54
138.56
138.52
132.03
132.00
129.72
129.61
129.54
128.31
128.07
127.41
114.75
114.33
21.61
F
SO2Tol
(E)-2a
f1 (ppm)

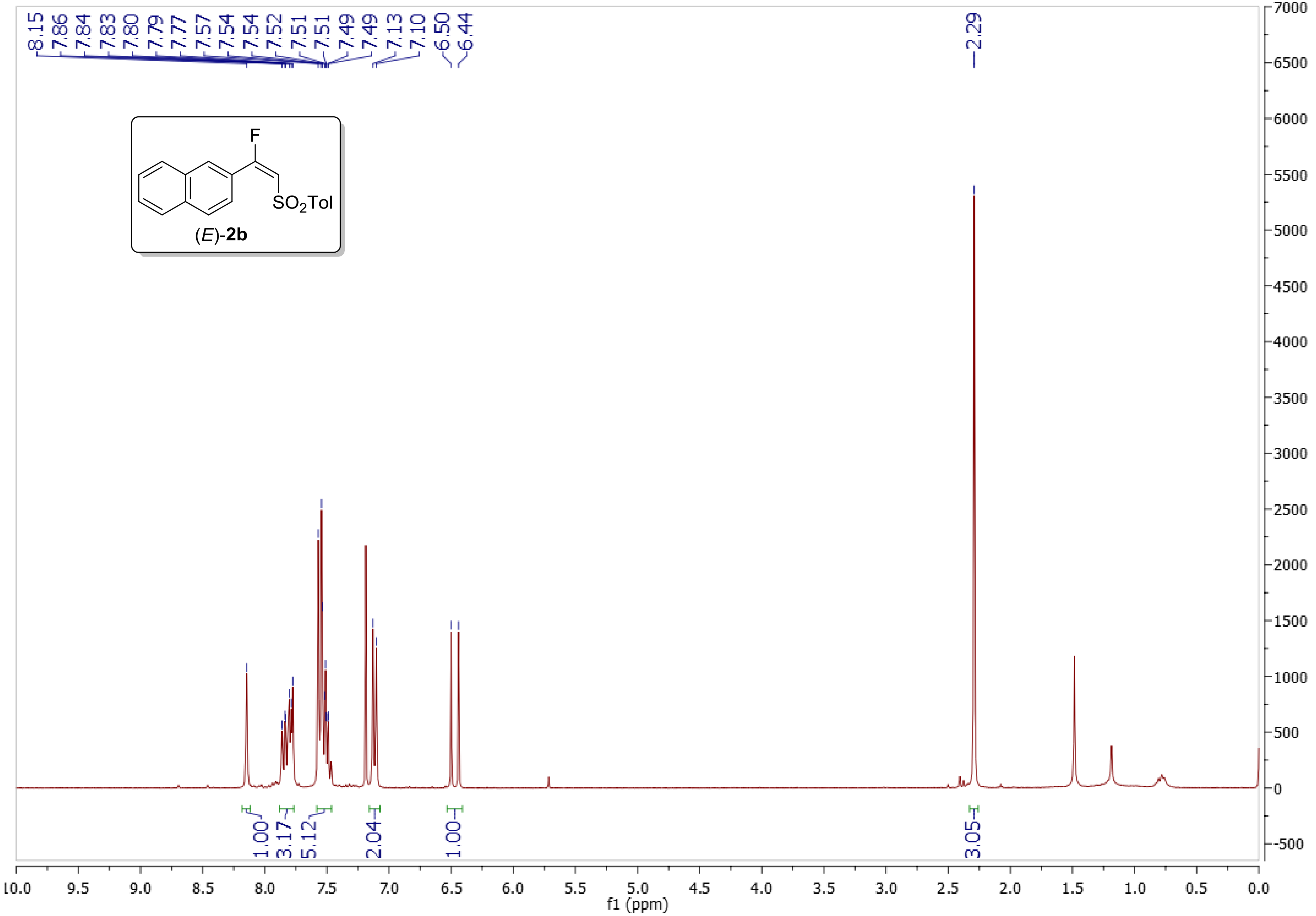

8.15
7.86
7.84
7.83
7.80
7.79
7.77
7.57
7.54
7.54
7.52
7.51
7.51
7.49
7.49
7.13
7.10
6.50
6.44
2.29
F
SO2Tol
(E)-2b
1.00
3.17
5.12
2.04
1.00
3.05
f1 (ppm)

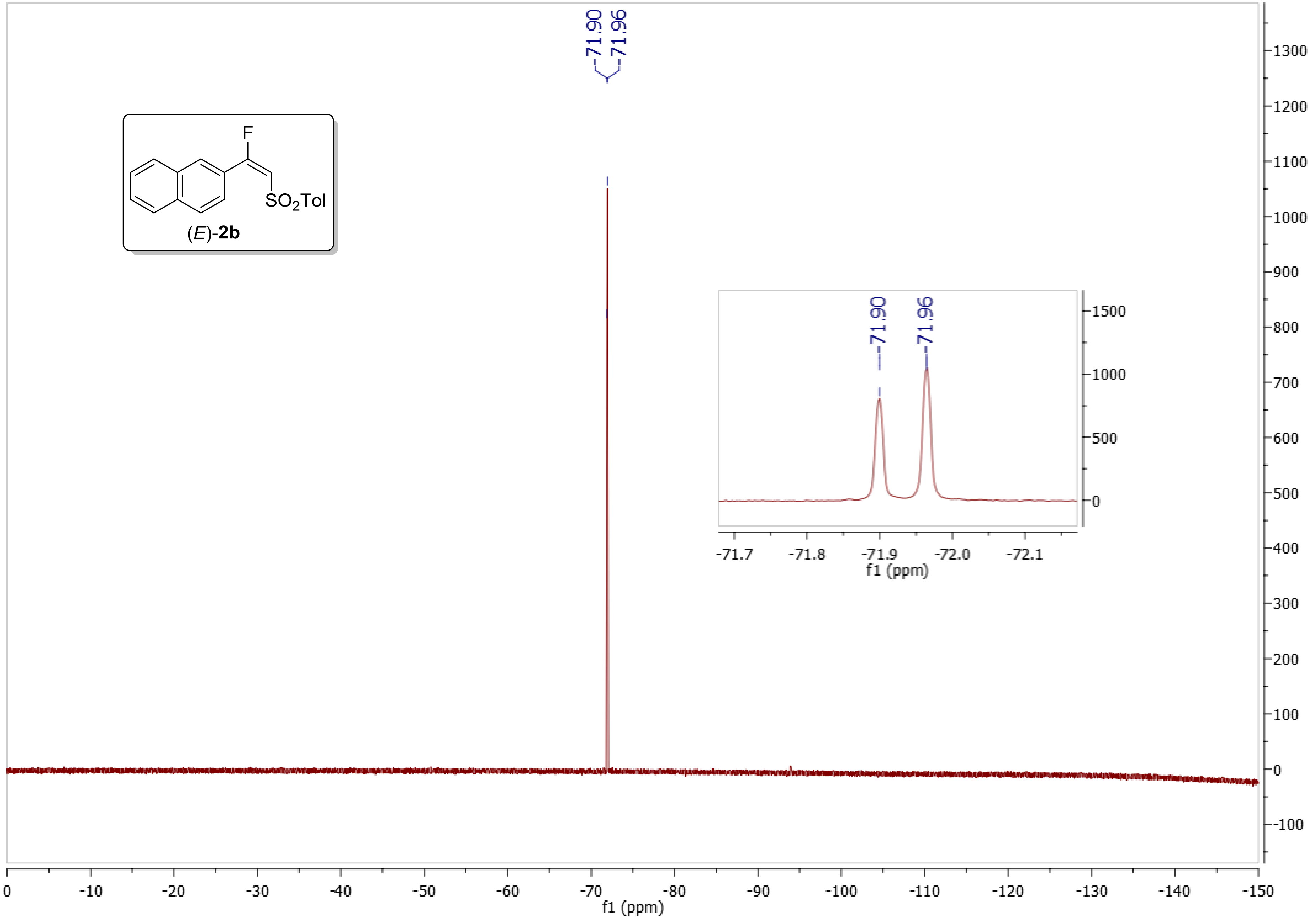

-71.90
-71.96
F
SO2Tol
(E)-2b
f1 (ppm)

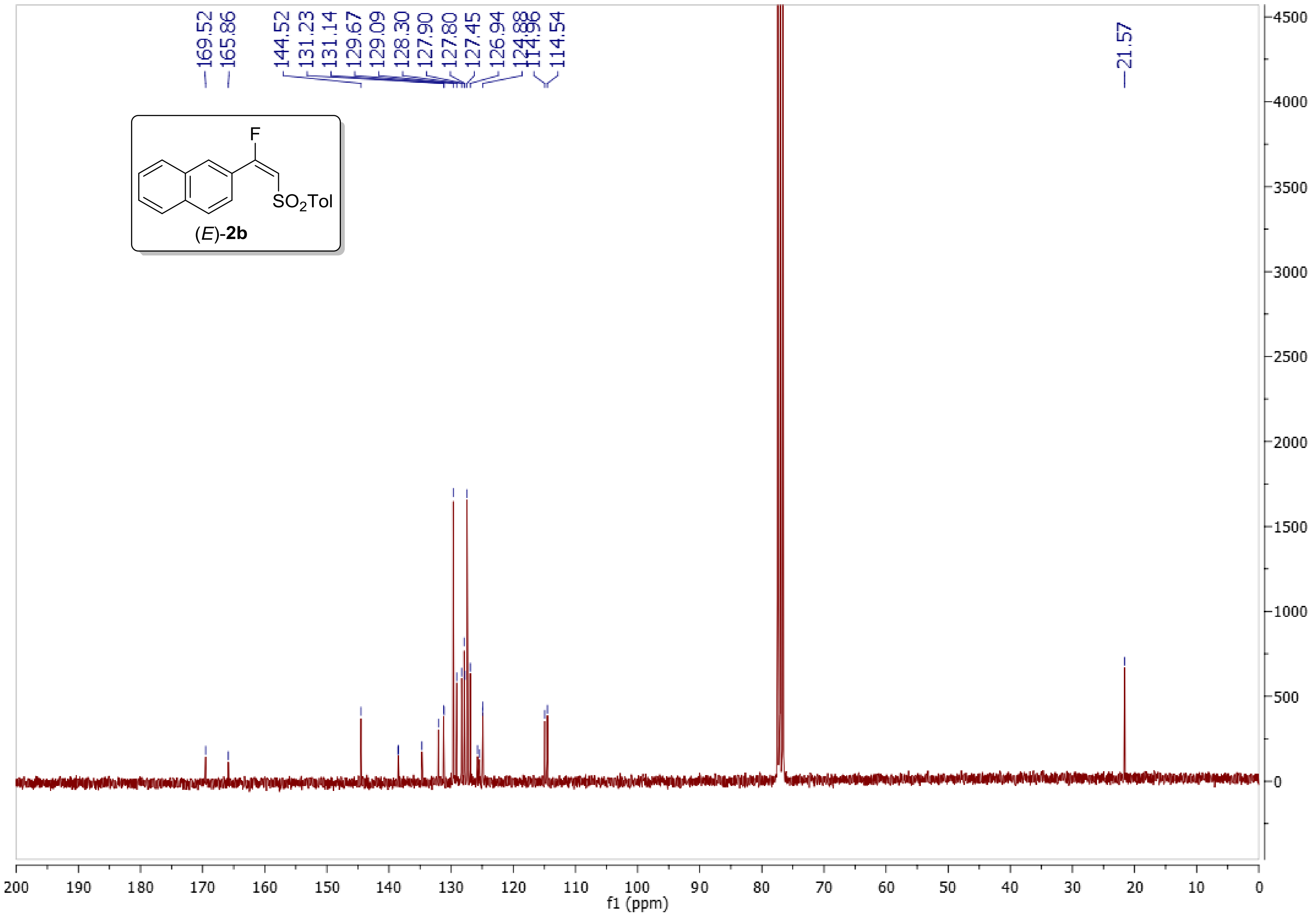
169.52
165.86
144.52
131.23
131.14
129.67
129.09
128.30
127.90
127.80
127.45
126.94
124.88
114.96
114.54
21.57
F
SO2Tol
(E)-2b
f1 (ppm)

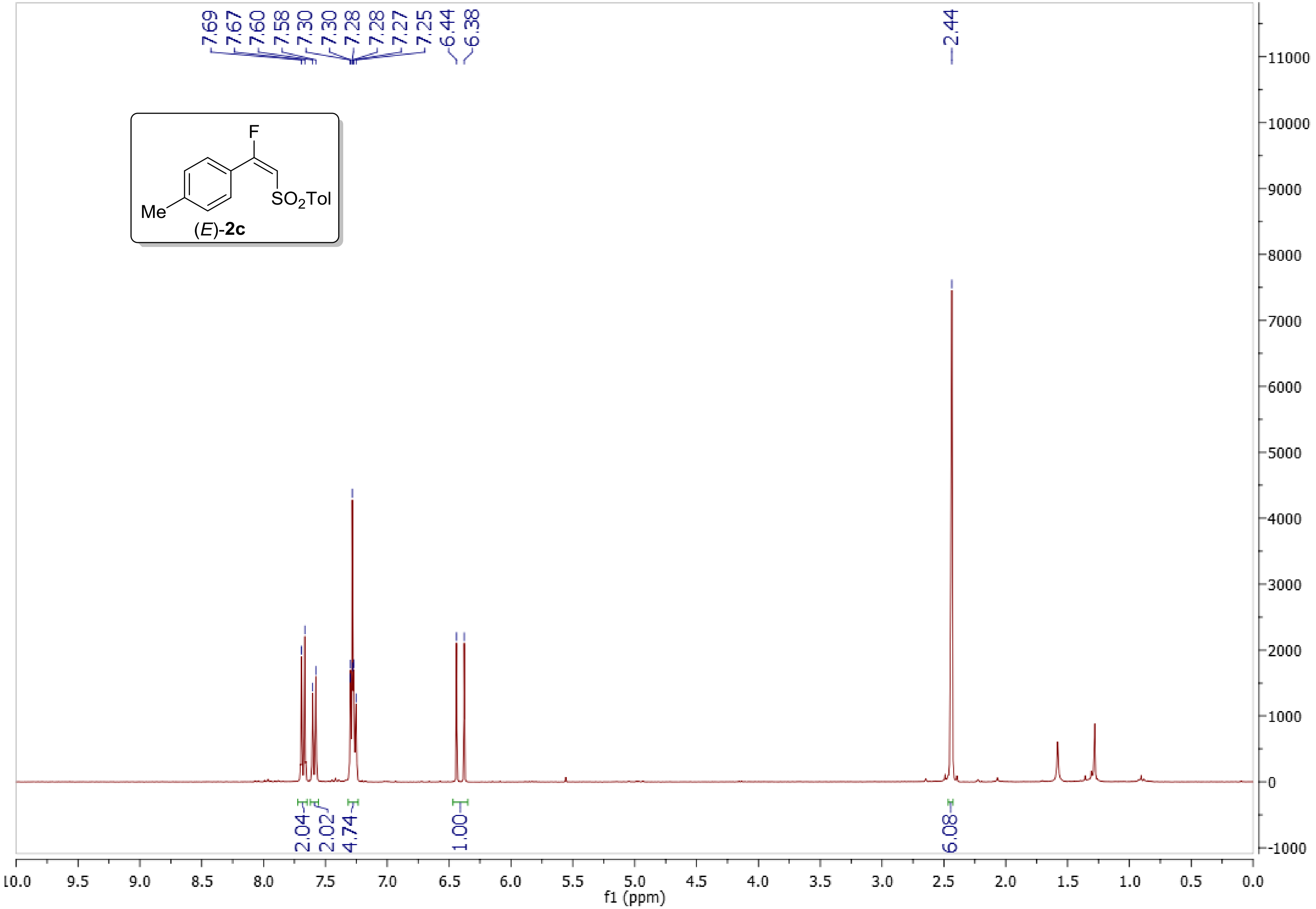
7.69
7.67
7.60
7.58
7.30
7.30
7.28
7.28
7.27
7.25
6.44
6.38
2.44
F
SO2Tol
Me
(E)-2c
2.04
2.02
4.74
1.00
6.08
f1 (ppm)

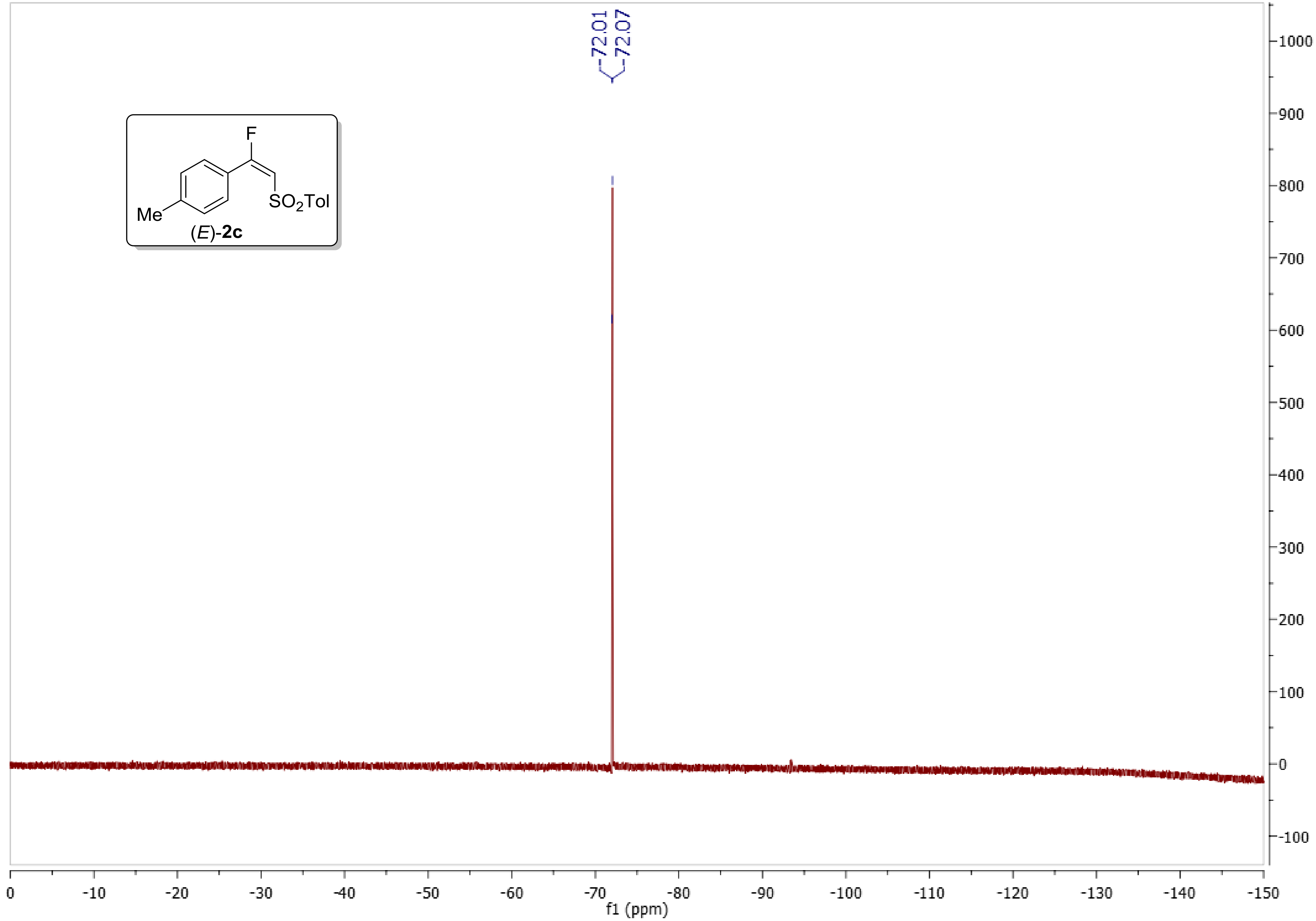

-72.01
-72.07
F
Me
SO_2Tol
(E)-2c
f1 (ppm)

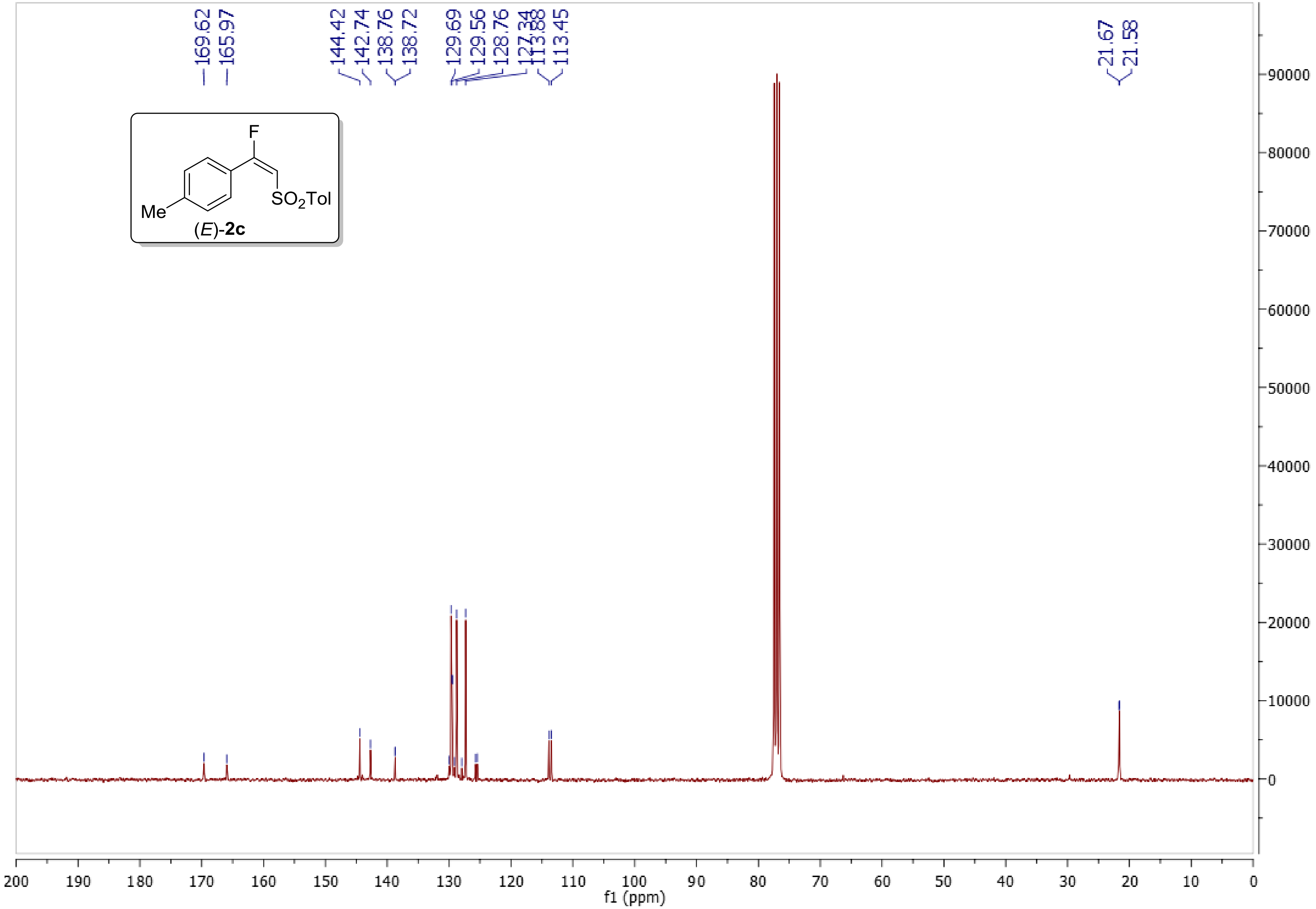
169.62
165.97
144.42
142.74
138.76
138.72
129.69
129.56
128.76
127.34
113.88
113.45
21.67
21.58
F
Me
SO2Tol
(E)-2c
f1 (ppm)

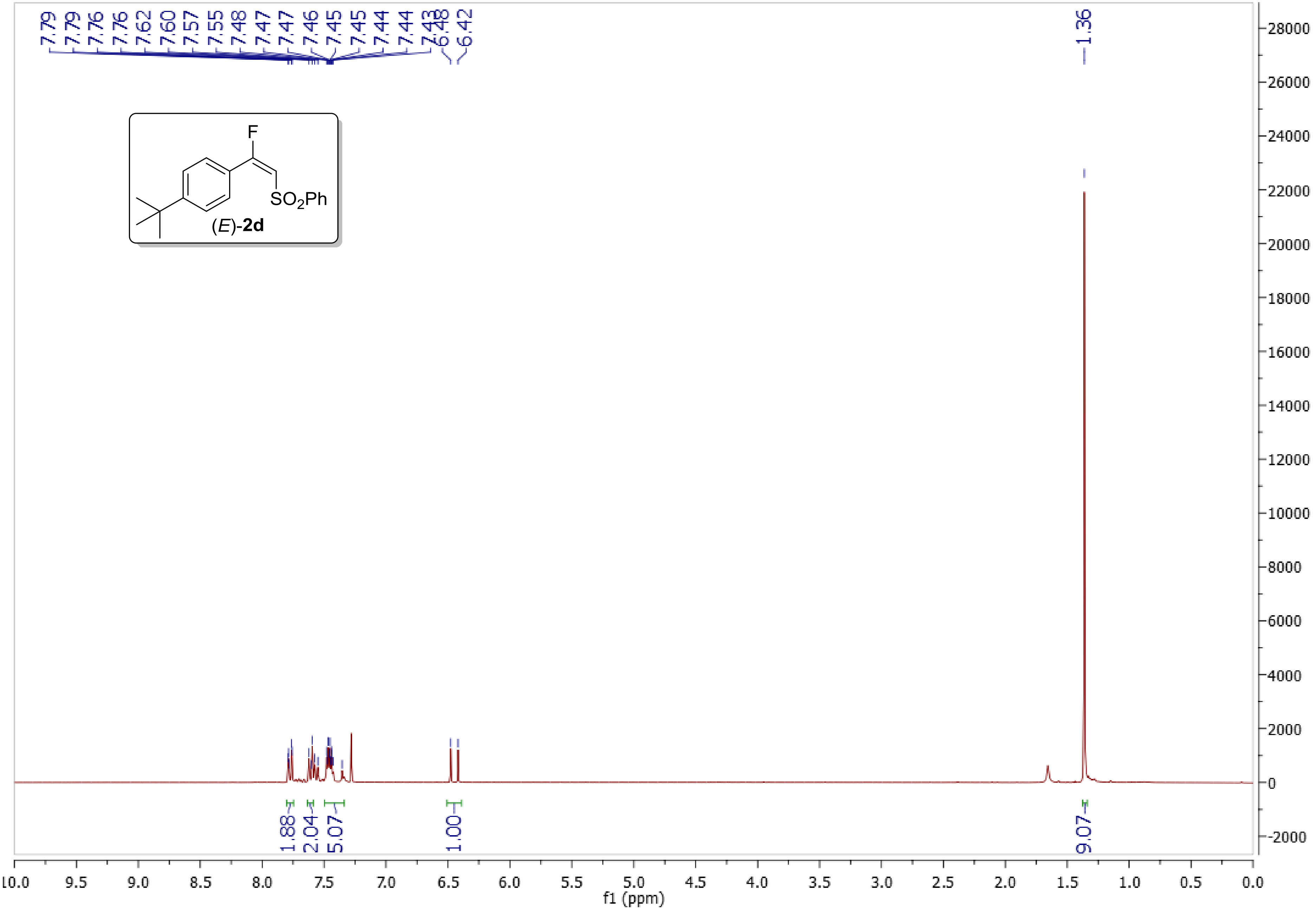

7.79
7.79
7.76
7.76
7.62
7.60
7.57
7.55
7.48
7.47
7.47
7.46
7.45
7.45
7.44
7.44
7.43
6.48
6.42
1.36
F
SO2Ph
(E)-2d
1.88
2.04
5.07
1.00
9.07
f1 (ppm)

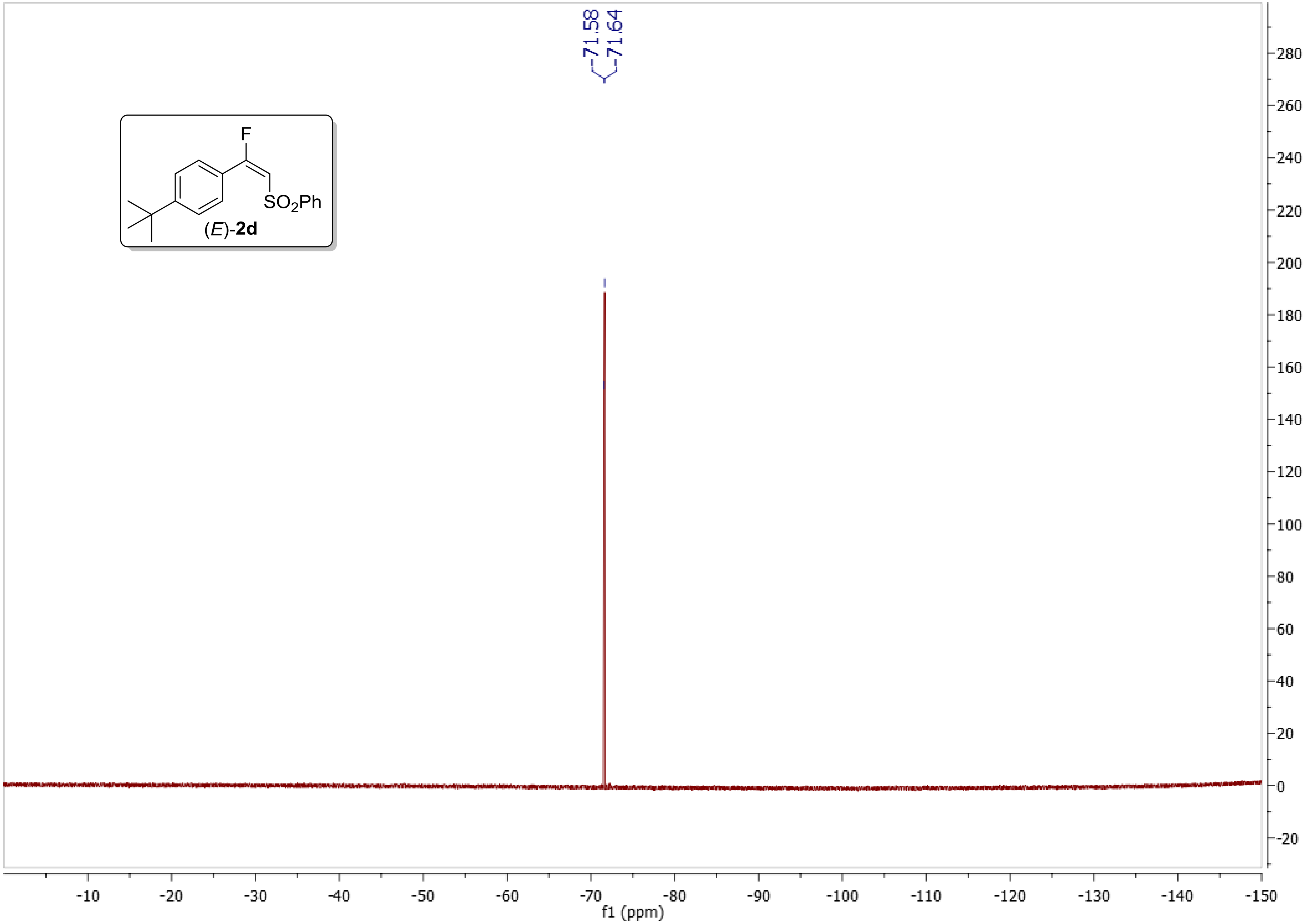
-71.58
-71.64
F
SO2Ph
(E)-2d
f1 (ppm)

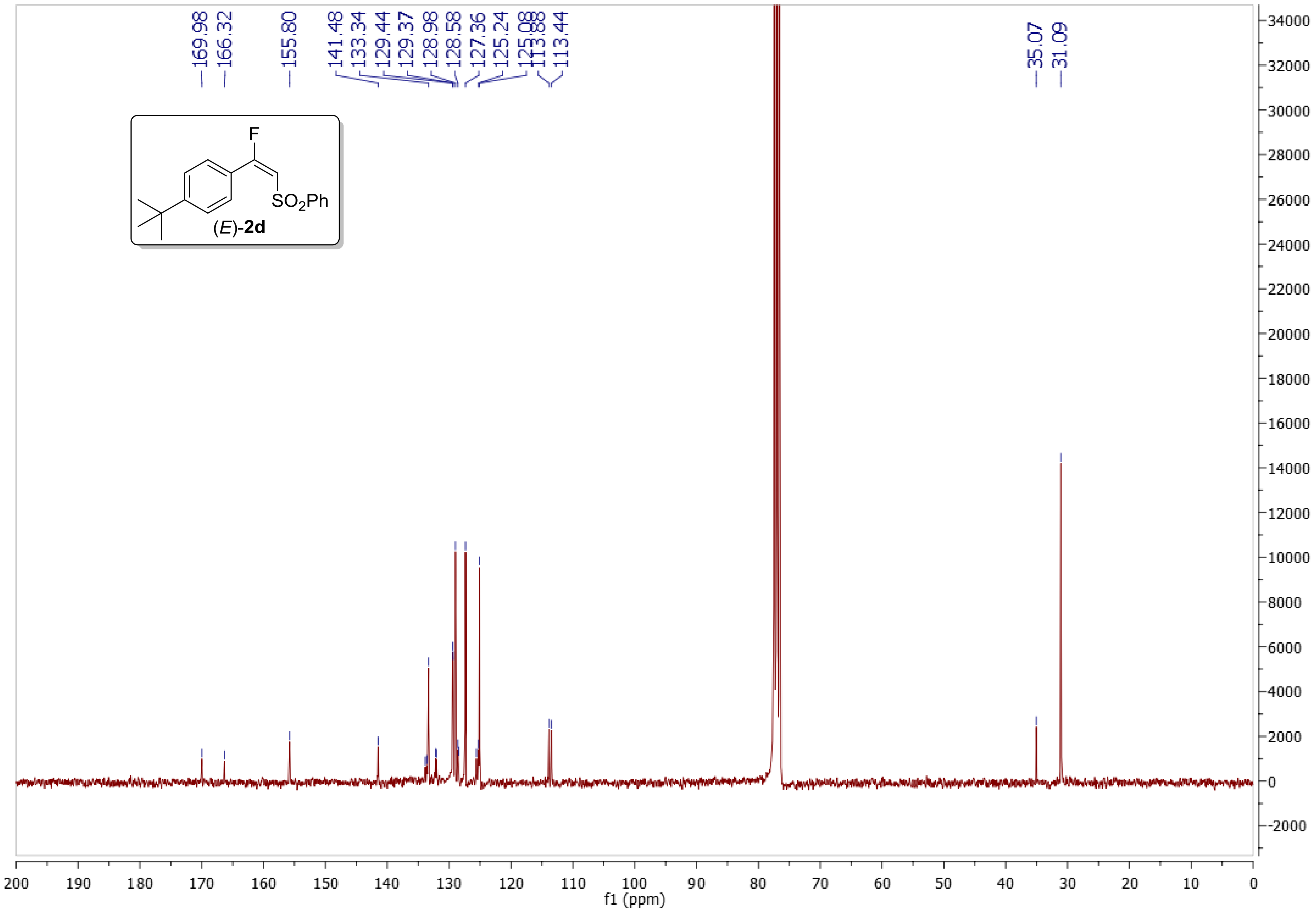

169.98
166.32
155.80
141.48
133.34
129.44
129.37
128.98
128.58
127.36
125.24
125.08
113.88
113.44
35.07
31.09
F
SO2Ph
(E)-2d
200
190
180
170
160
150
140
130
120
110
100
90
80
70
60
50
40
30
20
10
0
f1 (ppm)
34000
32000
30000
28000
26000
24000
22000
20000
18000
16000
14000
12000
10000
8000
6000
4000
2000
0
-2000

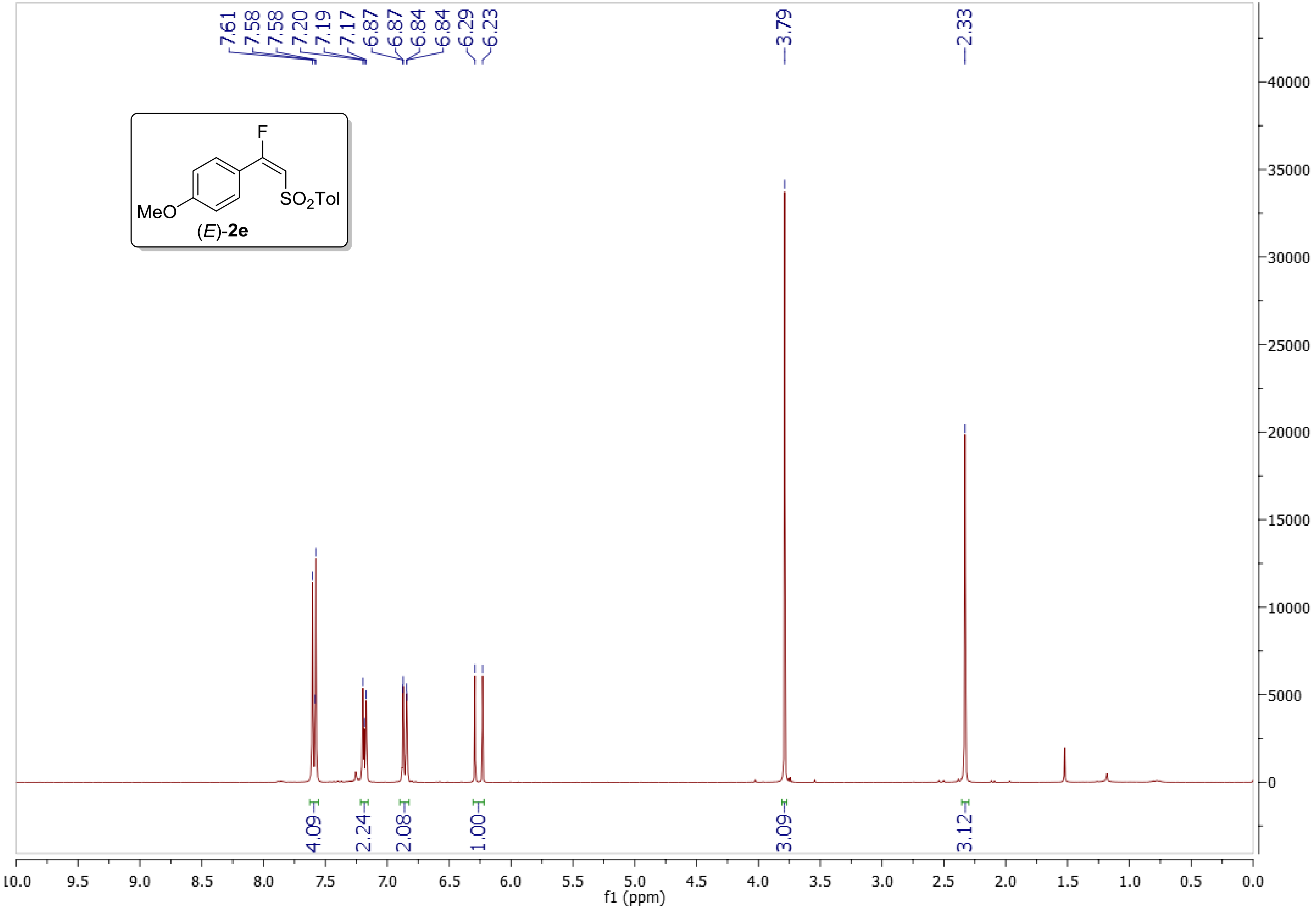

7.61
7.58
7.58
7.20
7.19
7.17
6.87
6.87
6.84
6.84
6.29
6.23
3.79
2.33
F
MeO
SO2Tol
(E)-2e
4.09
2.24
2.08
1.00
3.09
3.12
40000
35000
30000
25000
20000
15000
10000
5000
0
10.0
9.5
9.0
8.5
8.0
7.5
7.0
6.5
6.0
5.5
5.0
4.5
4.0
3.5
3.0
2.5
2.0
1.5
1.0
0.5
0.0
f1 (ppm)

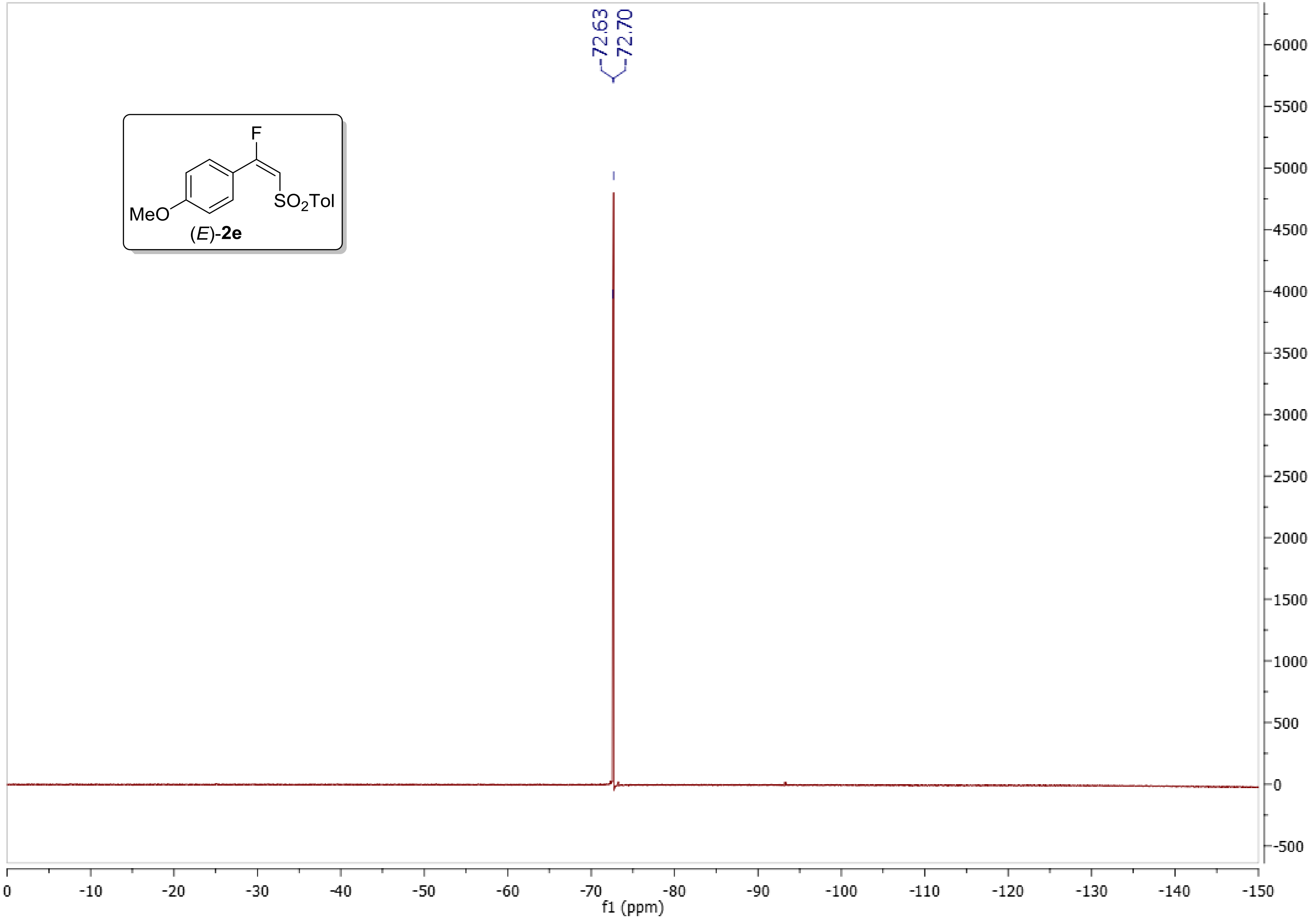
-72.63
-72.70
F
MeO
SO2Tol
(E)-2e
6000
5500
5000
4500
4000
3500
3000
2500
2000
1500
1000
500
0
-500
0
-10
-20
-30
-40
-50
-60
-70
-80
-90
-100
-110
-120
-130
-140
-150
f1 (ppm)

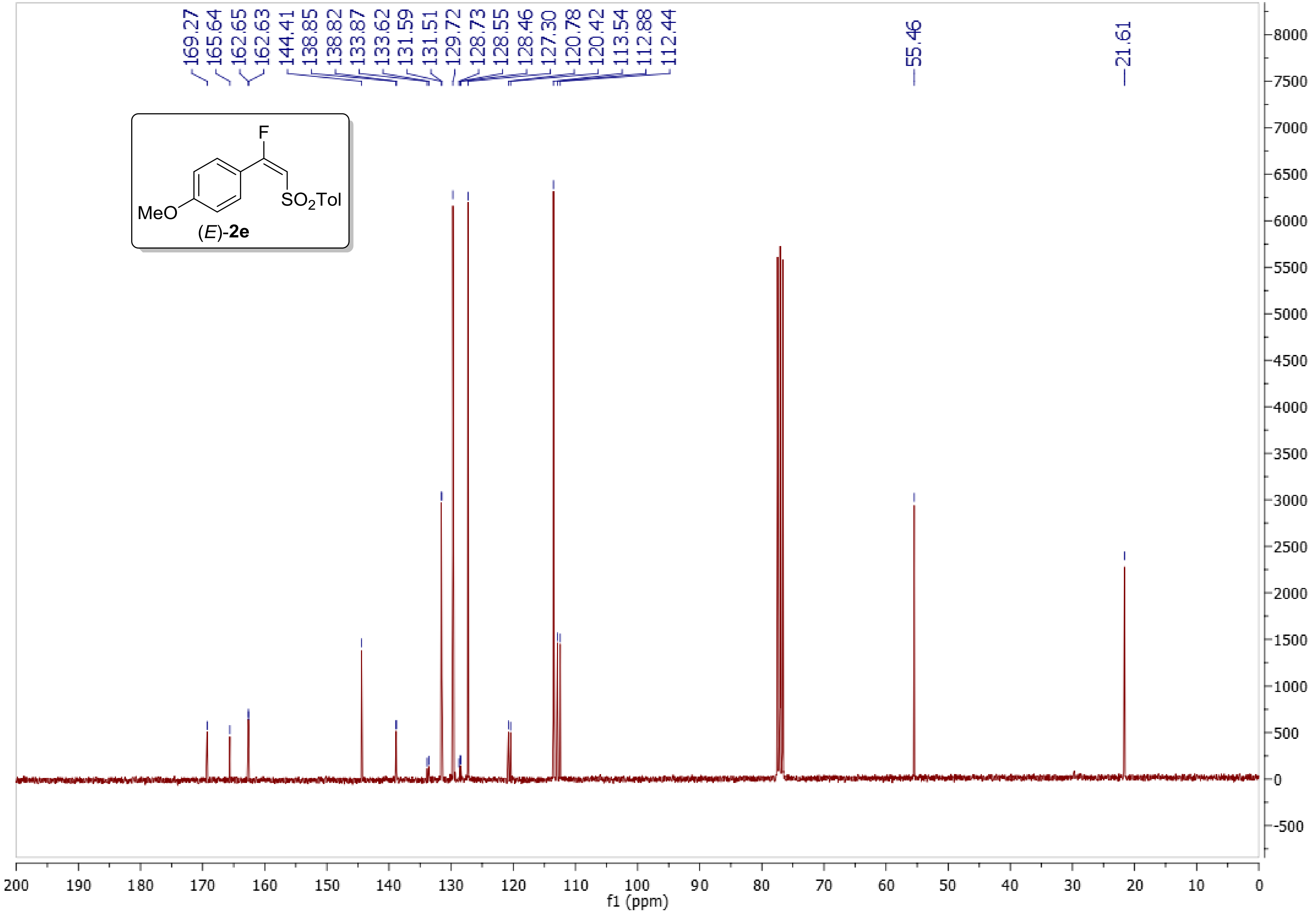
169.27
165.64
162.65
162.63
144.41
138.85
138.82
133.87
133.62
131.59
131.51
129.72
128.73
128.55
128.46
127.30
120.78
120.42
113.54
112.88
112.44
55.46
21.61
F
MeO
SO2Tol
(E)-2e
f1 (ppm)

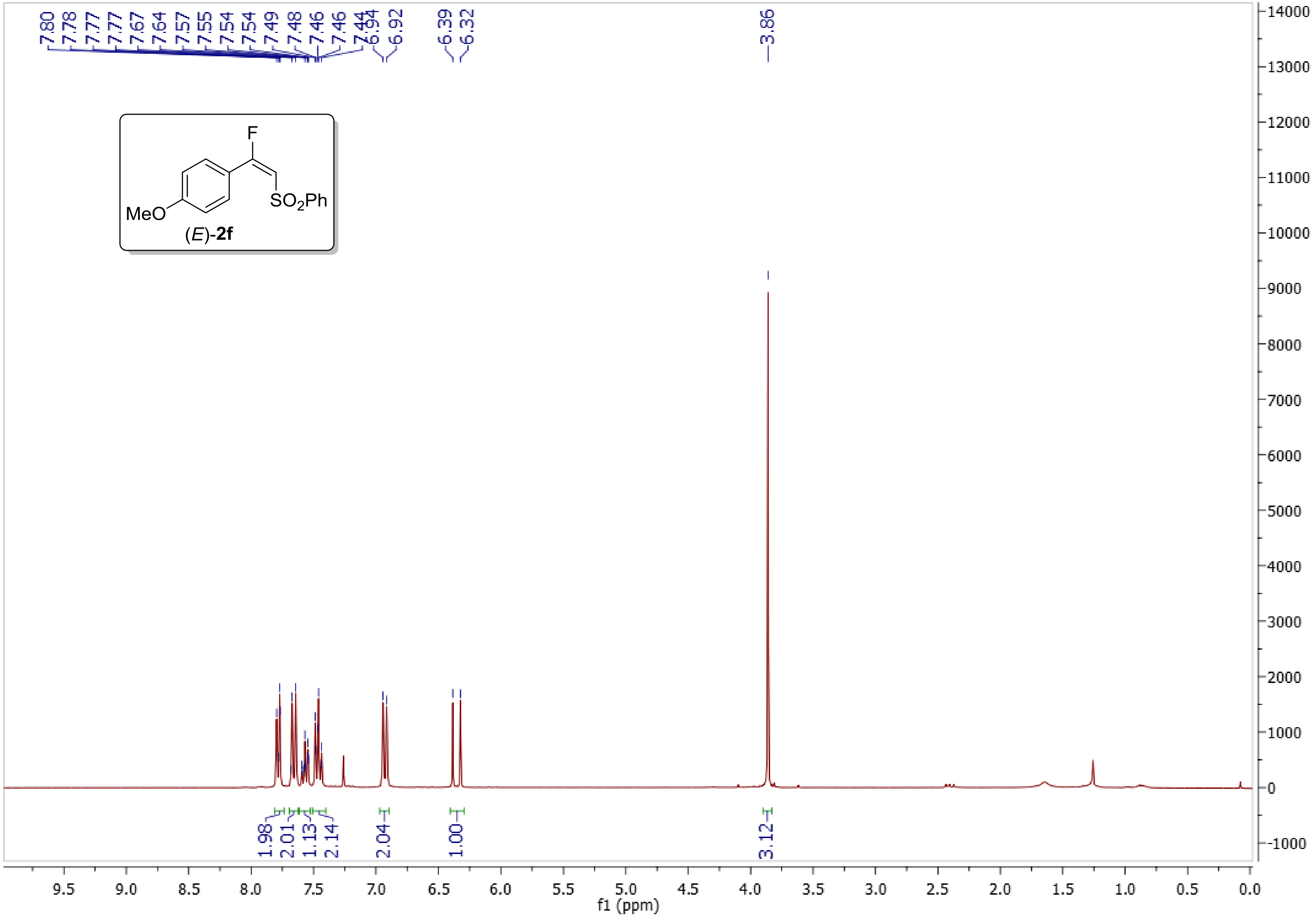

7.80
7.78
7.77
7.77
7.67
7.64
7.57
7.55
7.54
7.54
7.49
7.48
7.46
7.46
7.44
6.94
6.92
6.39
6.32
3.86
F
MeO
SO2Ph
(E)-2f
1.98
2.01
1.13
2.14
2.04
1.00
3.12
f1 (ppm)

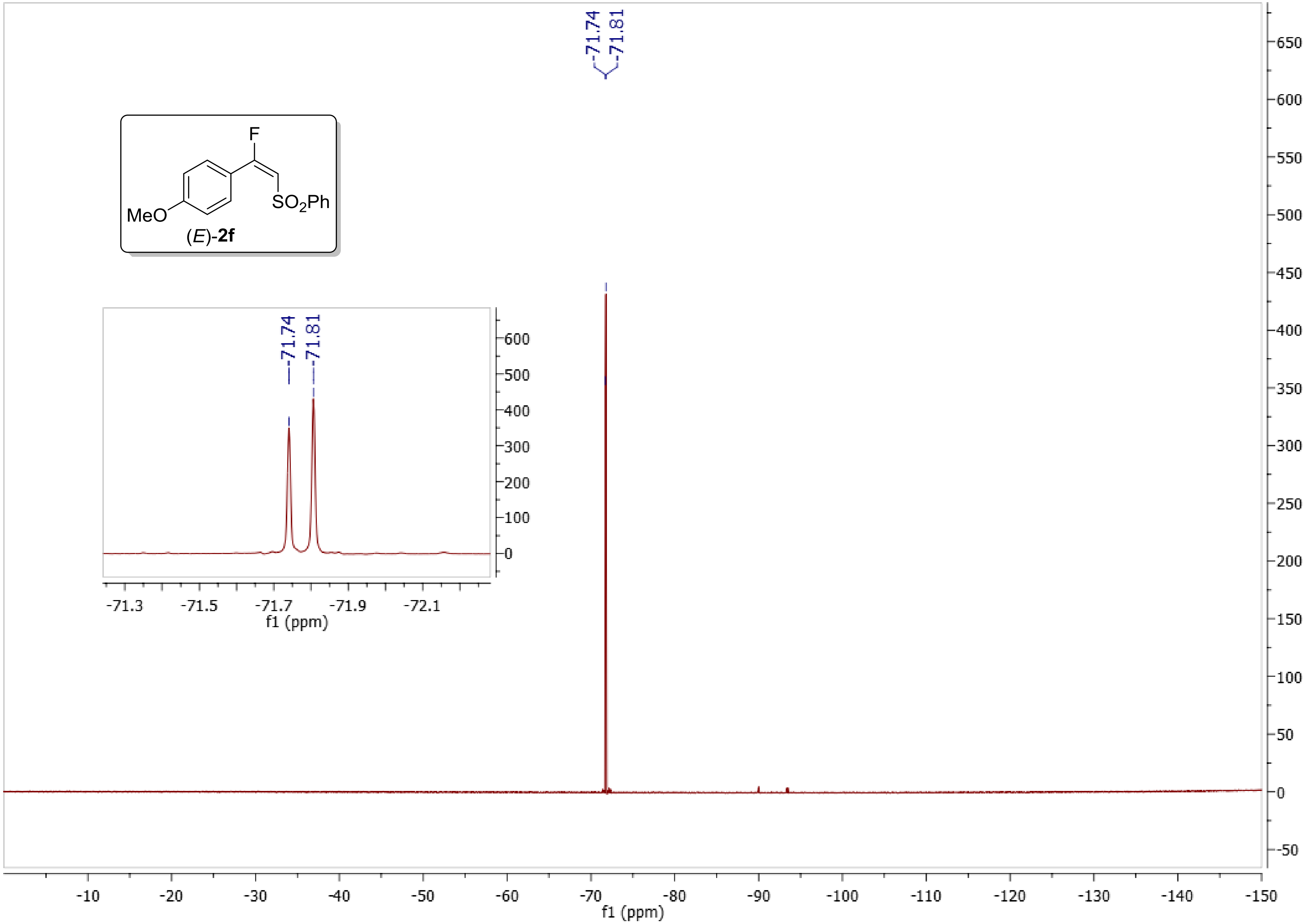

-71.74
-71.81
F
MeO
SO_2Ph
(*E*)-**2f**
-71.3
-71.5
-71.7
-71.9
-72.1
f1 (ppm)
-10
-20
-30
-40
-50
-60
-70
-80
-90
-100
-110
-120
-130
-140
-150
f1 (ppm)

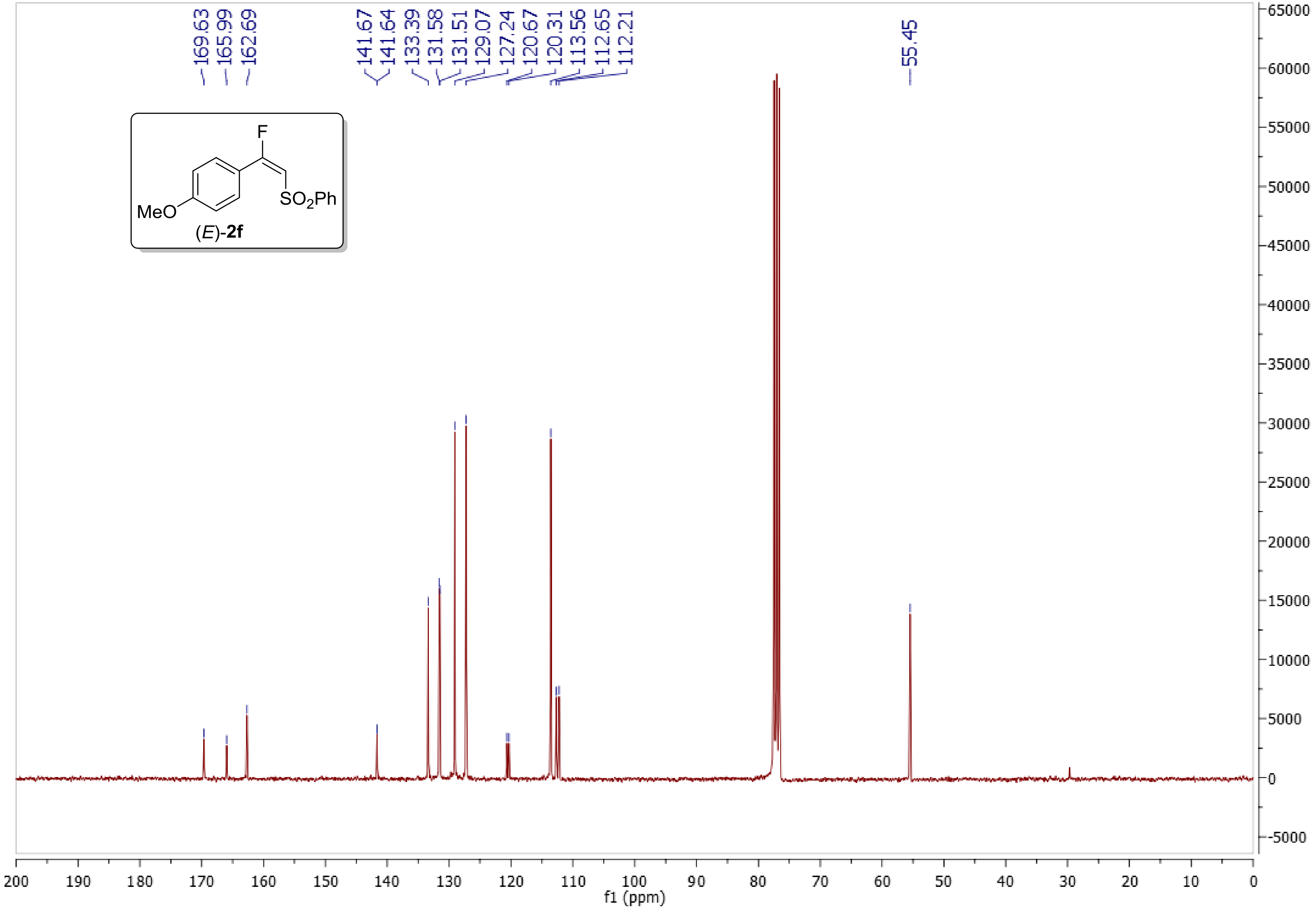
169.63
165.99
162.69
141.67
141.64
133.39
131.58
131.51
129.07
127.24
120.67
120.31
113.56
112.65
112.21
55.45
F
MeO
SO2Ph
(E)-2f
f1 (ppm)

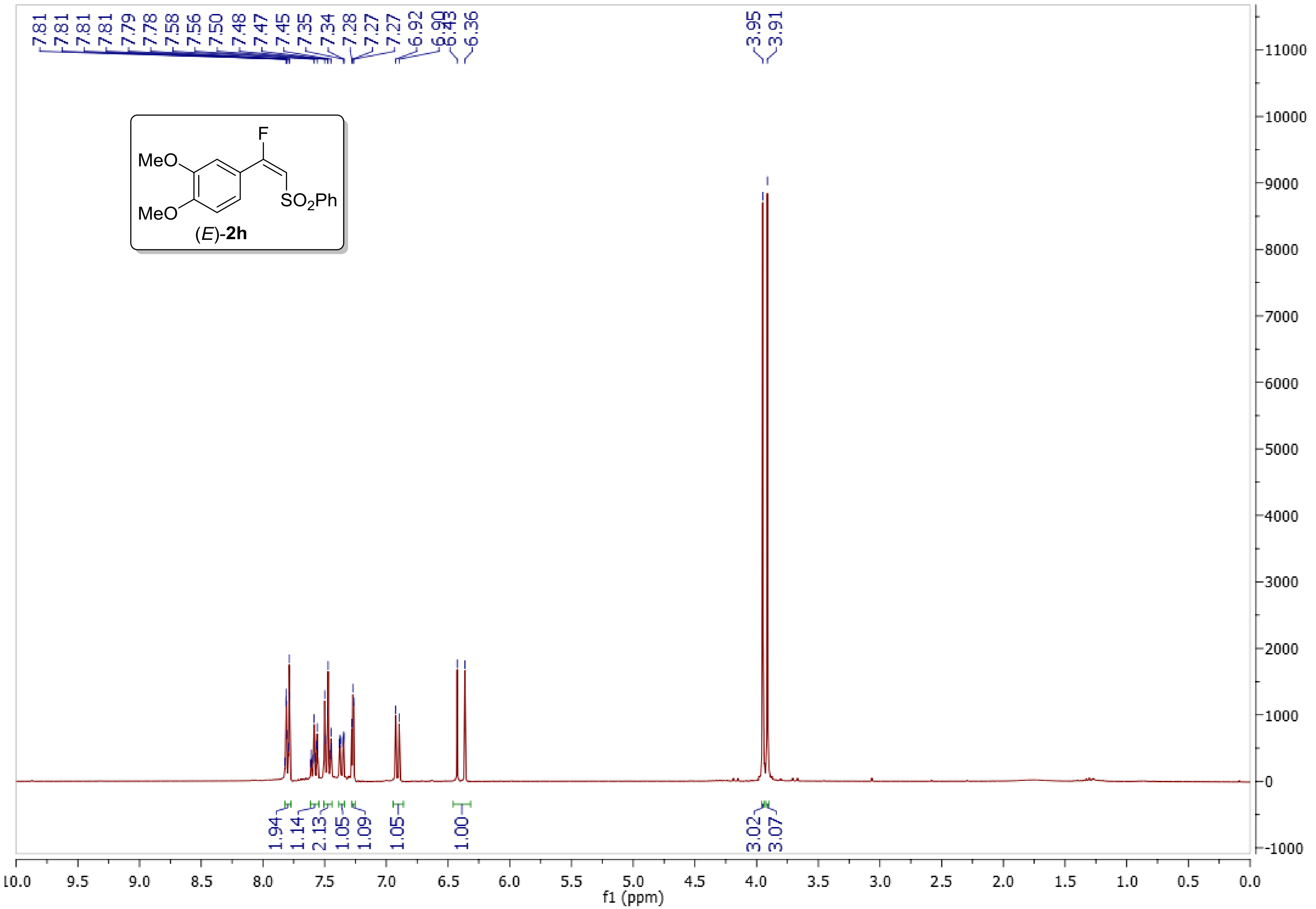
7.81
7.81
7.81
7.81
7.79
7.78
7.58
7.56
7.50
7.48
7.47
7.45
7.35
7.34
7.28
7.27
7.27
6.92
6.90
6.43
6.36
3.95
3.91
MeO
MeO
F
SO2Ph
(E)-2h
1.94
1.14
2.13
1.05
1.09
1.05
1.00
3.02
3.07
f1 (ppm)

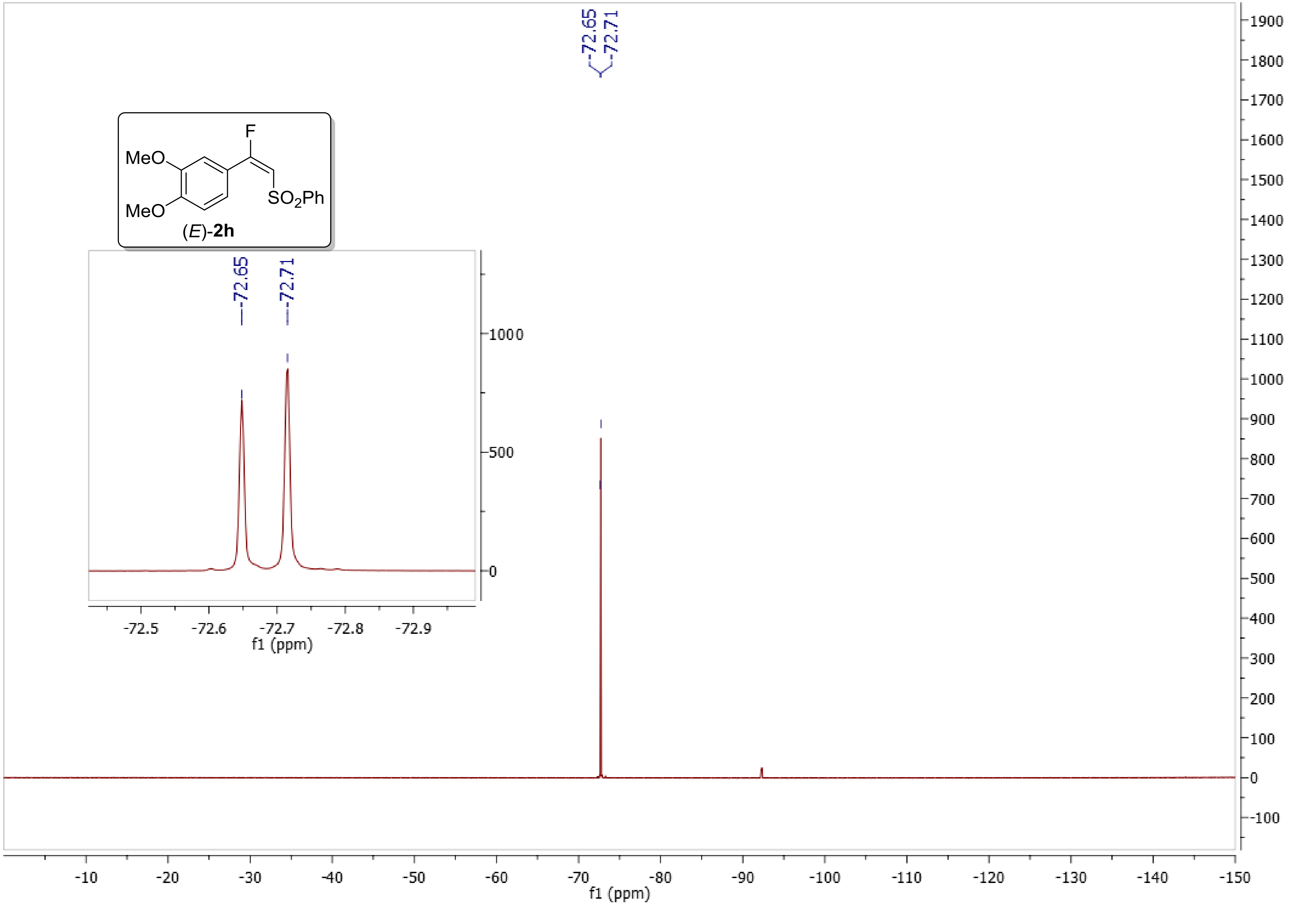

-72.65
-72.71
F
MeO
MeO
SO2Ph
(E)-2h
-72.5
-72.6
-72.7
-72.8
-72.9
f1 (ppm)
1000
500
0
1900
1800
1700
1600
1500
1400
1300
1200
1100
1000
900
800
700
600
500
400
300
200
100
0
-100
-10
-20
-30
-40
-50
-60
-70
-80
-90
-100
-110
-120
-130
-140
-150
f1 (ppm)

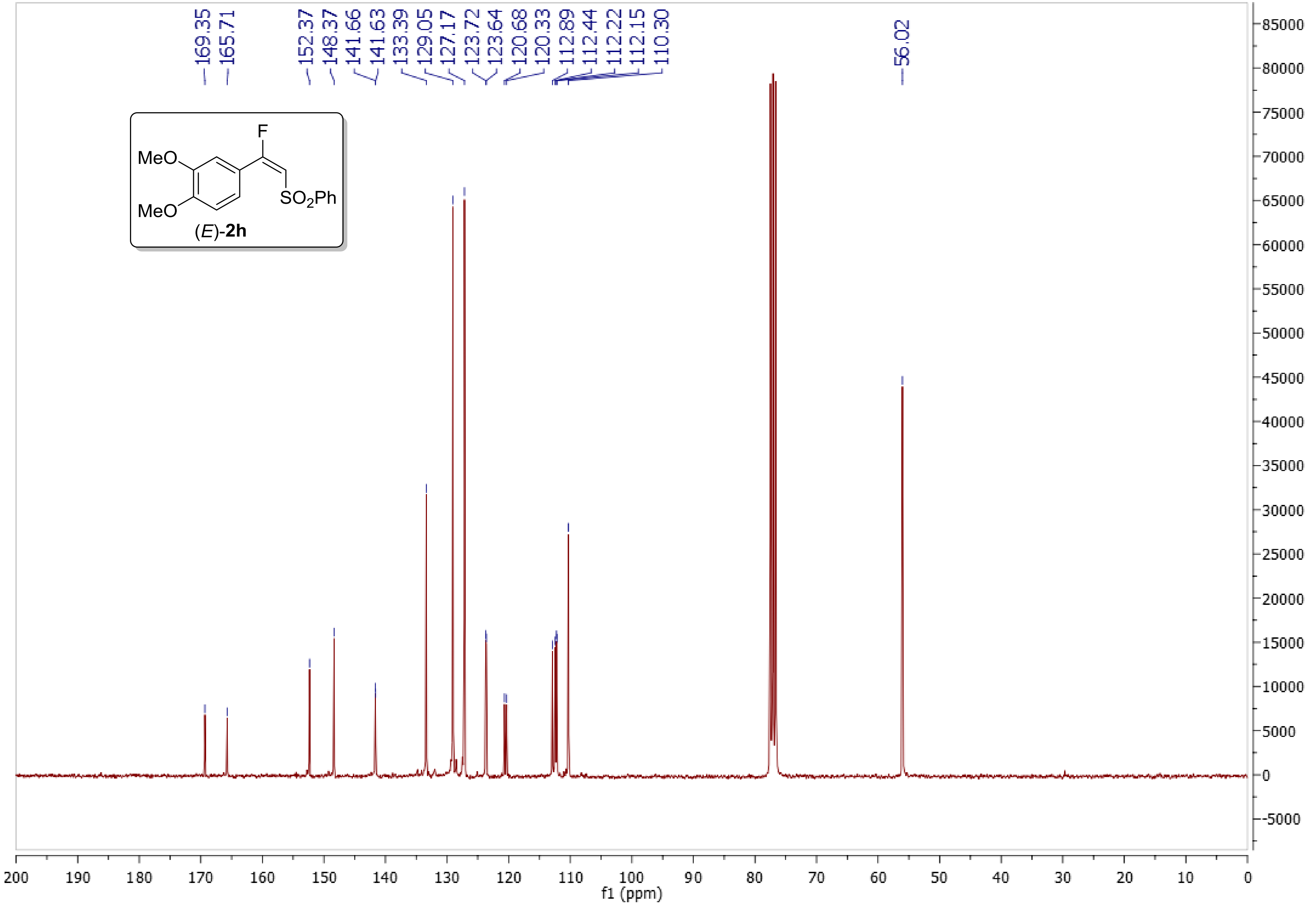
169.35
165.71
152.37
148.37
141.66
141.63
133.39
129.05
127.17
123.72
123.64
120.68
120.33
112.89
112.44
112.22
112.15
110.30
56.02
F
MeO
MeO
SO2Ph
(E)-2h
85000
80000
75000
70000
65000
60000
55000
50000
45000
40000
35000
30000
25000
20000
15000
10000
5000
0
-5000
200
190
180
170
160
150
140
130
120
110
100
90
80
70
60
50
40
30
20
10
0
f1 (ppm)

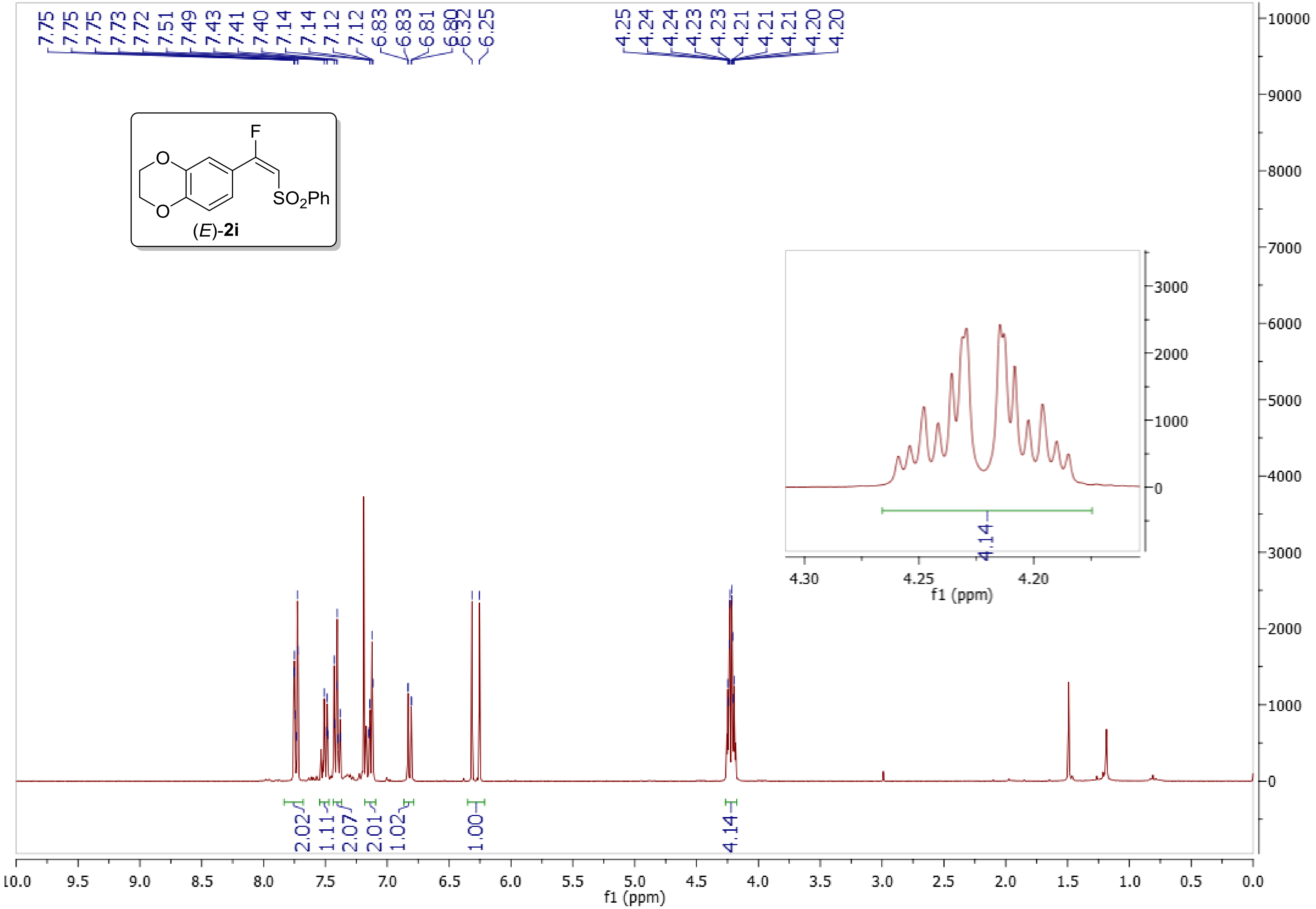

7.75 7.75 7.75 7.73 7.72 7.51 7.49 7.43 7.41 7.40 7.14 7.14 7.12 7.12 6.83 6.83 6.81 6.80 6.32 6.25
4.25 4.24 4.24 4.23 4.23 4.21 4.21 4.21 4.20 4.20
F
O
O
SO2Ph
(E)-2i
4.14
4.30 4.25 4.20
f1 (ppm)
2.02 1.11 2.07 2.01 1.02 1.00 4.14
f1 (ppm)

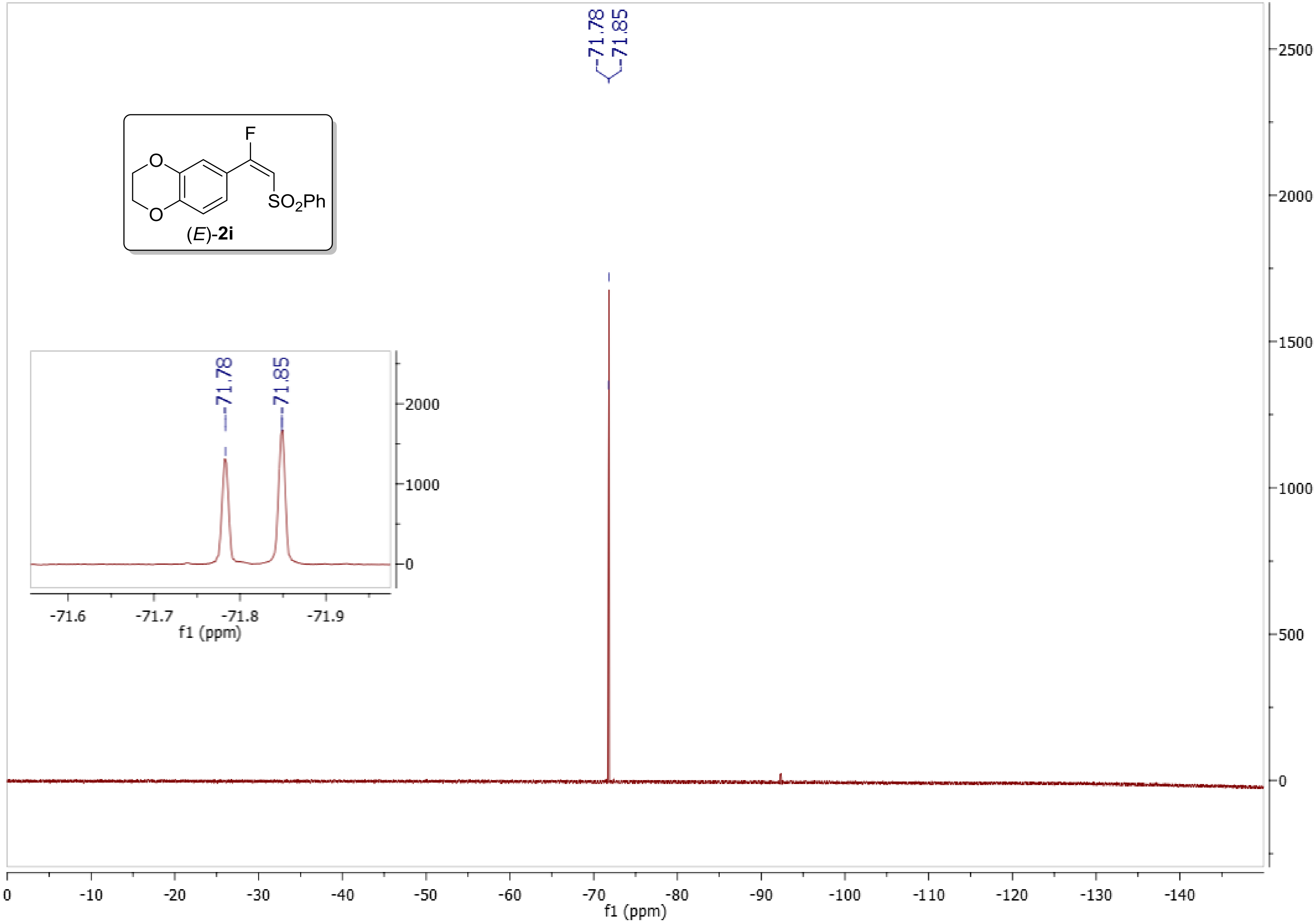

-71.78
-71.85
F
O
O
SO2Ph
(E)-2i
-71.78
-71.85
2000
1000
0
-71.6
-71.7
-71.8
-71.9
f1 (ppm)
2500
2000
1500
1000
500
0
0
-10
-20
-30
-40
-50
-60
-70
-80
-90
-100
-110
-120
-130
-140
f1 (ppm)

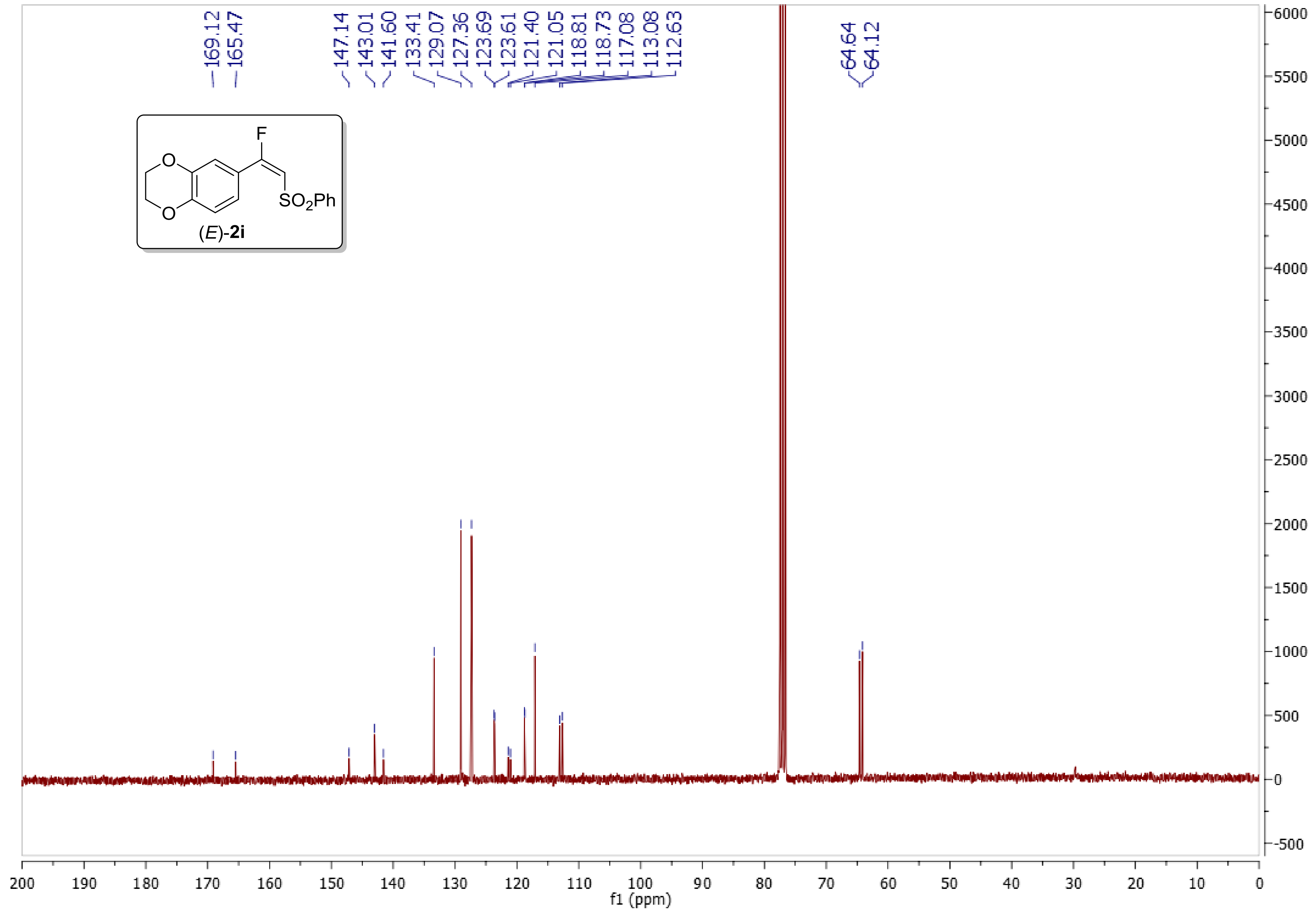
169.12
165.47
147.14
143.01
141.60
133.41
129.07
127.36
123.69
123.61
121.40
121.05
118.81
118.73
117.08
113.08
112.63
64.64
64.12
F
O
O
SO2Ph
(E)-2i
f1 (ppm)

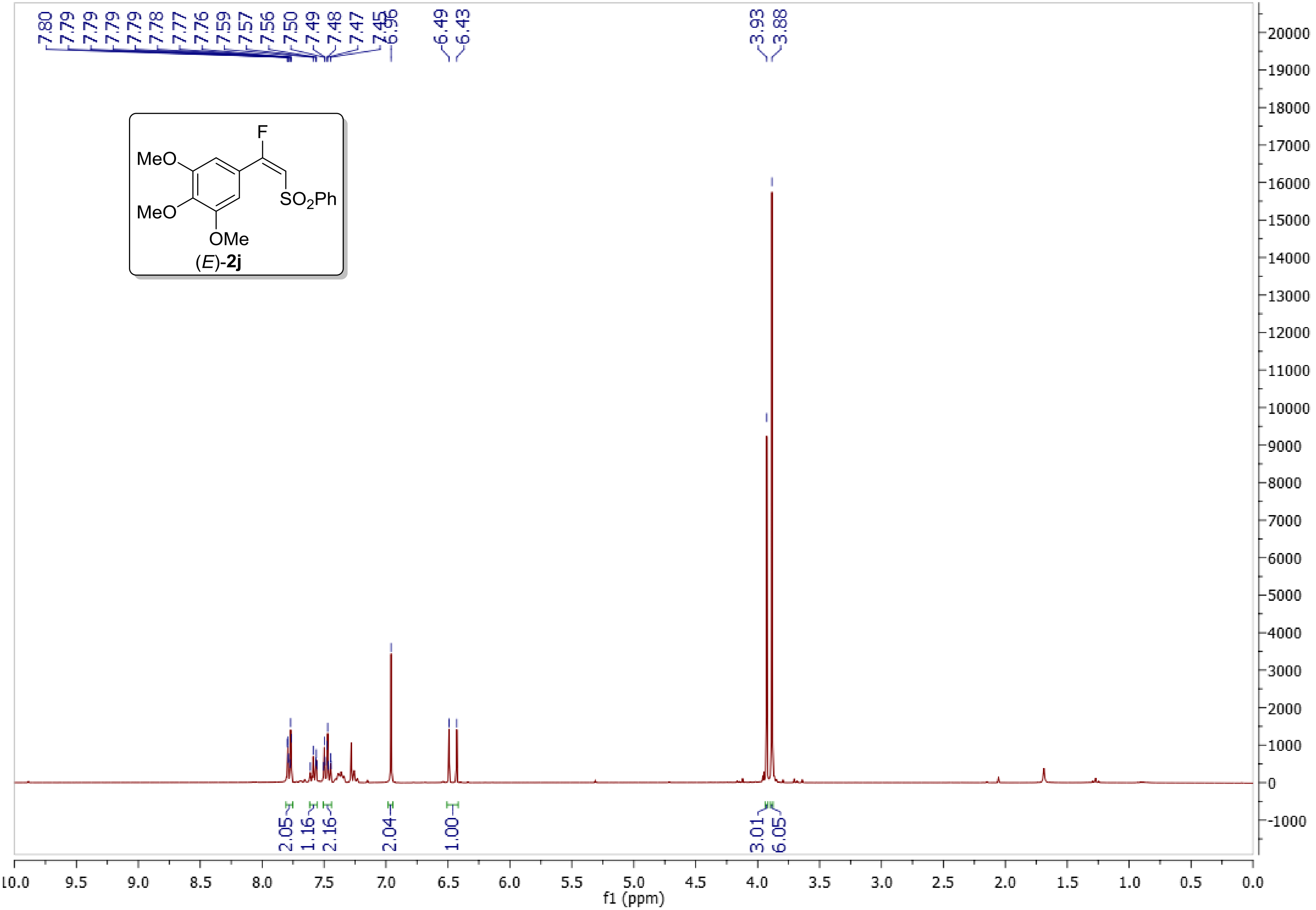

7.80 7.79 7.79 7.79 7.79 7.78 7.77 7.76 7.59 7.57 7.56 7.50 7.49 7.48 7.47 7.45 6.96
6.49 6.43
3.93 3.88
F
MeO
MeO
OMe
SO2Ph
(E)-2j
2.05 1.16 2.16 2.04 1.00 3.01 6.05
f1 (ppm)

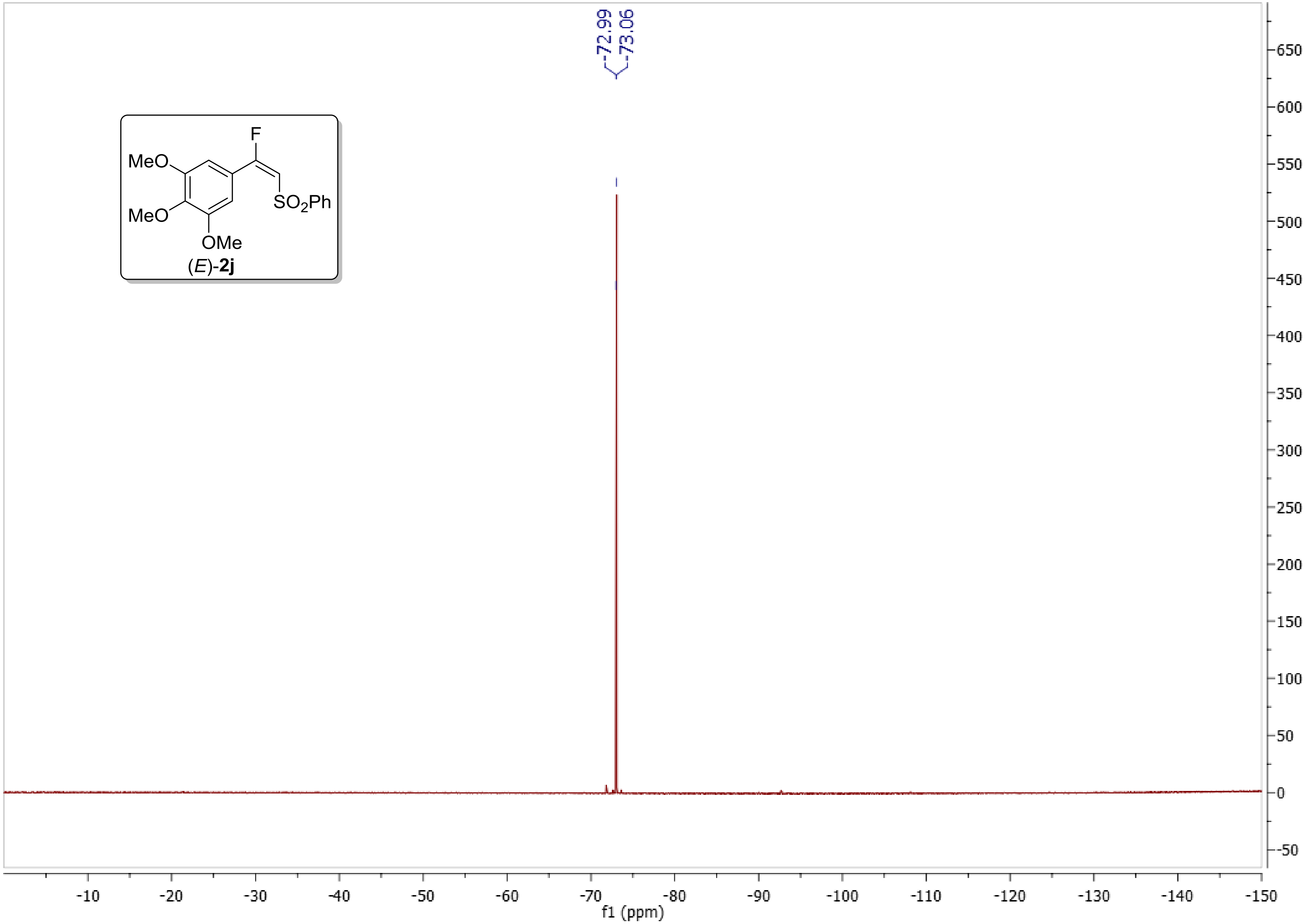

-72.99
-73.06
MeO
MeO
OMe
F
SO2Ph
(E)-2j
f1 (ppm)

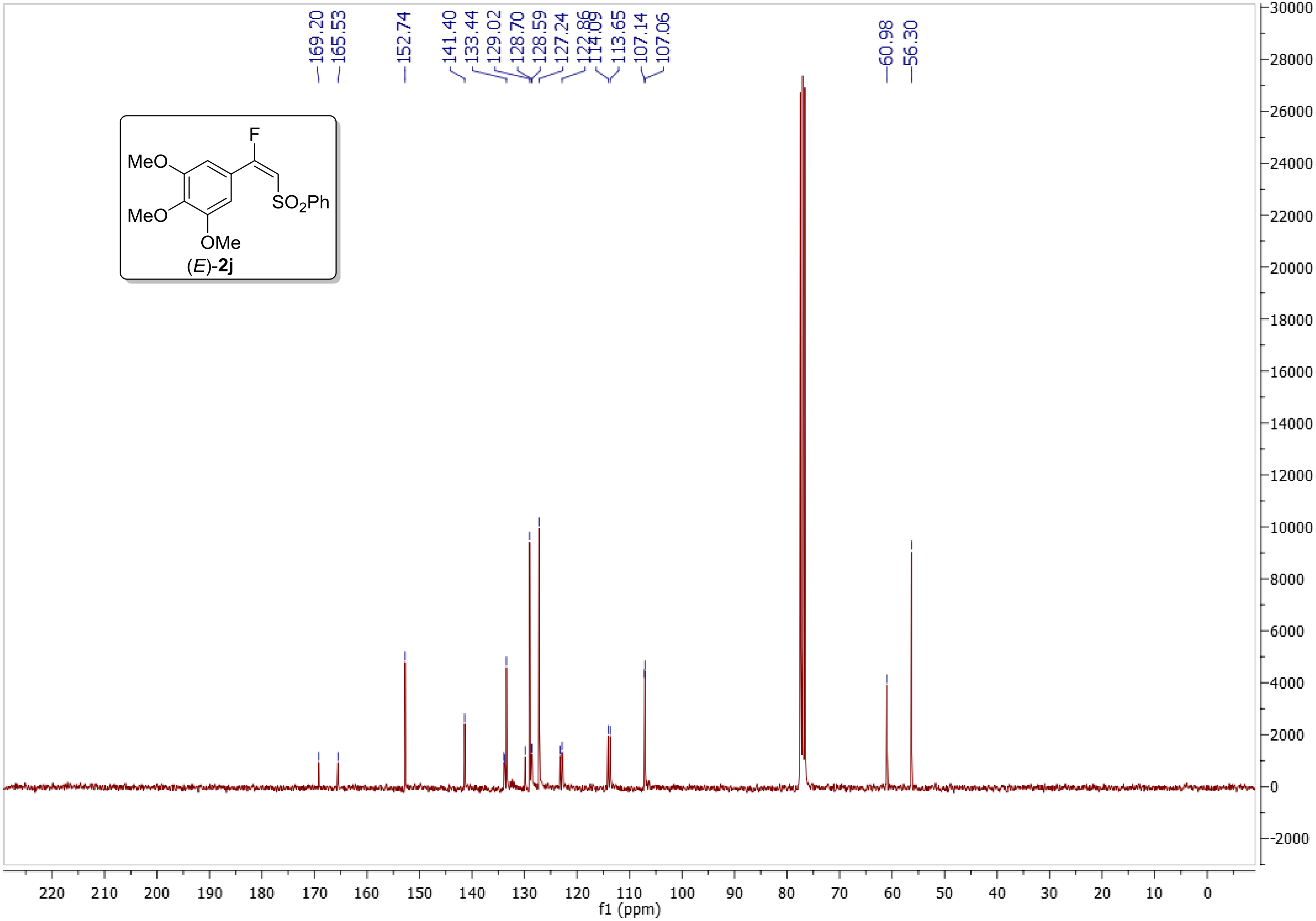

169.20
165.53
152.74
141.40
133.44
129.02
128.70
128.59
127.24
122.86
114.09
113.65
107.14
107.06
60.98
56.30
F
MeO
MeO
OMe
SO_2Ph
(*E*)-**2j**
f1 (ppm)

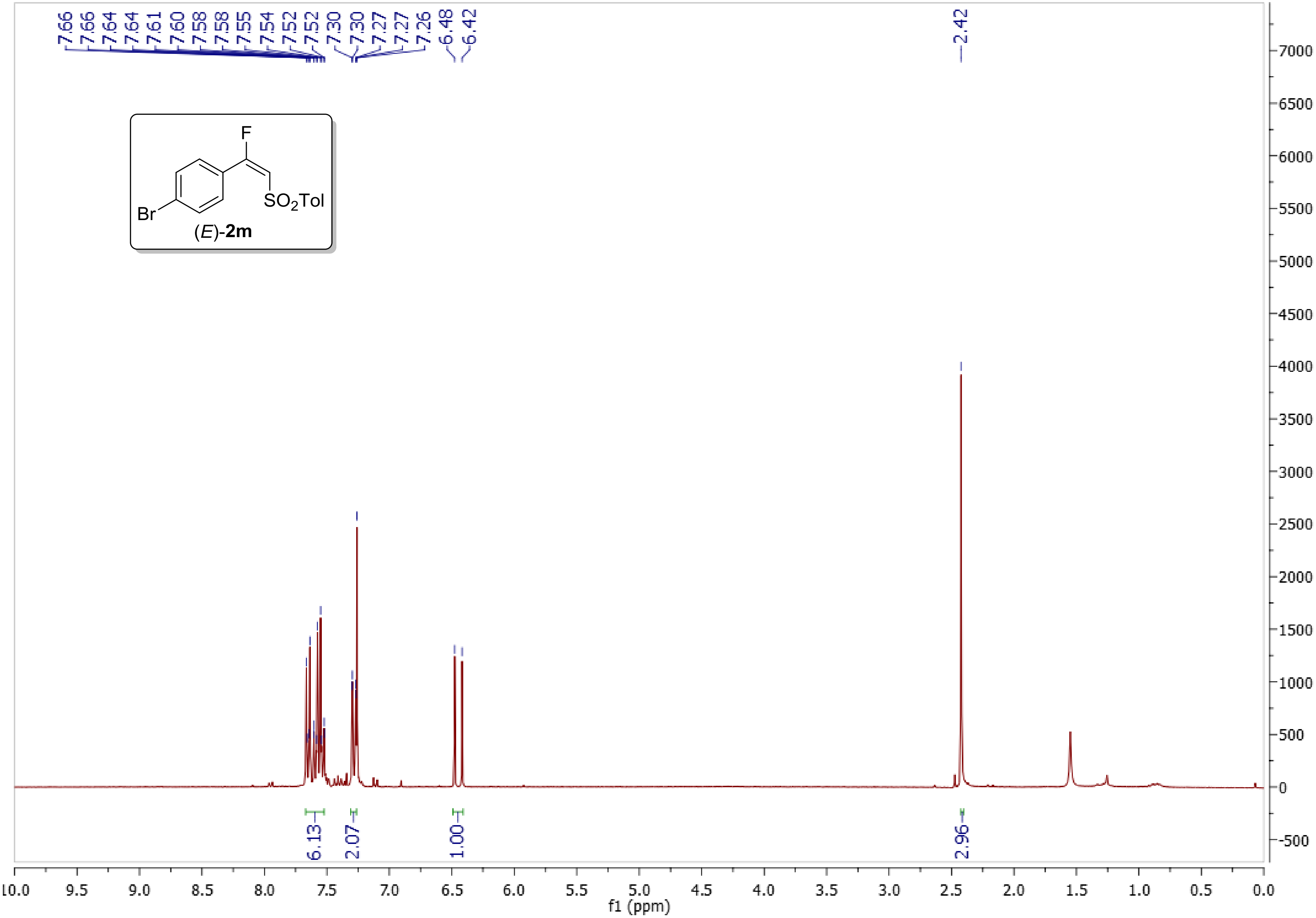
7.66 7.66 7.64 7.64 7.61 7.60 7.58 7.58 7.55 7.54 7.52 7.52 7.30 7.30 7.27 7.27 7.26 6.48 6.42
2.42
F
Br
SO2Tol
(E)-2m
6.13
2.07
1.00
2.96
7000 6500 6000 5500 5000 4500 4000 3500 3000 2500 2000 1500 1000 500 0 -500
10.0 9.5 9.0 8.5 8.0 7.5 7.0 6.5 6.0 5.5 5.0 4.5 4.0 3.5 3.0 2.5 2.0 1.5 1.0 0.5 0.0
f1 (ppm)

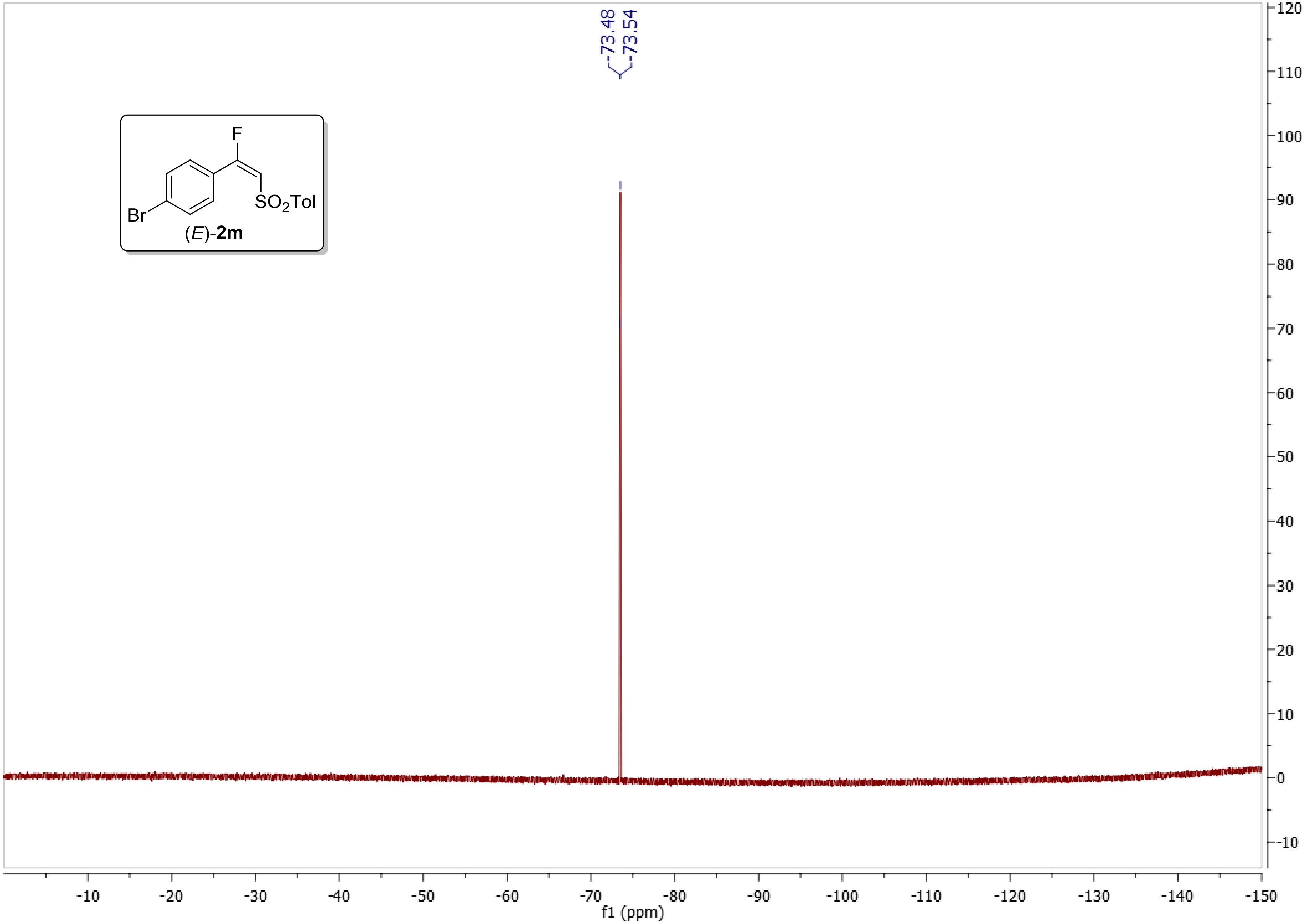

-73.48
-73.54
F
Br
SO$_2$Tol
(*E*)-**2m**
f1 (ppm)

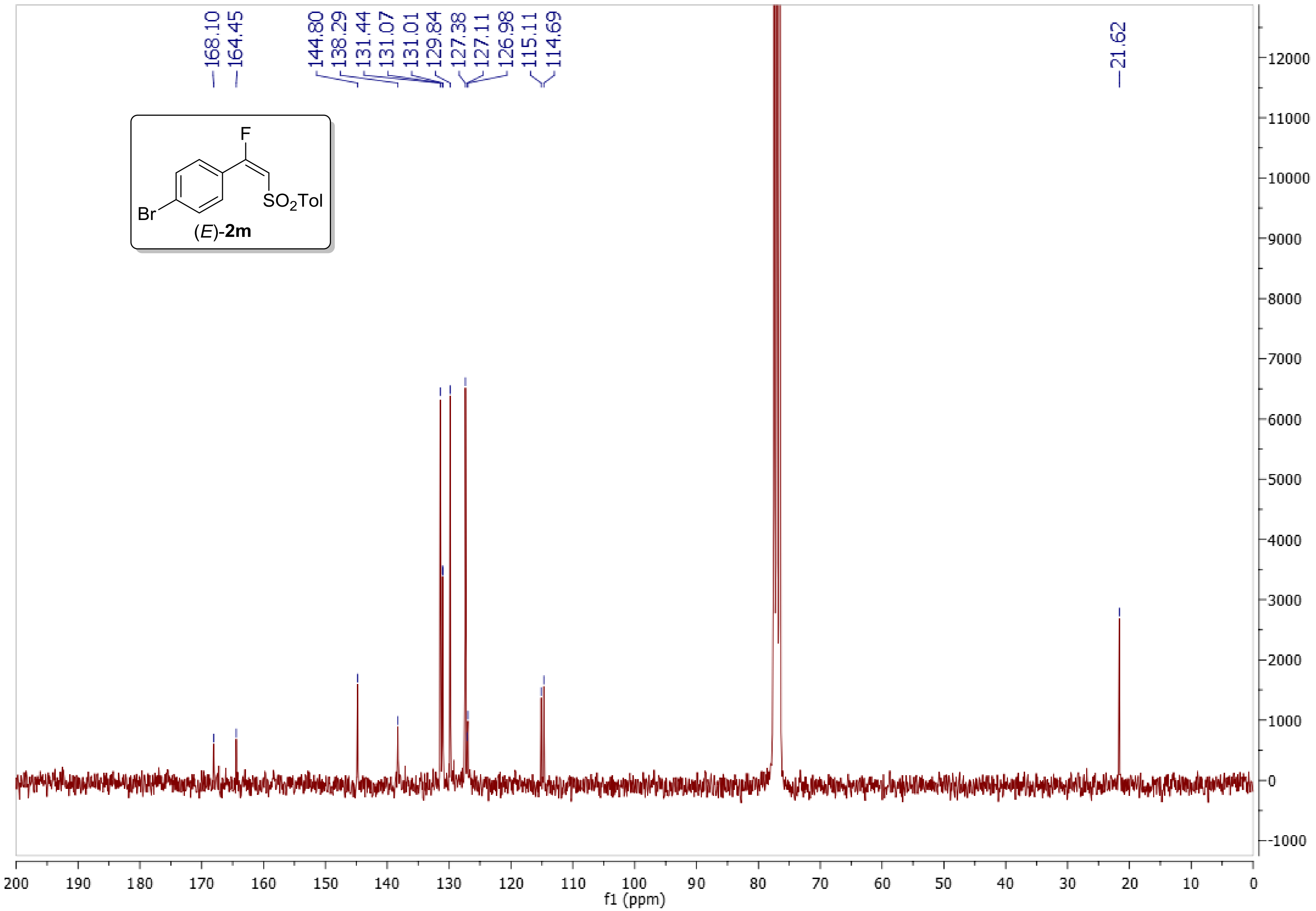

168.10
164.45
144.80
138.29
131.44
131.07
131.01
129.84
127.38
127.11
126.98
115.11
114.69
21.62
F
Br
SO2Tol
(E)-2m
f1 (ppm)

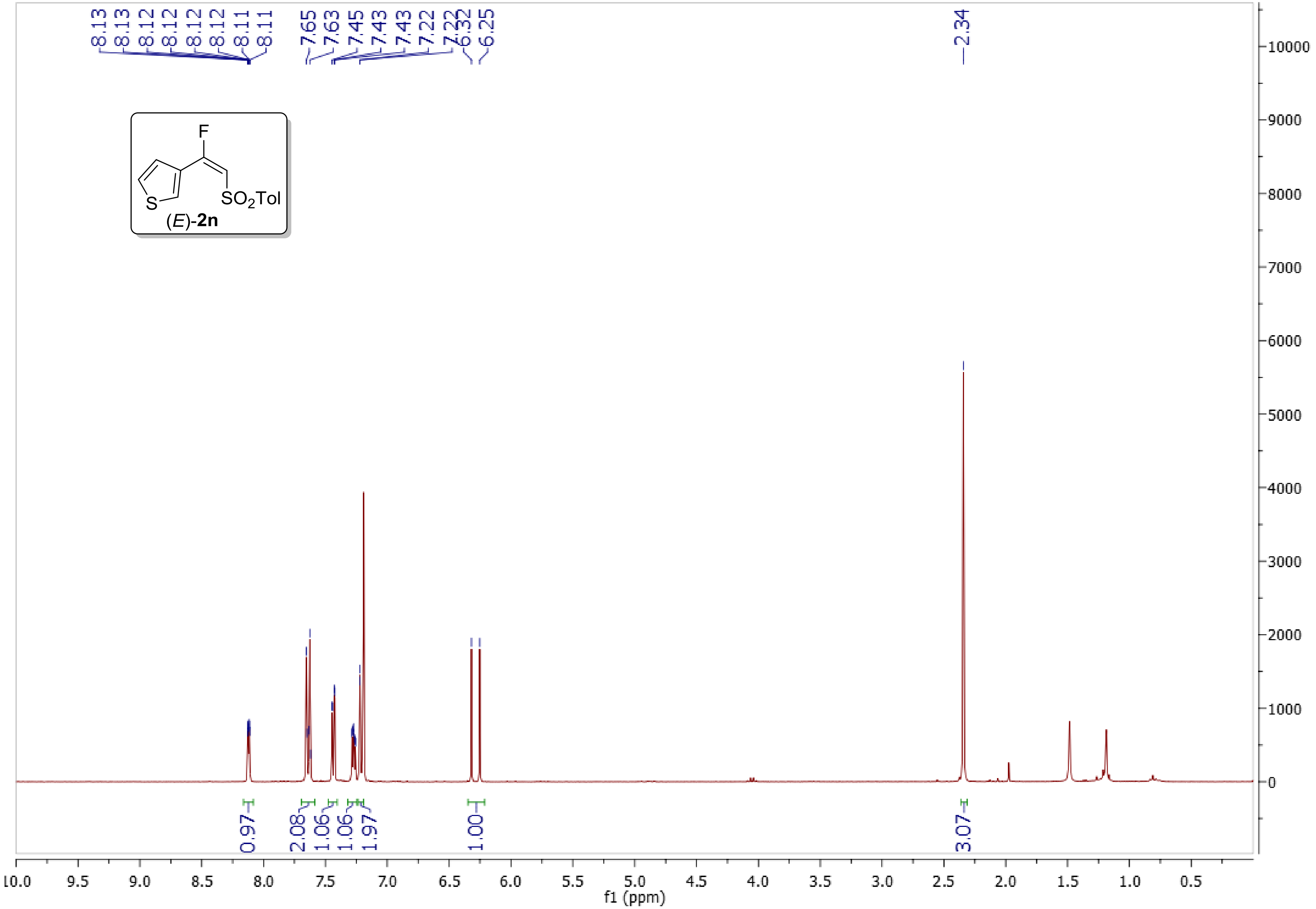

8.13
8.13
8.12
8.12
8.12
8.12
8.11
8.11
7.65
7.63
7.45
7.43
7.43
7.22
7.22
6.32
6.25
2.34
F
S
SO2Tol
(E)-2n
0.97
2.08
1.06
1.06
1.97
1.00
3.07
f1 (ppm)

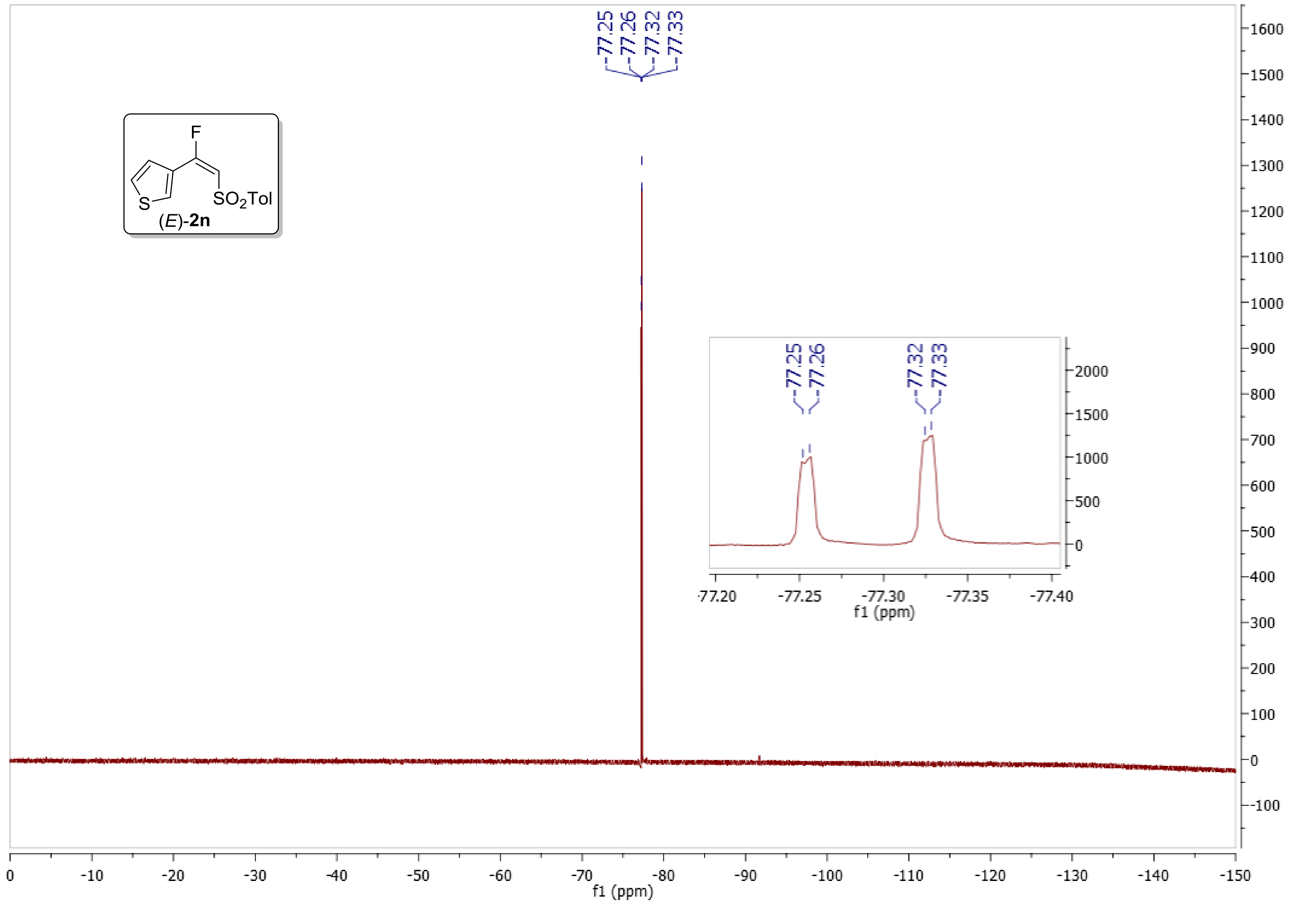

-77.25
-77.26
-77.32
-77.33
F
S
SO2Tol
(E)-2n
-77.20
-77.25
-77.30
-77.35
-77.40
f1 (ppm)
2000
1500
1000
500
0
1600
1500
1400
1300
1200
1100
1000
900
800
700
600
500
400
300
200
100
0
-100
0
-10
-20
-30
-40
-50
-60
-70
-80
-90
-100
-110
-120
-130
-140
-150
f1 (ppm)

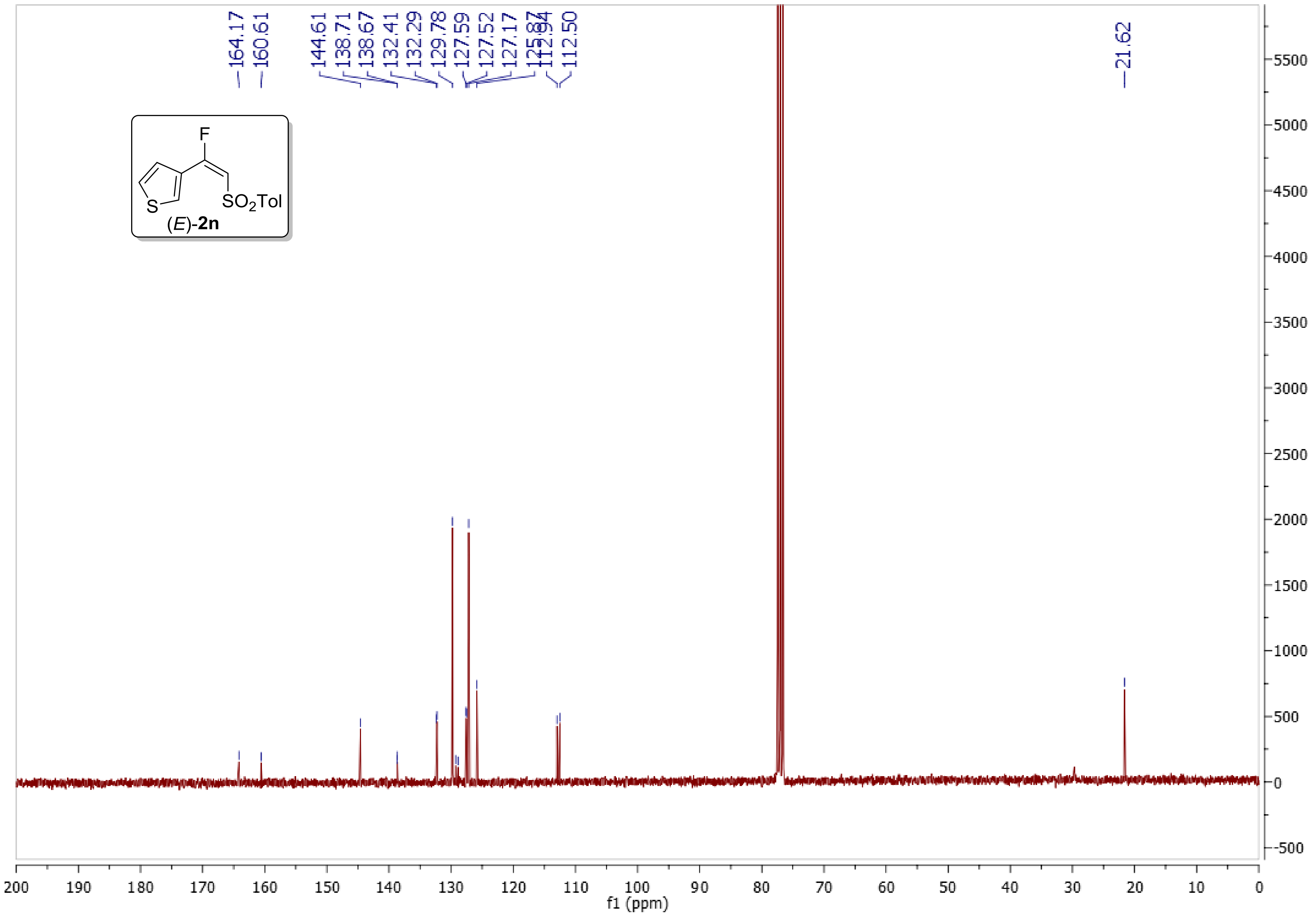
164.17
160.61
144.61
138.71
138.67
132.41
132.29
129.78
127.59
127.52
127.17
125.87
112.94
112.50
21.62
F
S
SO2Tol
(E)-2n
f1 (ppm)
